# Discovery of an endogenous foamy virus in primitive ruminant chevrotains

**DOI:** 10.1128/spectrum.02090-23

**Published:** 2023-08-15

**Authors:** Xiaojing Wang, Jie Cui

**Affiliations:** 1 CAS Key Laboratory of Molecular Virology & Immunology, Shanghai Institute of Immunity and Infection, Chinese Academy of Sciences, Shanghai, China; 2 University of Chinese Academy of Sciences, Beijing, China; 3 Laboratory for Marine Biology and Biotechnology, Pilot National Laboratory for Marine Science and Technology, Qingdao, China; Changchun Veterinary Research Institute, Changchun, China

**Keywords:** endogenous foamy virus, phylogenetic analysis, virus evolution, retroviruses

## Abstract

Foamy viruses (FVs) are a class of complex retroviruses that could lead to persistent infections in specific species. FVs have two evolutionary characteristics: the first is co-evolution with the host, and the second is difficulty going through the endogenization process. To date, in mammals, only a few species (aye-aye, sloths, and cape golden mole) have been found to have endogenous forms of FVs. In this study, we identified a novel endogenous FV named TraEFV in the genomes of two species of even-toed ungulate chevrotain (genus Tragulus, family Tragulidae) known as mouse-deer. Phylogenetic analysis clustered TraEFVs with an exogenous FV isolated from feline but not with those isolated from cow and horse, and such inconsistent virus-host relationships reflect their complex evolutionary history. Interestingly, TraEFVs could be divided into two lineages, suggesting that TraEFV invaded these hosts at least twice during ancient times. Finally, the molecular clock estimates that TraEFV is approximately 20 million years old, suggesting its ancient nature. Our findings enrich the host taxonomy of spumaretroviruses.

**IMPORTANCE:**

Foamy viruses (FV) are complex retroviruses that generally codiverge with their hosts. We identified a novel endogenous FV in the genomes of two mouse-deer species, the first endogenous FV found in Artiodactyla. The phylogenetic inconsistency of viruses and hosts suggested that the viruses might have emerged from cross-species transmission in the past. These findings indicate that ancient FVs may have had a wider range of hosts that remain to be expanded.

## INTRODUCTION

Spumavirinae is a distinct subfamily of Retroviridae that consists of only one genus, the foamy virus (FV), also called spumaretrovirus. Since 1954 (1), foamy viruses have been isolated from tissues or cells of various mammals, including non-human primates (2 – 5), horses (6), cats (7), bats (8), and cows (9 – 11). These extant FVs exhibit a co-evolutionary history with their hosts over a long-term course (12). However, these extant viruses represent a limited variety of viruses, which limit our understanding of the evolutionary history of foamy viruses.

Retroviruses, including foamy viruses, have the potential to undergo endogenization and leave viral remnants within the host genomes. Occasionally, retroviruses can integrate into the germ line and become heritable viral elements, named endogenous retroviruses (ERVs). These endogenous retroviruses, known as “fossil viruses,” are important molecular tools for revealing the evolutionary history of retroviruses. To date, endogenous foamy viruses (EFVs) were identified in the five major classes of vertebrates (mammals [12 – 15], birds [16, 17], reptiles [16, 18], amphibians [19, 20], and fish[(19, 21 – 23]). The increasing number of EFVs extended the host range of FVs and extended the estimated age of FVs to approximately 400 million years (19), supporting the extremely ancient origin of these viruses.

Compared with the exogenous FVs, the evolutionary pattern of EFVs appears to be much more complex. Cross-species transmission events have been observed in EFVs of prosimian aye-aye (12, 14), amphibians (20), and lobe-finned fish (23), indicating ancient FVs are capable of infecting distantly related species. In addition, the diversity and quantity of EFVs harbored by terrestrial animals (mammals, birds, reptiles) are smaller than those carried by amphibians and fish. In mammals, in particular, remnants of ancient foamy viruses have been identified only in the genomes of the aye-aye (14), sloths (13), and the cape golden mole (15) of Eutheria. The addition of genomic data sets may provide new clues to the evolutionary history of mammalian endogenous foamy viruses. In this study, we conducted a comprehensive screening of 524 available mammalian genomes and successfully identified a novel endogenous foamy virus in the genus Tragulus, family Tragulidae, for the first time in Artiodactyla. Furthermore, the phylogenetic and genomic analyses showed that TraEFV had undergone a complex evolutionary history.

## RESULTS

### Discovery foamy viral elements in *Tragulus javanicus* and *T. kanchil*

**Fig 1:**
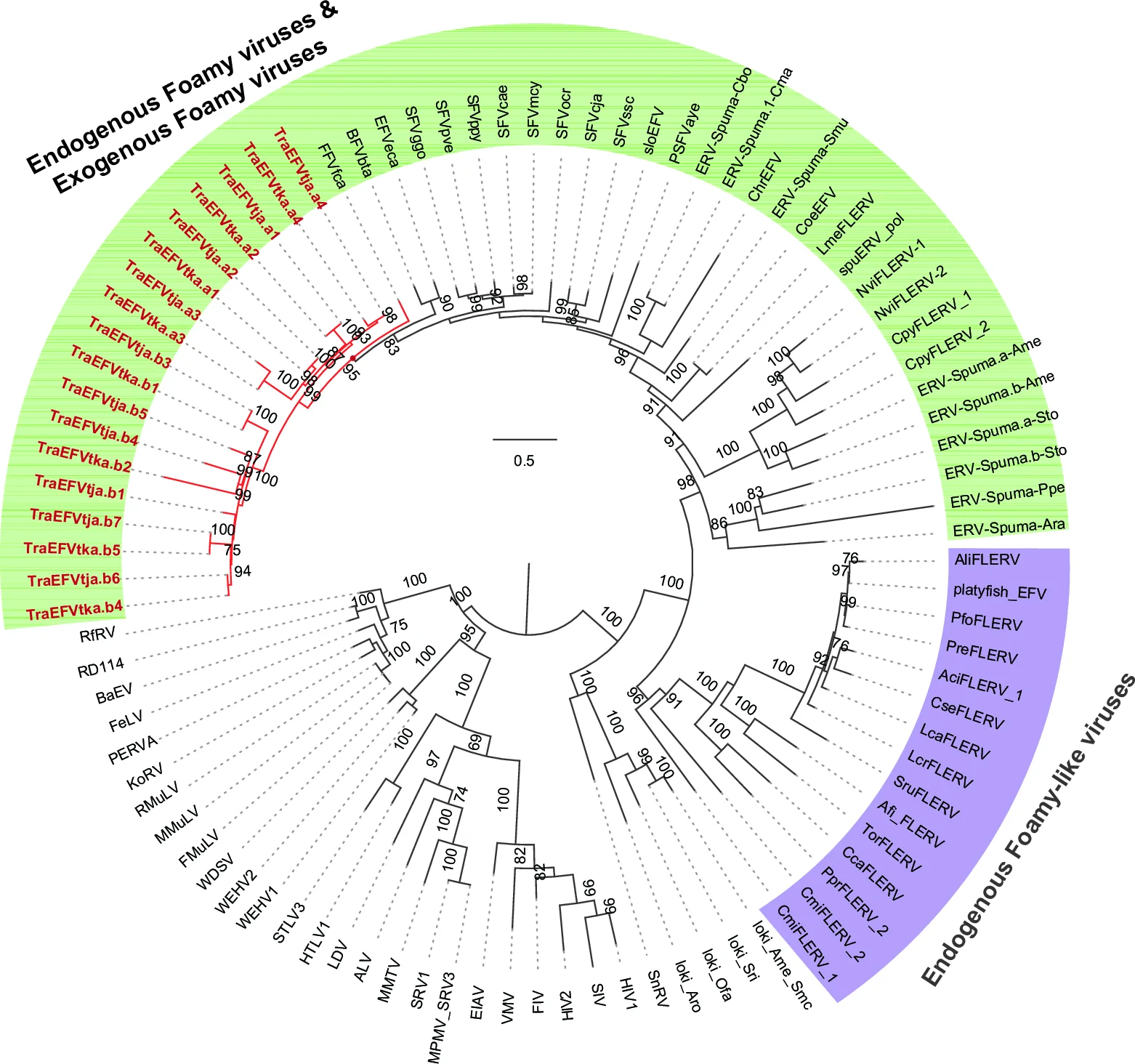
Unrooted phylogeny of TraEFVs and other retroviruses. The tree was inferred from reverse transcriptase (RT) protein alignment. The node robustness was evaluated through ultrafast bootstrap with 1,000 replicates. The newly identified EFVs in the Tragulus genus (TraEFVs) are labeled in red. The scale bar indicates the number of amino acid changes per site. Bootstrap values of <70% are not shown.

We screened 524 genome assemblies of mammals by using POL of mammalian exogenous and endogenous FVs as probes (Table S3). As expected, the endogenous forms of foamy viruses were rarely detected in mammals, and only seven species harbored endogenous foamy viral elements in their genomes. Five of these species (13 – 15) had been previously reported (Table S2). In addition, 20 EFVs have been identified in *Tragulus javanicus* (Java mouse-deer) and *T. kanchil* (lesser mouse-deer), both of which belong to the genus Tragulus and represent primitive ruminants. The RT phylogeny revealed that EFVs identified in the genus Tragulus clustered with mammalian FVs with robust support (bootstrap value of 98%) and were divided into two clades (Fig. 1). According to the topologies, two lineages of EFVs in *T. kanchil* were named TraEFVtka.an (*n* = 1–4) and TraEFVtka.bn (*n* = 1–5), and two lineages in *T. javanicus* were named TraEFVtja.an (*n* = 1–4) and TraEFVtja.bn (*n* = 1–7).

### Genomic characterization of TraEFV

**Fig 2:**
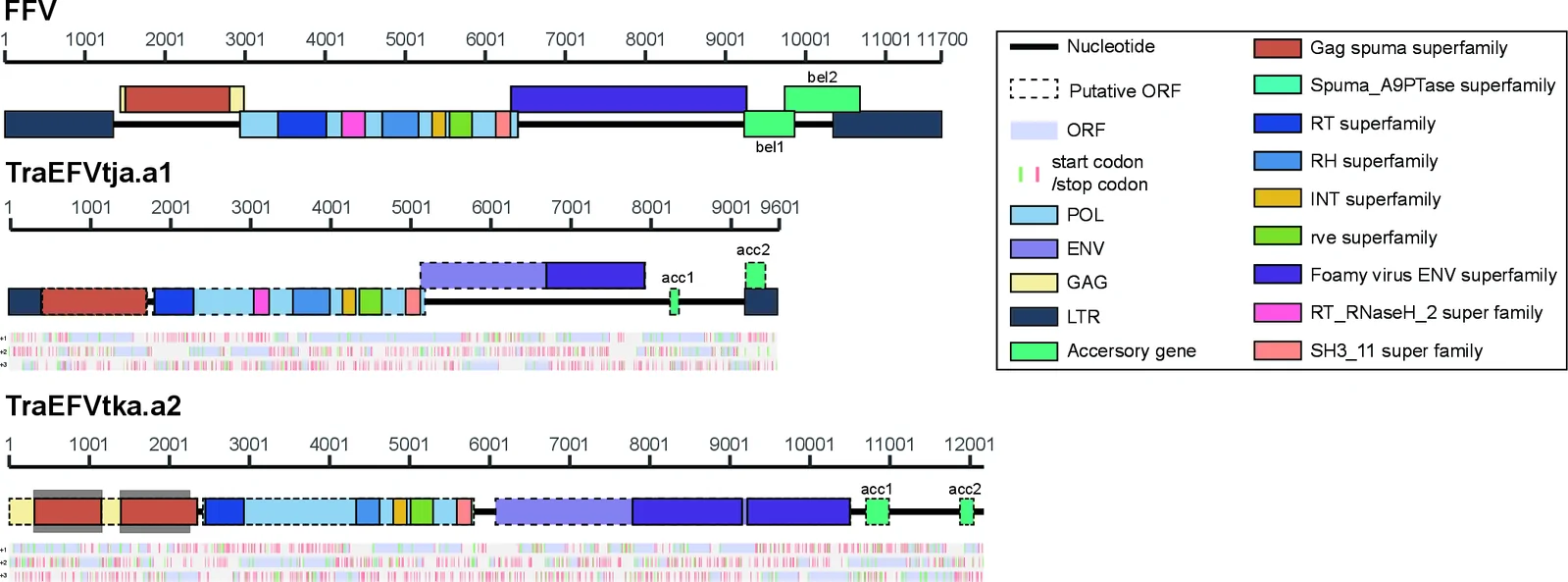
Genomic organization of FFV and TraEFVs. The genomes of feline foamy virus (FFV) and representative TraEFVs are drawn to scale using lines and boxes. The distributions of stop (red) and start (green) codons in three forward frames (+1, +2, +3; from top to bottom) are shown under a genomic schematic diagram for each copy of TraEFVs. Putative open reading frames (ORFs) are shown in dotted boxes and were used to determine viral coding regions. The predicted domain or regions that encode conserved proteins are represented by colored boxes. The repeated insertion fragments are indicated by gray boxes. LTR, long terminal repeat; GAG, group-specific antigen gene; POL, polymerase gene; ENV, envelope gene; RT, reverse transcriptase; RH, RNase H. INT, integrase. The *acc1* and *acc2* were the predicted accessory genes of TraEFVs.

To further characterize the genomic structure of TraEFVs, we identified pairwise long-terminal repeats (LTRs) in TraEFVs flanking sequences using LTR_harvest. One copy of TraEFVtja (TraEFVtja.a1) contained a pair of LTRs and was, therefore, considered to be a full-length TraEFV. All copies of TraEFVs had numerous pre-stop codon and frameshift mutations; therefore, no intact open reading frame (ORF) could be found (Fig. 2). Notably, for each copy of EFV identified in *T. kanchil* excluding TraEFVtka.b2, there was a highly similar EFV element found in *T. javanicus* (nucleic acid similarity: 95%–99%). By searching against Conserved Domain Database (CDD) using CD-Search, all conventional conserved domains of exogenous foamy viruses could be identified in TraEFVtja and TraEFVtka (Fig. 2), including the foamy viral-specific conserved domain [Gag_spuma superfamily (pfam03276), Foamy_virus_ENV superfamily (pfam03408), and Spuma_A9PTase superfamily (pfam03539)]. In addition, two accessory genes, *acc1* and *acc2*, were identified in TraEFVtja.a and TraEFVtka.a using tBLASTn (Table S4). However, we failed to identify the primer binding site (PBS) and internal promoter (TATAAAA) of TraEFVtja.a1. As previously described, PBS and the internal promoter of TraEFVtja may be unrecognizable due to the numerous mutations.

Notably, all copies of TraEFVtka contained repeated insertions of foamy viral fragments (Fig. 2). These insertions led to the existence of multiple identical regions (>90%) in TraEFVtka (e.g., the 2 Gag_spuma superfamily domains in TraEFVtka.a1, *gag-gag-pol*). Importantly, these fragments were regularly inserted next to another identical sequence. Thus, these repeated insertions may be artifacts due to genomic assembly errors.

### Phylogenetic analysis of TraEFV

**Fig 3:**
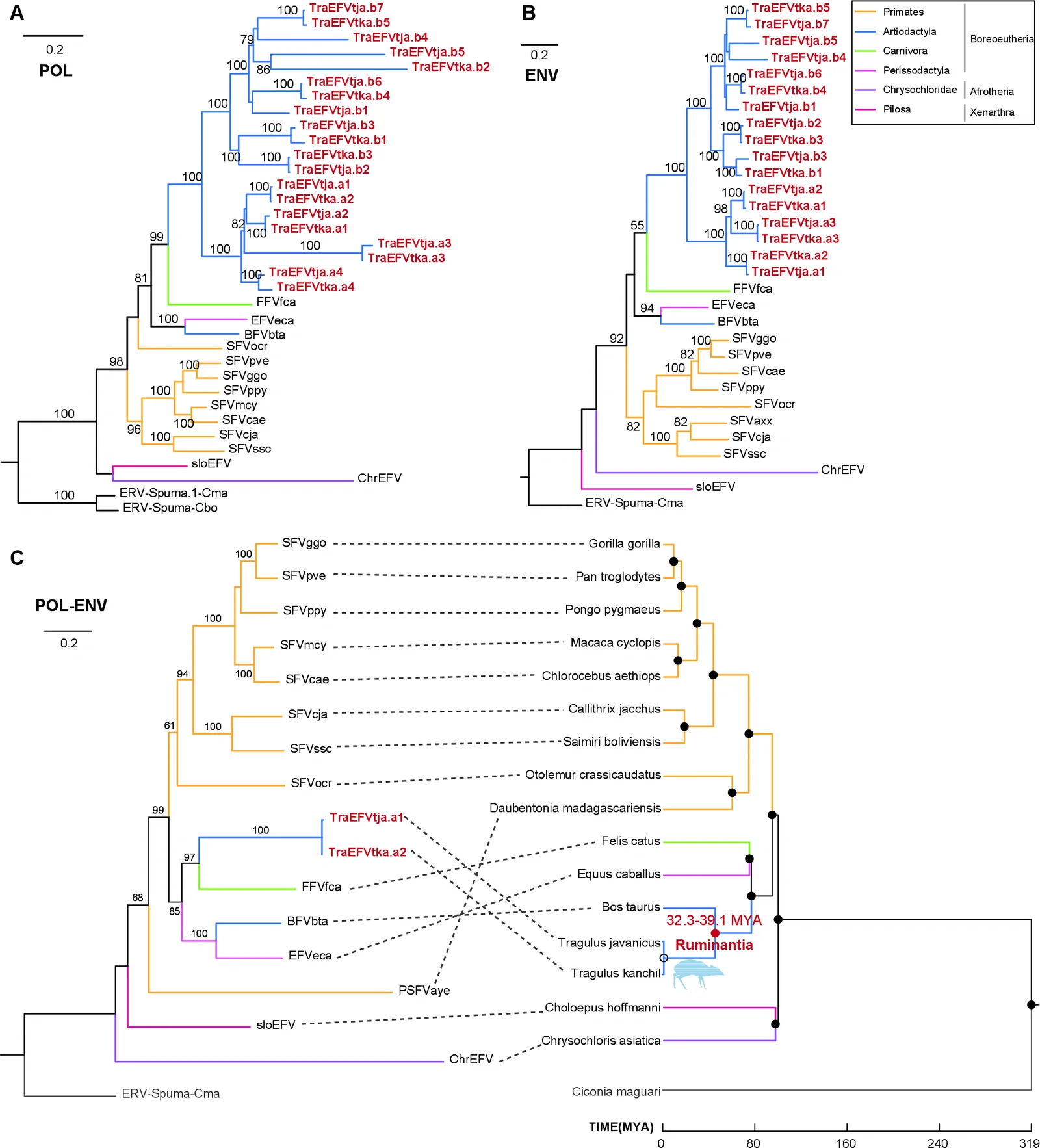
Phylogenetic trees of mammalian FVs and TraEFVs. The trees were inferred using conserved amino acid sequences of POL (**A**), ENV (**B**), and POL-ENV (**C**). These trees are rooted by avian foamy viruses. The phylogenetic tree of hosts was generated by Timetree (http://timetree.org/). The newly identified TraEFVs are labeled in red. The hosts of foamy viruses are labeled with different colors. The scale bar indicates the number of amino acid changes per site. Bootstrap values of <70% are not shown.

To investigate the relationship between TraEFVs and other known exogenous and endogenous mammalian FVs, we constructed the phylogenetic tree of polymerases. Surprisingly, TraEFV formed the sister lineage with the feline foamy virus with robust support rather than clustered with BFV isolated from cattle (Fig. 3A). Notably, each copy of TraEFVtka was clustered with one copy of TraEFVtja, respectively, which was consistent with the above description of the similarity of TraEFVs nucleic acids, indicating that TraEFVtka and TraEFVtja might have been vertically inherited. In addition, TraEFVs were classified into two clades based on POL phylogenetic topologies, suggesting that mouse-deer had been infected with multiple lineages of foamy viruses.

Considering the possibility of TraEFV recombination, phylogenetic analyses of the other major structural proteins (GAG, ENV) were performed (Fig. 3B; Fig. S1). Although the topologies of the GAG, POL, and ENV phylogenetic trees were inconsistent, TraEFVs stably clustered with feline FVs, suggesting that TraEFVs and feline FVs originated from a common ancestral virus. The inconsistency between TraEFVs and their hosts indicated that TraEFVs may be acquired from cross-species transmission (Fig. 3C).

### Ancient nature of TraEFV

**Fig 4:**
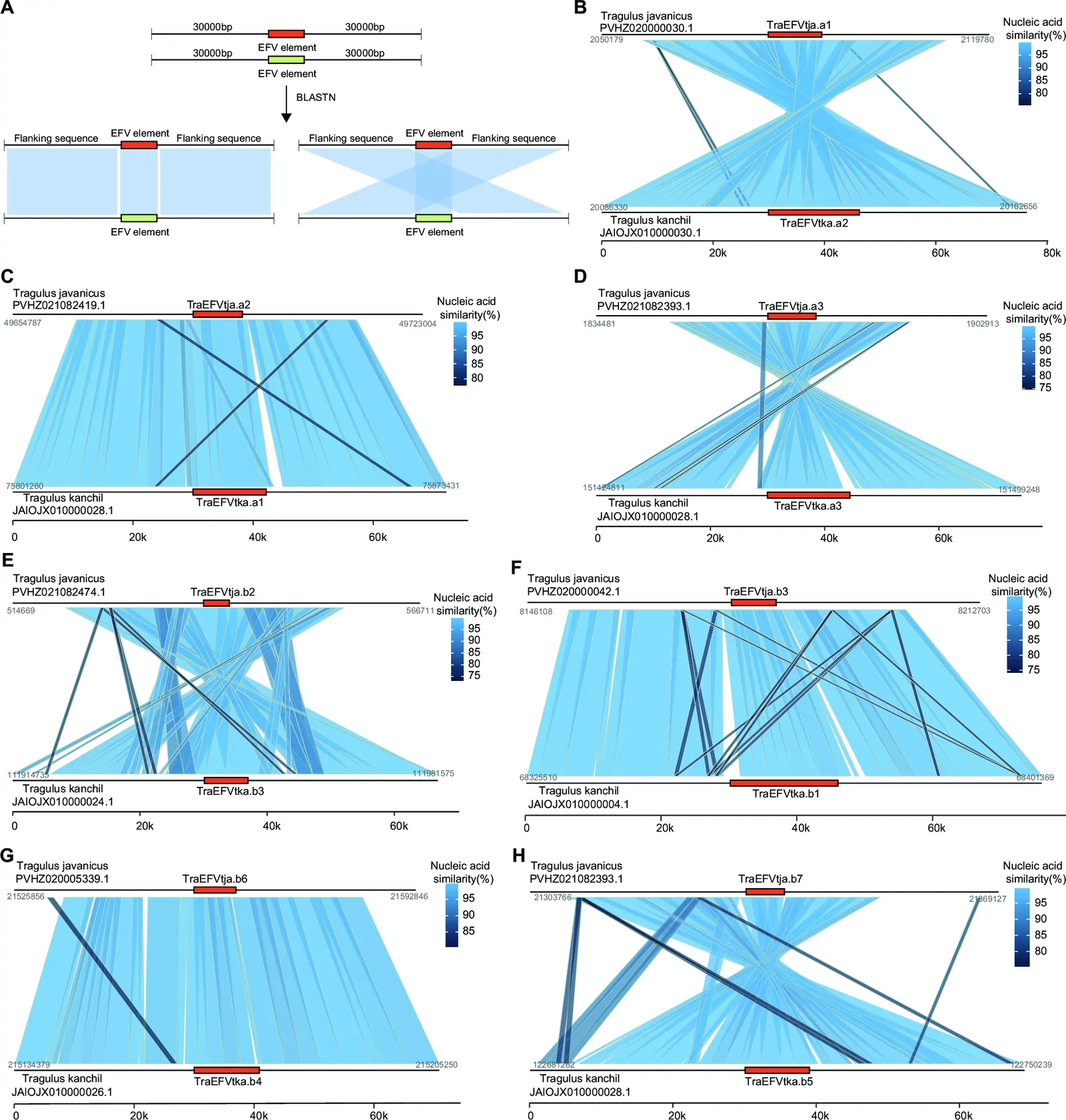
TraEFVs orthologous insertions in *T. javanicus* and *T. kanchil*. (**A**) Schematic representation of the identification of vertical transmission events. The flanking sequences of TraEFVs, with the length of 30,000 bp, were extracted. Then, the homologous regions of TraEFVs and their flanking sequences were detected by BLASTN. (**B–H**) The synteny maps of TraEFVs and their flanking sequences. The links indicate the homologous regions. TraEFVs were labeled with orange boxes.

Based on the relationship between each copy of TraEFVtja and TraEFVtka, we attempted to find molecular evidence of vertical transmission by comparing the similarity of flanking sequences surrounding TraEFVs in different species (Fig. 4A). As expected, seven orthologous insertion events of TraEFVtja and TraEFVtka were identified (Fig. 4B through H), indicating TraEFVtja and TraEFVtka had vertically inherited history. Therefore, it is likely that these foamy viruses integrated into the germ line of the common ancestor of *T. kanchil* and *T. javanicus*.

We also used the molecular dating method based on pairwise LTR divergence to estimate the integration time of TraEFVs. Only TraEFVtja.a had pairwise LTRs. And, it was found that the integration time of TraEFV.tja.a could be traced back to 20 million years ago (MYA), which is much older than the divergence time of *T. kanchil* and *T. javanicus* (about 2.8 MYA) (24). Significantly, Hyemoschus and Tragulus diverged about 23 MYA (24), close to the integration time of TraEFV.tja.a. Thus, it is likely that one lineage of TraEFVs invaded the mouse-deer genomes after the divergence of Hyemoschus and Tragulus during the Miocene. However, no LTRs could be detected in the copies of TraEFVtka.b and TraEFVtja.b, so the accurate insertion time of this lineage of TraEFVs cannot be estimated. To determine more accurate evolutionary history of TraEFVs, additional genomes from Tragulidae with virus integrated should be investigated in the future.

## DISCUSSION

In this study, a novel endogenous foamy virus was discovered in genomes of two species of mouse-deer and termed as TraEFV. TraEFVs exhibited two notable phenomena: (i) TraEFVs were acquired through cross-species transmission and (ii) TraEFVs showed vertical transmission in *T. kanchil* and *T. javanicus*. The finding of TraEFVs suggests that FVs may have a wider range of hosts beyond what we currently know. In fact, this is not the first study that identified cross-species transmission of FVs. Previously, one group found that the evolutionary history of EFV identified in aye-aye (12) conflicted with the virus-host co-divergence pattern of FVs. Furthermore, phylogenetic analysis of EFVs in coelacanth (19, 23) and Mexican spadefoot toad (20) demonstrated that FVs can spread among different species and even across land and water boundaries.

Multiple infiltrations of different lineages have also been reported such that we previously found two lineages of salamander EFVs were integrated into the host genome at different times (ERV-Spuma.a-Ame: 18.92–55.95 MYA, ERV-Spuma.b-Ame: 2.39–24.89 MYA) (20). In the case of TraEFV, we also observed two lineages—TraEFV.a and TraEFV.b may represent two independent viral infection events, although TraEFV.b lacks LTRs, making it impossible to estimate viral insertion time by LTR dating.

## MATERIALS AND METHODS

### Genome screening and identification of TraEFVs

To identify potential endogenous foamy viral elements, 524 reference mammalian genome assemblies (Table S1) were screened using the tBLASTn algorithm (25), and the POL protein sequences of all available foamy retroviruses (including endogenous forms) and foamy-like viruses were used as probes (Table S3). A 40% sequence identity over 40% of the region with an *e*-value of 1E−5 was used to filter significant hits. Next, these significant hits were confirmed by phylogeny, and those clustered with representative FVs were considered mammalian endogenous FVs (Table S2).

### Genomic annotation of TraEFVs

The flanking sequences of potential mammalian EFVs were extended to detect pairwise long-terminal repeats using LTR_finder (26) and LTR_harvest (27). The consensus sequences of TraEFVs could not be constructed due to the limited number of copies of EFVs and poorly aligned sequences. The distributions of ORFs in copies of EFV were determined using ORFfinder in the NCBI database and confirmed by BLASTp (25). However, the newly identified mammalian EFVs contained numerous pre-stop codon and frameshift mutations, and no completed ORFs were identified. Thus, the boundary of EFV coding genes was identified by using tBLASTn (25), and all coding genes of available mammalian foamy viruses were used as probes. The potential ORFs of EFVs were confirmed by searching against the nr database of NCBI. Conserved domains for each sequence were found using CD-Search against the CDD (https://www.ncbi.nlm.nih.gov/cdd/).

### Phylogenetic analysis

The protein sequences for RT, GAG, POL, ENV, and concatenated POL-ENV were aligned using MAFFT 7.222 (28). The poorly aligned regions in the alignment were removed using TrimAL (29) and manually confirmed with MEGA X (30). A sequence was excluded if its length was less than 50% of the alignment. The phylogenetic trees for these protein sequences were inferred using the maximum likelihood method in IQ-Tree by incorporating 100 bootstrap replicates for the assessment of node robustness (31). The best-fit models were estimated by the ModelFinder program of IQ-Tree (RT: LG + G4, POL: VT + F + G4, GAG: JTT + G4, ENV: JTT + F + G4, POL-ENV: LG + F + G4). The phylogenetic trees were viewed and annotated using FigTree V1.4.3 (https://github.com/rambaut/figtree/). The alignments performed in this study can be found in Data sets S1.

### Vertical transmission identification

To determine potential vertical transmission between TraEFVtja and TraEFVtka, we first extracted the flanking sequences surrounding TraEFVs with lengths of 30,000 bp. Then, we used dc-megablast with *e*-value of 1E−5 to detect the homologous regions of TraEFVs flanking sequences in different species. Pairwise comparison was performed between TraEFVtja and TraEFVtka to determine if they exhibited vertical inheritance based on two criteria: (i) The nucleic acid similarity between TraEFVtja and TraEFVtka was over 90% and (ii) the coverage of flanking sequences alignments was over 50%. To avoid repeated hits of short repeat sequences, we excluded alignments less than 500 bp. The BLASTN results were visualized with a beta version of gggenomes (https://github.com/thackl/gggenomes).

### Molecular dating of TraEFVs

The EFV integration time can be estimated using the relationship *T* = (*D*/*R*)/2, in which *T* is the integration time (million years, MYA), *D* is the nucleotide divergence between a set of pairwise LTRs, and *R* is the genomic substitution rate (nucleotide substitutions per site, per year). We used a previously estimated evolutionary substitution rate for ancestral ruminants (~1.5 × 10^−9^ nucleotide substitutions per site, per year) (32) as the R to calculate the integration time of TraEFVtja.a1. The divergence of LTRs of TraEFVtja.a1 was 0.06.

## Data Availability

All the data needed to support the conclusions detailed in the article is included in the article itself and the supplemental material.

## SUPPLEMENTAL MATERIAL

FIg. S1, Tables S1 to S4, Dataset S1 spectrum.02090-23-s0001.pdf Supplemental materials.

ASM does not own the copyrights to Supplemental Material that may be linked to, or accessed through, an article. The authors have granted ASM a non-exclusive, world-wide license to publish the Supplemental Material files. Please contact the corresponding author directly for reuse.
