# Supplementary material for: Discovery of an endogenous foamy virus in primitive ruminant chevrotains
Source: Microbiol Spectr. 2023 Aug 15;11(5):e02090-23. doi: 10.1128/spectrum.02090-23 (PMC10581153; doi:10.1128/spectrum.02090-23)
Supplement: FIg. S1, Tables S1 to S4, Dataset S1 — Supplemental materials. [file spectrum.02090-23-s0001.pdf]

# Supplementary Materials

This PDF file includes:

**Fig. S1.** Phylogenetic trees of mammalian FVs and TraEFVs.

**Table S1.** Information of mammalian genomes used for data mining.

**Table S2.** The endogenous foamy viral elements identified in mammals.

**Table S3.** Information about the representative retroviruses.

**Table S4.** The tBLASTn results of accessory genes of TraEFVs.

**Dataset S1.** The alignments used to build the phylogenetic trees for RT, POL, GAG, ENV and POL-ENV.

**Reference**

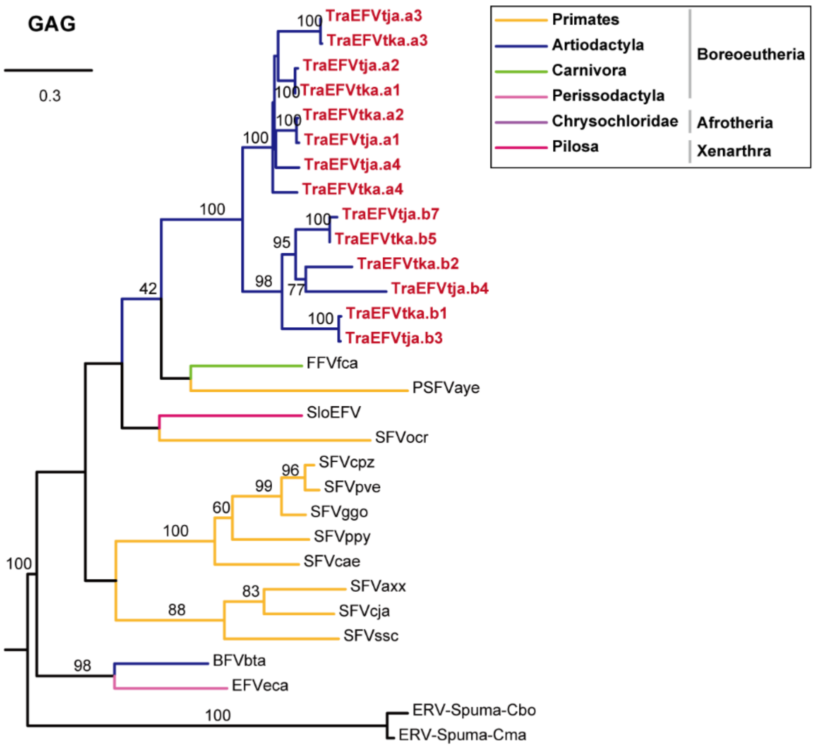


**Fig. S1.** Phylogenetic trees of mammalian FVs and TraEFVs. The tree was inferred using conserved amino acid sequences of GAG. These trees are rooted by avian foamy viruses (ERV-Spuma-Cbo and ERV-Spuma-Cma). The newly identified TraEFVs are labeled in red. The hosts of foamy viruses are labeled with different colors. The scale bar indicates the number of amino acid changes per site. Bootstrap values of <70% are not shown.

**Table S1. Information of mammalian genomes used for data mining.**

| Latin name | Assembly number |
|---|---|
| Acinonyx jubatus | GCA_003709585.1 |
| Acomys cahirinus | GCA_004027535.1 |
| Acomys dimidiatus | GCA_907164435.1 |
| Acomys kempi | GCA_907164505.1 |
| Acomys percivali | GCA_907169655.1 |
| Acomys russatus | GCA_903995435.1 |
| Addax nasomaculatus | GCA_019593525.1 |
| Aeorestes cinereus | GCA_011751065.1 |
| Aepyceros melampus | GCA_006408695.1 |
| Ailuropoda melanoleuca | GCA_002007445.3 |
| Ailurus styani | GCA_002007465.1 |
| Alcelaphus buselaphus | GCA_006408545.1 |
| Alces alces | GCA_007570765.1 |
| Alouatta palliata | GCA_004027835.1 |
| Ammotragus lervia | GCA_002201775.1 |
| Anoura caudifer | GCA_004027475.1 |
| Antechinus flavipes | GCA_016432865.2 |
| Antechinus stuartii | GCA_016696395.1 |
| Antidorcas marsupialis | GCA_006408585.1 |
| Antilocapra americana | GCA_007570785.1 |
| Antrozous pallidus | GCA_007922775.1 |
| Aotus nancymaae | GCA_000952055.2 |
| Aplodontia rufa | GCA_004027875.1 |
| Apodemus speciosus | GCA_002335545.1 |
| Apodemus sylvaticus | GCA_947179515.1 |
| Arctocephalus gazella | GCA_900642305.1 |
| Artibeus jamaicensis | GCA_014825515.1 |
| Arvicanthis niloticus | GCA_011762505.1 |
| Arvicola amphibius | GCA_903992535.2 |
| Ateles geoffroyi | GCA_023783555.1 |
| Ateles hybridus | GCA_916098195.1 |
| Axis porcinus | GCA_003798545.1 |
| Balaenoptera acutorostrata scammoni | GCA_000493695.1 |
| Balaenoptera bonaerensis | GCA_000978805.1 |
| Balaenoptera musculus | GCA_009873245.3 |
| Balaenoptera physalus | GCA_023338255.1 |
| Beatragus hunteri | GCA_004027495.1 |
| Bettongia penicillata ogilbyi | GCA_023548195.1 |
| Bison bison bison | GCA_000754665.1 |
| Bos frontalis | GCA_007844835.1 |
| Bos gaurus | GCA_014182915.2 |
| Bos gaurus x Bos taurus | GCA_946052875.1 |

| | |
|---|---|
| Bos grunniens | GCA_005887515.3 |
| Bos grunniens x Bos taurus | GCA_009493645.1 |
| Bos indicus | GCA_000247795.2 |
| Bos indicus x Bos taurus | GCA_003369695.2 |
| Bos mutus | GCA_000298355.1 |
| Bos taurus | GCA_002263795.3 |
| Bos taurus x Bison bison | GCA_018282465.1 |
| **Bradypus variegatus #** | **GCA_004027775.1** |
| Bubalus bubalis | GCA_019923935.1 |
| Bubalus depressicornis | GCA_025584935.1 |
| Budorcas taxicolor | GCA_023091745.1 |
| Callithrix jacchus | GCA_009663435.2 |
| Callorhinus ursinus | GCA_003265705.1 |
| Camelus bactrianus | GCA_000767855.1 |
| Camelus dromedarius | GCA_000803125.3 |
| Camelus ferus | GCA_009834535.1 |
| Canis lupus dingo | GCA_003254725.2 |
| Canis lupus familiaris | GCA_014441545.1 |
| Capra aegagrus | GCA_000978405.1 |
| Capra hircus | GCA_001704415.2 |
| Capra ibex | GCA_006410555.1 |
| Capra sibirica | GCA_003182615.2 |
| Capreolus capreolus | GCA_000751575.1 |
| Capreolus pygargus | GCA_012922965.1 |
| Capromys pilorides | GCA_004027915.1 |
| Caracal caracal | GCA_016801355.1 |
| Carlito syrichta | GCA_000164805.2 |
| Carollia perspicillata | GCA_004027735.1 |
| Castor canadensis | GCA_001984765.1 |
| Catagonus wagneri | GCA_004024745.2 |
| Cavia aperea | GCA_000688575.1 |
| Cavia porcellus | GCA_000151735.1 |
| Cavia tschudii | GCA_004027695.1 |
| Cebus albifrons | GCA_023783575.1 |
| Cebus imitator | GCA_001604975.1 |
| Cephalophus harveyi | GCA_006410635.1 |
| Ceratotherium simum simum | GCA_000283155.1 |
| Cercocebus atys | GCA_000955945.1 |
| Cercopithecus albogularis | GCA_023783535.1 |
| Cercopithecus mona | GCA_014849445.1 |
| Cercopithecus neglectus | GCA_004027615.1 |
| Cervus albirostris | GCA_006408465.1 |
| Cervus canadensis | GCA_019320065.1 |
| Cervus elaphus | GCA_910594005.1 |

| | |
|---|---|
| Cervus hanglu yarkandensis | GCA_010411085.1 |
| Chaetophractus vellerosus | GCA_004027955.1 |
| Cheirogaleus medius | GCA_008086735.1 |
| Chinchilla lanigera | GCA_000276665.1 |
| Chlorocebus aethiops | GCA_023783515.1 |
| Chlorocebus sabaeus | GCA_015252025.1 |
| **Choloepus didactylus #** | **GCA_015220235.1** |
| **Choloepus hoffmanni #** | **GCA_000164785.2** |
| **Chrysochloris asiatica #** | **GCA_000296735.1** |
| Chrysocyon brachyurus | GCA_024262425.1 |
| Colobus angolensis palliatus | GCA_000951035.1 |
| Colobus guereza | GCA_021498455.1 |
| Condylura cristata | GCA_000260355.1 |
| Connochaetes taurinus | GCA_006408615.1 |
| Craseonycteris thonglongyai | GCA_004027555.1 |
| Cricetomys gambianus | GCA_004027575.1 |
| Cricetulus griseus | GCA_000223135.1 |
| Crocidura indochinensis | GCA_004027635.1 |
| Crocuta crocuta | GCA_008692635.1 |
| Cryptoprocta ferox | GCA_004023885.1 |
| Cryptotis parvus | GCA_021461705.1 |
| Ctenodactylus gundi | GCA_004027205.1 |
| Ctenomys sociabilis | GCA_004027165.1 |
| Cuniculus paca | GCA_004365215.1 |
| Cynomys gunnisoni | GCA_011316645.1 |
| Cynopterus brachyotis | GCA_009793145.1 |
| Damaliscus lunatus | GCA_006408505.1 |
| Dasyprocta punctata | GCA_004363535.1 |
| Dasypus novemcinctus | GCA_000208655.4 |
| Dasyurus viverrinus | GCA_020854095.1 |
| **Daubentonia madagascariensis #** | **GCA_023783475.1** |
| Delphinapterus leucas | GCA_002288925.3 |
| Desmodus rotundus | GCA_002940915.3 |
| Dicerorhinus sumatrensis harrissoni | GCA_014189135.1 |
| Diceros bicornis minor | GCA_020826845.1 |
| Dinomys branickii | GCA_004027595.1 |
| Dipodomys merriami | GCA_024711535.1 |
| Dipodomys ordii | GCA_000151885.2 |
| Dipodomys spectabilis | GCA_019054845.1 |
| Dipodomys stephensi | GCA_004024685.1 |
| Dolichotis patagonum | GCA_004027295.1 |
| Dromiciops gliroides | GCA_019393635.1 |
| Dugong dugon | GCA_905400935.1 |
| Echinops telfairi | GCA_000313985.2 |

| | |
|---|---|
| Eidolon helvum | GCA_000465285.1 |
| Eira barbara | GCA_020311275.1 |
| Elaphurus davidianus | GCA_021018665.1 |
| Elephantulus edwardii | GCA_000299155.1 |
| Elephas maximus indicus | GCA_024166365.1 |
| Ellobius lutescens | GCA_001685075.1 |
| Ellobius talpinus | GCA_001685095.1 |
| Enhydra lutris kenyoni | GCA_002288905.2 |
| Eonycteris spelaea | GCA_003508835.1 |
| Eptesicus fuscus | GCA_000308155.1 |
| Equus asinus | GCA_016077325.2 |
| Equus caballus | GCA_002863925.1 |
| Equus przewalskii | GCA_000696695.1 |
| Equus quagga | GCA_021613505.1 |
| Erethizon dorsatum | GCA_006547115.1 |
| Erinaceus europaeus | GCA_000296755.1 |
| Erythrocebus patas | GCA_023783455.1 |
| Eschrichtius robustus | GCA_002189225.1 |
| Eubalaena japonica | GCA_004363455.1 |
| Eudorcas thomsonii | GCA_006408755.1 |
| Eulemur flavifrons | GCA_001262665.1 |
| Eulemur fulvus | GCA_004027275.1 |
| Eulemur macaco | GCA_001262655.1 |
| Eumetopias jubatus | GCA_004028035.1 |
| Felis catus | GCA_018350175.1 |
| Felis chaus | GCA_019924945.1 |
| Felis nigripes | GCA_004023925.1 |
| Fukomys damarensis | GCA_012274545.1 |
| Galago moholi | GCA_023783435.1 |
| Galemys pyrenaicus | GCA_019455555.1 |
| Galeopterus variegatus | GCA_000696425.1 |
| Giraffa camelopardalis rothschildi | GCA_017591445.1 |
| Giraffa tippelskirchi | GCA_013496395.1 |
| Glaucomys volans | GCA_020662805.1 |
| Glis glis | GCA_004027185.1 |
| Globicephala melas | GCA_006547405.1 |
| Gorilla gorilla gorilla | GCA_008122165.1 |
| Gracilinanus agilis | GCA_016433145.1 |
| Grammomys dolichurus | GCA_019843835.1 |
| Grammomys surdaster | GCA_004785775.1 |
| Graphiurus murinus | GCA_004027655.1 |
| Gulo gulo luscus | GCA_024510155.1 |
| Gymnobelideus leadbeateri | GCA_011680675.1 |
| Halichoerus grypus | GCA_012393455.1 |

| | |
|---|---|
| Helogale parvula | GCA_004023845.1 |
| Hemitragus hylocrius | GCA_004026825.1 |
| Heterocephalus glaber | GCA_000247695.1 |
| Heterohyrax brucei | GCA_004026845.1 |
| Hexaprotodon liberiensis | GCA_023065765.1 |
| Hippopotamus amphibius | GCA_023065835.1 |
| Hipposideros armiger | GCA_001890085.1 |
| Hipposideros galeritus | GCA_004027415.1 |
| Hipposideros pendleburyi | GCA_021464545.1 |
| Hippotragus equinus | GCA_016433095.1 |
| Hippotragus niger niger | GCA_006942125.1 |
| Homo sapiens | GCA_000001405.29 |
| Hoolock leuconedys | GCA_023748175.1 |
| Hyaena hyaena | GCA_003009895.1 |
| Hydrochoerus hydrochaeris | GCA_004027455.1 |
| Hydrodamalis gigas | GCA_013391785.1 |
| Hydropotes inermis | GCA_020226075.1 |
| Hylobates moloch | GCA_009828535.3 |
| Hylobates pileatus | GCA_021498465.1 |
| Hylomyscus alleni | GCA_019843855.1 |
| Hyperoodon ampullatus | GCA_024363105.1 |
| Hystrix brachyura | GCA_016801275.1 |
| Hystrix cristata | GCA_004026905.1 |
| Ia io | GCA_025583905.1 |
| Ictidomys tridecemlineatus | GCA_016881025.1 |
| Indri indri | GCA_004363605.1 |
| Inia geoffrensis | GCA_004363515.1 |
| Jaculus jaculus | GCA_020740685.1 |
| Kobus ellipsiprymnus | GCA_006410655.1 |
| Kobus leche leche | GCA_014926565.1 |
| Kogia breviceps | GCA_004363705.1 |
| Lagenorhynchus obliquidens | GCA_003676395.1 |
| Lama glama chaku | GCA_013239585.1 |
| Lama guanicoe cacsilensis | GCA_013239625.1 |
| Lasiurus borealis | GCA_004026805.1 |
| Lemur catta | GCA_020740605.1 |
| Leopardus geoffroyi | GCA_018350155.1 |
| Leptonychotes weddellii | GCA_000349705.1 |
| Lepus americanus | GCA_004026855.1 |
| Lepus timidus | GCA_009760805.1 |
| Lipotes vexillifer | GCA_000442215.1 |
| Litocranius walleri | GCA_006410535.1 |
| Lontra canadensis | GCA_010015895.1 |
| Lophiomys imhausi | GCA_907164525.1 |

| | |
|---|---|
| Lophocebus aterrimus | GCA_023783235.1 |
| Loris tardigradus | GCA_023783135.1 |
| Loxodonta africana | GCA_000001905.1 |
| Lutra lutra | GCA_902655055.2 |
| Lycaon pictus | GCA_004216515.1 |
| Lynx canadensis | GCA_007474595.2 |
| Lynx pardinus | GCA_900661375.1 |
| Lynx rufus | GCA_022079265.1 |
| Macaca arctoides | GCA_021188215.1 |
| Macaca assamensis | GCA_023783095.1 |
| Macaca fascicularis | GCA_012559485.3 |
| Macaca fuscata fuscata | GCA_003118495.1 |
| Macaca mulatta | GCA_003339765.3 |
| Macaca nemestrina | GCA_000956065.1 |
| Macaca nigra | GCA_928851695.1 |
| Macaca silenus | GCA_023807365.1 |
| Macaca thibetana thibetana | GCA_024542745.1 |
| Macroglossus sobrinus | GCA_004027375.1 |
| Macrotus californicus | GCA_007922815.1 |
| Madoqua kirkii | GCA_006408675.1 |
| Mandrillus leucophaeus | GCA_000951045.1 |
| Mandrillus sphinx | GCA_023783085.1 |
| Manis crassicaudata | GCA_016801295.1 |
| Manis javanica | GCA_014570535.1 |
| Manis pentadactyla | GCA_014570555.1 |
| Marmota flaviventris | GCA_003676075.3 |
| Marmota himalayana | GCA_005280165.1 |
| Marmota marmota marmota | GCA_001458135.2 |
| Marmota monax | GCA_021218885.2 |
| Marmota vancouverensis | GCA_005458795.1 |
| Martes zibellina | GCA_012583365.1 |
| Mastomys coucha | GCA_008632895.1 |
| Mastomys natalensis | GCA_021653895.1 |
| Megaderma lyra | GCA_004026885.1 |
| Megaptera novaeangliae | GCA_004329385.1 |
| Meles meles | GCA_922984935.2 |
| Mellivora capensis | GCA_004024625.1 |
| Meriones unguiculatus | GCA_002204375.1 |
| Mesocricetus auratus | GCA_017639785.1 |
| Mesoplodon bidens | GCA_004027085.1 |
| Mesoplodon densirostris | GCA_025265405.1 |
| Microcebus griseorufus | GCA_008750995.1 |
| Microcebus mittermeieri | GCA_008750955.1 |
| Microcebus murinus | GCA_000165445.3 |

| | |
|---|---|
| Microcebus ravelobensis | GCA_008750975.1 |
| Microcebus sp. 3 GT-2019 | GCA_008750915.1 |
| Microcebus tavaratra | GCA_008750935.1 |
| Microgale talazaci | GCA_004026705.1 |
| Micronycteris hirsuta | GCA_004026765.1 |
| Microtus agrestis | GCA_902806775.1 |
| Microtus arvalis | GCA_007455615.1 |
| Microtus fortis | GCA_014885135.2 |
| Microtus montanus | GCA_020392405.1 |
| Microtus ochrogaster | GCA_000317375.1 |
| Microtus oeconomus | GCA_007455595.1 |
| Microtus oregoni | GCA_018167655.1 |
| Microtus richardsoni arvicoloides | GCA_020387435.1 |
| Miniopterus natalensis | GCA_001595765.1 |
| Miniopterus schreibersii | GCA_004026525.1 |
| Mirounga angustirostris | GCA_021288785.2 |
| Mirounga leonina | GCA_011800145.1 |
| Mirza coquereli | GCA_004024645.1 |
| Mirza zaza | GCA_008750895.1 |
| Molossus molossus | GCA_014108415.1 |
| Monodelphis domestica | GCA_000002295.1 |
| Monodon monoceros | GCA_005190385.3 |
| Mormoops blainvillei | GCA_004026545.1 |
| Moschus berezovskii | GCA_022376915.1 |
| Moschus chrysogaster | GCA_006461725.1 |
| Moschus moschiferus | GCA_004024705.2 |
| Mungos mungo | GCA_004023785.1 |
| Muntiacus crinifrons | GCA_020276665.1 |
| Muntiacus gongshanensis | GCA_020226025.1 |
| Muntiacus muntjak | GCA_008782695.1 |
| Muntiacus reevesi | GCA_020226045.1 |
| Murina aurata feae | GCA_004026665.1 |
| Mus caroli | GCA_900094665.2 |
| Mus minutoides | GCA_902729485.2 |
| Mus musculus | GCA_000001635.9 |
| Mus pahari | GCA_900095145.2 |
| Mus spicilegus | GCA_003336285.1 |
| Mus spretus | GCA_921997135.2 |
| Muscardinus avellanarius | GCA_004027005.1 |
| Mustela erminea | GCA_009829155.1 |
| Mustela nigripes | GCA_022355385.1 |
| Mustela nivalis | GCA_019141155.1 |
| Mustela putorius furo | GCA_011764305.2 |
| Myocastor coypus | GCA_004027025.1 |

| | |
|---|---|
| Myodes glareolus | GCA_902806735.1 |
| Myotis brandtii | GCA_000412655.1 |
| Myotis davidii | GCA_000327345.1 |
| Myotis lucifugus | GCA_000147115.1 |
| Myotis myotis | GCA_014108235.1 |
| Myrmecobius fasciatus | GCA_023553655.1 |
| Myrmecophaga tridactyla | GCA_004026745.1 |
| Nanger dama | GCA_917880005.1 |
| Nanger granti | GCA_006408635.1 |
| Nannospalax galili | GCA_000622305.1 |
| Nasalis larvatus | GCA_000772465.1 |
| Neodon shergylaensis | GCA_025134845.1 |
| Neogale vison | GCA_020171115.1 |
| Neomonachus schauinslandi | GCA_002201575.2 |
| Neophocaena asiaeorientalis asiaeorientalis | GCA_020395145.1 |
| Neotoma lepida | GCA_001675575.1 |
| Neotragus moschatus | GCA_006410615.1 |
| Neotragus pygmaeus | GCA_006410875.1 |
| Noctilio leporinus | GCA_004026585.1 |
| Nomascus leucogenys | GCA_006542625.1 |
| Nomascus siki | GCA_023783065.1 |
| Notamacropus eugenii | GCA_000004035.1 |
| Nyctereutes procyonoides | GCA_905146905.1 |
| Nycticebus bengalensis | GCA_023898255.1 |
| Nycticebus coucang | GCA_004027815.1 |
| Nycticeius humeralis | GCA_007922795.1 |
| Ochotona curzoniae | GCA_017591425.1 |
| Ochotona princeps | GCA_014633375.1 |
| Octodon degus | GCA_000260255.1 |
| Octomys mimax | GCA_002564305.1 |
| Odobenus rosmarus divergens | GCA_000321225.1 |
| Odocoileus hemionus | GCA_020976825.1 |
| Odocoileus virginianus texanus | GCA_002102435.1 |
| Okapia johnstoni | GCA_024291935.2 |
| Ondatra zibethicus | GCA_004026605.1 |
| Onychomys torridus | GCA_903995425.1 |
| Orcinus orca | GCA_937001465.1 |
| Oreamnos americanus | GCA_009758055.1 |
| Oreotragus oreotragus | GCA_006410675.1 |
| Orientallactaga bullata | GCA_004027895.1 |
| Ornithorhynchus anatinus | GCA_004115215.4 |
| Orycteropus afer afer | GCA_000298275.1 |
| Oryctolagus cuniculus | GCA_009806435.2 |

| | |
|---|---|
| Oryx dammah | GCA_014754425.2 |
| Oryx gazella | GCA_003945745.1 |
| Otocyon megalotis megalotis | GCA_017311455.1 |
| Otolemur garnettii | GCA_000181295.3 |
| Ourebia ourebi | GCA_006417275.1 |
| Ovibos moschatus | GCA_021462335.1 |
| Ovis ammon | GCA_003121645.1 |
| Ovis ammon polii x Ovis aries | GCA_023701675.1 |
| Ovis aries | GCA_016772045.1 |
| Ovis canadensis | GCA_004026945.1 |
| Ovis nivicola lydekkeri | GCA_903231385.1 |
| Ovis orientalis | GCA_014523465.1 |
| Pan paniscus | GCA_013052645.3 |
| Pan troglodytes | GCA_002880755.3 |
| Panthera leo | GCA_018350215.1 |
| Panthera onca | GCA_004023805.1 |
| Panthera pardus | GCA_001857705.1 |
| Panthera tigris | GCA_018350195.2 |
| Panthera uncia | GCA_023721935.1 |
| Papio anubis | GCA_008728515.2 |
| Papio hamadryas | GCA_023781915.1 |
| Paradoxurus hermaphroditus | GCA_004024585.1 |
| Pedetes capensis | GCA_007922755.1 |
| Perognathus longimembris pacificus | GCA_023159225.1 |
| Peromyscus attwateri | GCA_902168425.1 |
| Peromyscus aztecus | GCA_902168405.1 |
| Peromyscus californicus insignis | GCA_007827085.3 |
| Peromyscus eremicus | GCA_902702925.1 |
| Peromyscus leucopus | GCA_004664715.2 |
| Peromyscus maniculatus bairdii | GCA_003704035.3 |
| Peromyscus melanophrys | GCA_902168415.1 |
| Peromyscus nudipes | GCA_902168325.1 |
| Peromyscus polionotus subgriseus | GCA_003704135.2 |
| Petromus typicus | GCA_004026965.1 |
| Phacochoerus africanus | GCA_016906955.1 |
| Phascolarctos cinereus | GCA_002099425.1 |
| Phataginus tricuspis | GCA_004765945.2 |
| Philantomba maxwellii | GCA_006410695.1 |
| Phoca vitulina | GCA_004348235.1 |
| Phocoena phocoena | GCA_004363495.1 |
| Phocoena sinus | GCA_008692025.1 |
| Phodopus roborovskii | GCA_943737965.1 |
| Phodopus sungorus | GCA_023856395.1 |
| Phyllostomus discolor | GCA_004126475.3 |

| | |
|---|---|
| Phyllostomus hastatus | GCA_019186645.2 |
| Physeter catodon | GCA_002837175.2 |
| Piliocolobus tephrosceles | GCA_002776525.4 |
| Pipistrellus kuhlii | GCA_014108245.1 |
| Pipistrellus pipistrellus | GCA_903992545.1 |
| Pithecia pithecia | GCA_023779675.1 |
| Platanista gangetica | GCA_017311385.1 |
| Platanista minor | GCA_004363435.1 |
| Plecturocebus donacophilus | GCA_004027715.1 |
| Pongo abelii | GCA_002880775.3 |
| Pongo pygmaeus | GCA_947095605.1 |
| Pontoporia blainvillei | GCA_011754075.1 |
| Potos flavus | GCA_015708855.1 |
| Praomys delectorum | GCA_019843815.1 |
| Prionailurus bengalensis | GCA_016509475.2 |
| Prionailurus iriomotensis | GCA_018403415.1 |
| Prionailurus viverrinus | GCA_022837055.1 |
| Procapra przewalskii | GCA_006410515.1 |
| Procavia capensis | GCA_004026925.3 |
| Procyon lotor | GCA_015708975.1 |
| Prolemur simus | GCA_003258685.1 |
| Propithecus coquereli | GCA_000956105.1 |
| Proteles cristata cristata | GCA_017311185.1 |
| Psammomys obesus | GCA_907164565.1 |
| Pseudois nayaur | GCA_003182575.1 |
| Pteronotus parnellii mesoamericanus | GCA_021234165.1 |
| Pteronura brasiliensis | GCA_004024605.1 |
| Pteropus alecto | GCA_000325575.1 |
| Pteropus giganteus | GCA_902729225.1 |
| Pteropus pselaphon | GCA_014363405.1 |
| Pteropus vampyrus | GCA_000151845.2 |
| Puma concolor | GCA_003327715.1 |
| Puma yagouaroundi | GCA_014898765.1 |
| Pygathrix nemaeus | GCA_004024825.1 |
| Pygathrix nigripes | GCA_023764695.1 |
| Rangifer tarandus caribou | GCA_019903745.1 |
| Raphicerus campestris | GCA_006410735.1 |
| Rattus norvegicus | GCA_015227675.2 |
| Rattus rattus | GCA_011064425.1 |
| Redunca redunca | GCA_006410935.1 |
| Rhabdomys dilectus | GCA_019844195.1 |
| Rhinoceros unicornis | GCA_019022865.1 |
| Rhinolophus ferrumequinum | GCA_004115265.3 |
| Rhinopithecus bieti | GCA_001698545.2 |

| | |
|---|---|
| Rhinopithecus roxellana | GCA_007565055.1 |
| Rhinopithecus strykeri | GCA_023764705.1 |
| Rhizomys pruinosus | GCA_009823505.1 |
| Rhombomys opimus | GCA_010120015.1 |
| Rhynchomys soricoides | GCA_019843965.1 |
| Rousettus aegyptiacus | GCA_014176215.1 |
| Rousettus leschenaultii | GCA_015472975.1 |
| Saguinus imperator | GCA_004024885.1 |
| Saguinus midas | GCA_021498475.1 |
| Saiga tatarica | GCA_004024985.1 |
| Saimiri boliviensis boliviensis | GCA_016699345.2 |
| Sapajus apella | GCA_009761245.1 |
| Sarcophilus harrisii | GCA_902635505.1 |
| Scalopus aquaticus | GCA_004024925.1 |
| Sciurus carolinensis | GCA_902686445.2 |
| Sciurus niger | GCA_020740815.1 |
| Sciurus vulgaris | GCA_902686455.2 |
| Semnopithecus entellus | GCA_004025065.1 |
| Sigmodon hispidus | GCA_004025045.1 |
| Solenodon paradoxus | GCA_004363575.1 |
| Sorex araneus | GCA_000181275.2 |
| Sorex cinereus | GCA_026122425.1 |
| Sousa chinensis | GCA_007760645.1 |
| Speothos venaticus | GCA_023170115.1 |
| Spermophilus dauricus | GCA_002406435.1 |
| Spilogale gracilis | GCA_004023965.1 |
| Spilogale interrupta | GCA_023159085.1 |
| Sturnira hondurensis | GCA_014824575.2 |
| Suncus etruscus | GCA_024139225.1 |
| Suricata suricatta | GCA_006229205.1 |
| Sus cebifrons | GCA_905335845.1 |
| Sus scrofa | GCA_000003025.6 |
| Sylvicapra grimmia | GCA_006408735.1 |
| Sylvilagus bachmani | GCA_015711505.1 |
| Symphalangus syndactylus | GCA_023761135.1 |
| Syncerus caffer | GCA_902825105.1 |
| Tachyglossus aculeatus | GCA_015852505.1 |
| Tadarida brasiliensis | GCA_004025005.1 |
| Talpa occidentalis | GCA_014898055.1 |
| Tamandua tetradactyla | GCA_023851605.1 |
| Tamias sibiricus | GCA_025594165.1 |
| Tapirus indicus | GCA_004024905.1 |
| Tapirus terrestris | GCA_004025025.1 |
| Taxidea taxus jeffersonii | GCA_003697995.1 |

| | |
|---|---|
| Theropithecus gelada | GCA_003255815.1 |
| Thomomys bottae | GCA_024803745.1 |
| Thryonomys swinderianus | GCA_004025085.1 |
| Thylacinus cynocephalus | GCA_007646695.3 |
| Tolypeutes matacus | GCA_004025125.1 |
| Tonatia saurophila | GCA_004024845.1 |
| Trachypithecus francoisi | GCA_009764315.1 |
| Trachypithecus phayrei crepuscula | GCA_023762245.1 |
| Tragelaphus buxtoni | GCA_006411685.1 |
| Tragelaphus eurycerus | GCA_935064755.1 |
| Tragelaphus imberbis | GCA_006410775.1 |
| Tragelaphus oryx | GCA_006416875.1 |
| Tragelaphus scriptus | GCA_006410495.1 |
| Tragelaphus spekii | GCA_006411015.1 |
| Tragelaphus strepsiceros | GCA_006410795.1 |
| **Tragulus javanicus #** | **GCA_004024965.2** |
| **Tragulus kanchil #** | **GCA_022376925.1** |
| Tremarctos ornatus | GCA_018398825.1 |
| Trichechus manatus latirostris | GCA_000243295.1 |
| Trichosurus vulpecula | GCA_011100635.1 |
| Tupaia belangeri | GCA_000181375.1 |
| Tupaia chinensis | GCA_000334495.1 |
| Tupaia tana | GCA_026018925.1 |
| Tursiops aduncus | GCA_003227395.1 |
| Tursiops truncatus | GCA_011762595.1 |
| Tympanoctomys barrerae | GCA_002564285.1 |
| Typhlomys cinereus | GCA_023101885.1 |
| Urocitellus parryii | GCA_003426925.1 |
| Uropsilus gracilis | GCA_004024945.1 |
| Ursus americanus | GCA_020975775.1 |
| Ursus arctos | GCA_023065955.1 |
| Ursus maritimus | GCA_017311325.1 |
| Ursus thibetanus thibetanus | GCA_009660055.1 |
| Vicugna pacos | GCA_000164845.5 |
| Vicugna vicugna mensalis | GCA_013265495.1 |
| Vombatus ursinus | GCA_900497805.2 |
| Vulpes ferrilata | GCA_024500485.1 |
| Vulpes lagopus | GCA_018345385.1 |
| Vulpes vulpes | GCA_003160815.1 |
| Xerus inauris | GCA_004024805.1 |
| Zalophus californianus | GCA_009762305.2 |
| Zapus hudsonius | GCA_004024765.1 |
| Ziphius cavirostris | GCA_004364475.1 |

# indicated species harbored endogenous foamy viral elements.

**Table S2. The endogenous foamy viral elements identified in mammals.**

| Accession number | EFVs name | Species | Start | End | Feature |
|---|---|---|---|---|---|
| PVHZ020000030.1 | TraEFVtja.a1 | Tragulus javanicus | 2080179 | 2089780 | LTR-gag-pol-env-acc-LTR |
| PVHZ021082419.1 | TraEFVtja.a2 | Tragulus javanicus | 49693004 | 49684787 | gag-pol-env |
| PVHZ021082393.1 | TraEFVtja.a3 | Tragulus javanicus | 1864481 | 1872913 | gag-pol-env-acc |
| PVHZ020000133.1 | TraEFVtja.a4 | Tragulus javanicus | 8973 | 2730 | gag-pol-env |
| PVHZ021082439.1 | TraEFVtja.b1 | Tragulus javanicus | 33921797 | 33926826 | pol-env |
| PVHZ021082474.1 | TraEFVtja.b2 | Tragulus javanicus | 544669 | 548719 | pol-env |
| PVHZ020000042.1 | TraEFVtja.b3 | Tragulus javanicus | 8176108 | 8182703 | pol-env |
| PVHZ020003498.1 | TraEFVtja.b4 | Tragulus javanicus | 167781 | 174307 | gag-pol-env |
| PVHZ020003498.1 | TraEFVtja.b5 | Tragulus javanicus | 250668 | 254435 | pol-env |
| PVHZ020005339.1 | TraEFVtja.b6 | Tragulus javanicus | 21555856 | 21562846 | pol-env |
| PVHZ021082393.1 | TraEFVtja.b7 | Tragulus javanicus | 21333766 | 21339127 | pol-env |
| JAIOJX010000028.1 | TraEFVtka.a1 | Tragulus kanchil | 75843431 | 75831260 | gag-pol-env-acc |
| JAIOJX010000030.1 | TraEFVtka.a2 | Tragulus kanchil | 20132656 | 20116330 | gag-pol-env-acc |
| JAIOJX010000028.1 | TraEFVtka.a3 | Tragulus kanchil | 151454811 | 151469248 | gag-pol-env |
| JAIOJX010000003.1 | TraEFVtka.a4 | Tragulus kanchil | 76534394 | 76528878 | gag-pol |
| JAIOJX010000004.1 | TraEFVtka.b1 | Tragulus kanchil | 68355510 | 68371369 | gag-pol-env |
| JAIOJX010000018.1 | TraEFVtka.b2 | Tragulus kanchil | 1077546 | 1084980 | gag-pol-env |
| JAIOJX010000024.1 | TraEFVtka.b3 | Tragulus kanchil | 111944735 | 111951575 | pol-env |
| JAIOJX010000026.1 | TraEFVtka.b4 | Tragulus kanchil | 215164379 | 215175250 | pol-env |
| JAIOJX010000028.1 | TraEFVtka.b5 | Tragulus kanchil | 122711262 | 122720239 | gag-pol-env |
| PVKY010023683.1 | - | Bradypus variegatus | 529 | 1962 | pol |
| PVKY010113869.1 | - | Bradypus variegatus | 595 | 1899 | pol |
| CM026693.1 | - | Choloepus didactylus | 76514523 | 76515773 | pol |
| CM026695.1 | - | Choloepus didactylus | 229487 | 230947 | pol |

| | | | | | |
|---|---|---|---|---|---|
| CM026696.1 | - | Choloepus didactylus | 101263471 | 101265162 | pol |
| CM026697.1 | - | Choloepus didactylus | 125138538 | 125139734 | pol |
| CM026699.1 | - | Choloepus didactylus | 12015560 | 12017761 | pol |
| CM026715.1 | - | Choloepus didactylus | 804464 | 806164 | pol |
| JADCNF010000019.1 | - | Choloepus didactylus | 1701634 | 1702926 | pol |
| JADCNF010000067.1 | - | Choloepus didactylus | 3525128 | 3526672 | pol |
| ABVD02267833.1 | - | Choloepus hoffmanni | 22517 | 24262 | pol |
| KN182126.1 | - | Choloepus hoffmanni | 131979 | 133412 | pol |
| KN186275.1 | - | Choloepus hoffmanni | 37955 | 39391 | pol |
| KN187399.1 | - | Choloepus hoffmanni | 185929 | 187725 | pol |
| KN190438.1 | - | Choloepus hoffmanni | 256975 | 258507 | pol |
| KN191700.1 | - | Choloepus hoffmanni | 87042 | 89243 | pol |
| KN194725.1 | - | Choloepus hoffmanni | 890524 | 892059 | pol |
| KN194761.1 | - | Choloepus hoffmanni | 206763 | 208166 | pol |
| KN194973.1 | - | Choloepus hoffmanni | 437570 | 440347 | pol |
| KN195535.1 | - | Choloepus hoffmanni | 203880 | 205136 | pol |
| KN195827.1 | - | Choloepus hoffmanni | 483982 | 486300 | pol |
| JH823331.1 | - | Chrysochloris asiatica | 7746789 | 7748123 | pol |
| JAKFHU010001865.1 | - | Daubentonia madagascariensis | 22517 | 24262 | pol |

**Table S3. Information about the representative retroviruses**

| Virus name | Genus | Abbreviation | Natural host | Accession no. |
|---|---|---|---|---|
| Avian leukemia virus | Alpharetrovirus | ALV | Chicken | NC_015116 |
| Lymphoproliferative disease virus | Alpharetrovirus | LDV | Turkey | U09568 |
| Mouse mammary tumor virus | Betaretrovirus | MMTV | Mouse | NC_001503 |
| Mason-Pfizer monkey virus | Betaretrovirus | MPMV | Primate | NC_001550 |
| Simian retrovirus 1 | Betaretrovirus | SRV1 | Primate | M11841 |
| Bovine leukemia virus | Deltaretrovirus | BLV | Cattle | NC_001414 |
| Human T-lymphotropic virus 1 | Deltaretrovirus | HTLV1 | Human | NC_001436 |
| Simian T-lymphotropic virus 2 | Deltaretrovirus | STLV2 | Non-human primate | NC_001815 |
| Walleye dermal sarcoma virus | Epsilonretrovirus | WDSV | Fish | NC_001867 |
| Walleye epidermal hyperplasia virus type 1 | Epsilonretrovirus | WEHV1 | Fish | AF133051 |
| Walleye epidermal hyperplasia virus type 2 | Epsilonretrovirus | WEHV2 | Fish | AF133052 |
| Atlantic salmon swim bladder sarcoma virus | Gamma-epsilon | SSSV | Atlantic salmon | NC_007654 |
| Feline leukemia virus | Gammaretrovirus | FeLV | Cat | NC_001940 |
| Friend murine leukemia virus | Gammaretrovirus | F-MuLV | Mouse | NC_001362 |
| Mus dunni endogenous retrovirus | Gammaretrovirus | MDEV | Mouse | AF053745 |
| Porcine endogenous retrovirus A | Gammaretrovirus | PERV-A | Pig | AJ293656 |
| Rhinolophus ferrumequinum retrovirus | Gammaretrovirus | RfRV | Greater horseshoe bat | JQ303225 |
| Equine infectious anemia virus | Lentivirus | EIAV | Horse | NC_001450 |
| Feline immunodeficiency virus | Lentivirus | FIV | Cat | NC_001482 |
| Human immunodeficiency virus 1 | Lentivirus | HIV1 | Human | NC_001802 |
| Visna/Maedi virus | Lentivirus | VMV | Sheep | NC_001452 |
| Bovine foamy virus | Spumavirus | BFVbta | Cattle | NC_001831 |
| Equine foamy virus | Spumavirus | EFVeca | Horse | NC_002201 |
| Feline foamy virus | Spumavirus | FFVfca | Cat | NC_001871 |

| | | | | |
|---|---|---|---|---|
| Brown greater galago prosimian foamy virus | Spumavirus | SFVocr | Greater galago | KM233624 |
| White-tufted-ear marmoset simian foamy virus | Spumavirus | SFVcja | Common marmoset | GU356395 |
| Squirrel monkey simian foamy virus | Spumavirus | SFVssc | Squirrel monkey | GU356394 |
| Orangutan Simian foamy virus | Spumavirus | SFVppy | Pongo pygmaeus pygmaeus | AJ544579 |
| Macaque simian foamy virus | Spumavirus | SFVmcy | Macaque | NC_010819 |
| African green monkey simian foamy virus | Spumavirus | SFVcae | African green monkey | NC_010820 |
| Western chimpanzee simian foamy virus | Spumavirus | SFVpve | Western chimpanzee | NC_001364 |
| Western lowland gorilla simian foamy virus | Spumavirus | SFVggo | Western lowland gorilla | NC_039029 |
| Spider monkey simian foamy virus | Spumavirus | SFVaxx | Spider monkey | NC_039027 |
| Sloth endogenous foamy virus | Spumavirus | SloEFV | Sloth | ABVD02350954 |
| Sphenodon punctatus endogenous foamy virus | Spumavirus | ERV-Spuma-Spu | Tuatara | Ref. 1 |
| Coelacanth endogenous foamy-like virus | Spumavirus | CoeEFV | Coelacanth | Ref. 2 |
| Amphilophus citrinellus foamy-like virus | Spumavirus | AciFLERV_1 | Midas cichlid | CCOE01002251 |
| Amphilophus citrinellus foamy-like virus | Spumavirus | AciFLERV_2 | Midas cichlid | CCOE01002087 |
| Austrofundulus limnaeus foamy-like virus | Spumavirus | AliFLERV | Annual killifish | Ref. 3 |
| Notophthalmus viridescens | Spumavirus | NviFLERV | Eastern newt | Ref. 3 |
| latyfish endogenous retrovirus | Spumavirus | PlatyfishEFV | Platyfish | Ref. 4 |
| Danio rerio foamy virus | Spumavirus | DrFV-1 | Zebrafish | CABZ01054182 |
| Cynops pyrrhogaster foamy-like virus | Spumavirus | CpyFLERV_1 | Japanese fire belly newt | FS313726 |
| Cynops pyrrhogaster foamy-like virus | Spumavirus | CpyFLERV_2 | Japanese fire belly newt | FS296312 |
| Pleurodeles waltl foamy-like virus | Spumavirus | PwaFLERV | Iberian ribbed newt | JG015238 |
| Poecilia reticulata foamy-like virus | Spumavirus | PreFLERV | Guppy | AZHG01028727 |
| Poecilia formosa foamy-like virus | Spumavirus | PfoFLERV_1 | Amazon molly | AYCK01023761 |
| Poecilia formosa foamy-like virus | Spumavirus | PfoFLERV_2 | Amazon molly | AYCK01027102 |
| Larimichthys crocea foamy-like virus | Spumavirus | LcrFLERV | Large yellow croaker | JRPU01021077 |
| Stegastes partitus foamy-like virus | Spumavirus | SpaFLERV | Bicolor damselfish | JMKM01038484 |

| | | | | |
|---|---|---|---|---|
| Fundulus heteroclitus foamy-like virus | Spumavirus | FheFLERV | Mummichog | JXMV01100753 |
| Lates calcarifer foamy-like virus | Spumavirus | LcaFLERV | Barramundi | LBLR01010097 |
| Gadus morhua foamy-like virus | Spumavirus | GmoFLERV_1 | Atlantic cod | CAEA01131311 |
| Gadus morhua foamy-like virus | Spumavirus | GmoFLERV_2 | Atlantic cod | CAEA01539013 |
| Dicentrarchus labrax foamy-like virus | Spumavirus | DlaFLERV | European bass | CBXY010016181 |
| Oreochromis niloticus foamy-like virus | Spumavirus | OniFLERV | Nile tilapia | AERX01018483 |
| Cynoglossus semilaevis foamy-like virus | Spumavirus | CseFLERV | Tongue sole | AGRG01002780 |
| Sebastes rubrivinctus foamy-like virus | Spumavirus | SruFLERV | Flag rockfish | AUPQ01030678 |
| Sebastes nigrocinctus foamy-like virus | Spumavirus | SniFLERV | Tiger rockfish | AUPR01019601 |
| Pimephales promelas foamy-like virus | Spumavirus | PprFLERV_1 | Fathead minnow | JNCD01073002 |
| Pimephales promelas foamy-like virus | Spumavirus | PprFLERV_2 | Fathead minnow | JNCD01029789 |
| Thunnus orientalis foamy-like virus | Spumavirus | TorFLERV | Pacific bluefin tuna | BADN01112239 |
| Periophthalmus magnuspinnatus foamy-like virus | Spumavirus | PmaFLERV | Mudskipper | JACL01052273 |
| Periophthalmodon schlosseri foamy-like virus | Spumavirus | PscFLERV | Mudskipper | JACM01000693 |
| Anoplopoma fimbria foamy-like virus | Spumavirus | AfiFLERV | Sablefish | AWGY01041462 |
| Cyprinus carpio foamy-like virus | Spumavirus | CcaFLERV | Common carp | LN590673 |
| Nothobranchius furzeri foamy-like virus | Spumavirus | NfuFLERV | Turquoise killifish | JNBZ01063262 |
| Esox lucius foamy-like virus | Spumavirus | EluFLERV | Common pike | AZJR02000232 |
| Callorhinchus milii foamy-like virus | Spumavirus | CmiFLERV_1 | Australian ghostshark | XM_007890932 |
| Callorhinchus milii foamy-like virus | Spumavirus | CmiFLERV_2 | Australian ghostshark | AAVX02030290 |
| Hynobius retardatus foamy-like virus | Spumavirus | HreFLERV | Hokkaido salamander | LE148029 |
| Rhinatrema bivittatum endogenous foamy virus | Spumavirus | ERV-Spuma-Rbi | Rhinatrema bivittatum | Ref. 6 |
| Ambystoma mexicanum endogenous foamy virus type I | Spumavirus | ERV-Spuma.a-Ame | Ambystoma mexicanum | Ref. 6 |
| Ambystoma mexicanum endogenous foamy virus type II | Spumavirus | ERV-Spuma.b-Ame | Ambystoma mexicanum | Ref. 6 |

| | | | | |
|---|---|---|---|---|
| Spea multiplicate endogenous foamy virus | Spumavirus | ERV-Spuma-Smu | Spea multiplicate | Ref. 6 |
| Ciconia boyciana endogenous foamy virus | Spumavirus | ERV-Spuma-Cbo | Ciconia boyciana | Ref. 7 |
| Ciconia maguari endogenous foamy virus | Spumavirus | ERV-Spuma-Cma | Ciconia maguari | Ref. 7 |
| Scyliorhinus torazame endogenous foamy virus type I | Spumavirus | ERV-Spuma.a-Sto | Scyliorhinus torazame | Ref. 8 |
| Scyliorhinus torazame endogenous foamy virus type II | Spumavirus | ERV-Spuma.a-Sto | Scyliorhinus torazame | Ref. 8 |
| Pristis pectinate endogenous foamy virus | Spumavirus | ERV-Spuma-Ppe | Pristis pectinate | Ref. 8 |
| Amblyraja radiata endogenous foamy virus | Spumavirus | ERV-Spuma-Ara | Amblyraja radiata | Ref. 8 |
| Snakehead retrovirus | Unclassified | SnRV | Fish (snakehead fish) | NC_001724 |
| Astyanax mexicanus lokiretrovirus | Lokiretroviruses | Loki-Ame | Astyanax mexicanus | Ref. 5 |
| Seriola rivoliana lokiretrovirus | Lokiretroviruses | Loki-Sri | Seriola rivoliana | Ref. 5 |
| Oplegnathus fasciatus lokiretrovirus | Lokiretroviruses | Loki-Ofa | Oplegnathus fasciatus | Ref. 5 |
| Anguilla rostrata lokiretrovirus | Lokiretroviruses | Loki-Aro | Anguilla rostrata | Ref. 5 |

**Table S4. The tBLASTn results of accessory genes of TraEFVs.**

| TraEFV_name | Similarity | Length | Genomic region | Genomic region represented | Query(accession_number) |
|---|---|---|---|---|---|
| TraEFVtja.a1 | 25.714 | 35 | 8270-8374 | taf | NC_001364.1_Western_chimpanzee_SFV |
| TraEFVtja.a1 | 46.154 | 26 | 9354-9431 | Borf-2 | NC_001831.1_BFV |
| TraEFVtja.a1 | 31.481 | 54 | 9315-9473 | bel2 | NC_039023.1_Brown_greater_galago-PSFV |
| TraEFVtja.a1 | 25.714 | 35 | 8270-8374 | tas | NC_039024.1_Central_cimpanzee-SFV |
| TraEFVtja.a1 | 25.714 | 35 | 8270-8374 | tas | NC_039025.1_Eastern_chimpanzee-SFV |
| TraEFVtja.a1 | 38.462 | 39 | 8261-8374 | tas | NC_043445.1_Guenon_SFV |
| TraEFVtja.a3 | 27.027 | 37 | 7525-7635 | taf | NC_001364.1_Western_chimpanzee_SFV |
| TraEFVtja.a3 | 23.729 | 59 | 7519-7695 | tas | NC_039024.1_Central_cimpanzee-SFV |
| TraEFVtja.a3 | 25.424 | 59 | 7519-7695 | tas | NC_039025.1_Eastern_chimpanzee-SFV |

| | | | | | |
|---|---|---|---|---|---|
| TraEFVtja.a3 | 35 | 40 | 7516-7635 | tas | NC_043445.1_Guenon_SFV |
| TraEFVtka.a1 | 27.027 | 37 | 10817-10927 | taf | NC_001364.1_Western_chimpanzee_SFV |
| TraEFVtka.a1 | 23.729 | 59 | 10811-10987 | tas | NC_039024.1_Central_cimpanzee-SFV |
| TraEFVtka.a1 | 25.424 | 59 | 10811-10987 | tas | NC_039025.1_Eastern_chimpanzee-SFV |
| TraEFVtka.a1 | 28.571 | 77 | 10700-10927 | tas | NC_043445.1_Guenon_SFV |
| TraEFVtka.a2 | 25.714 | 35 | 12783-12887 | taf | NC_001364.1_Western_chimpanzee_SFV |
| TraEFVtka.a2 | 25.714 | 35 | 13897-14001 | taf | NC_001364.1_Western_chimpanzee_SFV |
| TraEFVtka.a2 | 46.154 | 26 | 16078-16155 | Borf-2 | NC_001831.1_BFV |
| TraEFVtka.a2 | 46.154 | 26 | 14982-15059 | Borf-2 | NC_001831.1_BFV |
| TraEFVtka.a2 | 34.694 | 49 | 14943-15086 | bel2 | NC_039023.1_Brown_greater_galago-PSFV |
| TraEFVtka.a2 | 32.653 | 49 | 1248-1391 | bel2 | NC_039023.1_Brown_greater_galago-PSFV |
| TraEFVtka.a2 | 29.63 | 54 | 120-278 | bel2 | NC_039023.1_Brown_greater_galago-PSFV |
| TraEFVtka.a2 | 34.694 | 49 | 16039-16182 | bel2 | NC_039023.1_Brown_greater_galago-PSFV |
| TraEFVtka.a2 | 25.714 | 35 | 12783-12887 | tas | NC_039024.1_Central_cimpanzee-SFV |
| TraEFVtka.a2 | 25.714 | 35 | 13897-14001 | tas | NC_039024.1_Central_cimpanzee-SFV |
| TraEFVtka.a2 | 25.714 | 35 | 13897-14001 | tas | NC_039025.1_Eastern_chimpanzee-SFV |
| TraEFVtka.a2 | 25.714 | 35 | 12783-12887 | tas | NC_039025.1_Eastern_chimpanzee-SFV |
| TraEFVtka.a2 | 38.462 | 39 | 13888-14001 | tas | NC_043445.1_Guenon_SFV |
| TraEFVtka.a2 | 38.462 | 39 | 12774-12887 | tas | NC_043445.1_Guenon_SFV |

**Dataset S1.** The alignments used to build the phylogenetic trees for RT, POL, GAG, ENV, and POL-ENV.

##RT alignment

>AciFLERV_1

QYPLNPGAVKEMDLIVRELLTLGVIRSPIQAVKKPEGGWRPVINFKALNRRTVANRASLINPQGTLKTLKLKPYKSCIDL

ANGFFSLRLARTAFTHGRSYVWERLPQGYKNSPNVFQAAVMDVLDGLGATIYIDDVFIADDTEEQHLDRLQKIVEAGLKL

KKCQFGRKVDYLGFQV

>Afi_FLERV

QYPIKPEVKKELDL---ELEIIAKIQLPVMGVPKPDGTHRLVHNLIELNKRSFSDARHLINPSRTMRTLPQKLYKTSLDL

SNGFWSIPIDPTCFTWVESYCWLRLPMGYKNAPNVFQTAVVKTLEDLPVEVSIDDVYFTHDDPEEHLQCLEEVLRAGFKI

KKCEIARHLAYLGFKI

>AliFLERV

QYPLHPEAVAEMDKIVKELHALGIIRSPIQAVKKPEGGWRPVINFKALNRRTVANRASLINPQGALKTLRVKKYKSCIDL

ANGFFSLRLARTAFTHGKSYVWQRLPQGYRNSPNVFQSAVLEILEDVGASVYIDDVFIADDTEEEHLKRLEEVVKAGLKL

AKCQFGQFVNYLGFQV

>ALV

QWPLPEGKLVALTQLVEKELQLGHIETPVFVIRKASGSYRLLHDLRAVNAKLVAVQQGAPVL----SALPRGWPLMVLDL

KDCFFSIPLAEFAFTLARRFQWKVLPQGMTCSPTICQLIVGQVLEPLCMLHYMDDLLLAASSHDRLEAAGEEVISAGFTI

DKVQREPGVQYLGYKL

>BaEV

QYPMSLEAHMGIRQHIIKFLELGVLRTPLLPVKKPGQDYRPVQDLREINKRTVDIHPTVPNPYNLLSTLKDYSWYTVLDL

KDAFFCLPLAPFAFEWSGQLTWTRLPQGFKNSPTLFDEALHRDLTDFTLLQYVDDLLLAAPTKKACTQGTRHLLQKGYRA

KKAQICQTVTYLGYIL

>BDFF02022209_Cbo

QFKINPQAISSIQIVINYLLKQGVPRTPVYPVPKGEGKWRLVLDYRAVNKVTPAIAAQNCHSTGILMQLTRKKYKTTLDL

SRWVFGPILSPQPLLGAKQHVWTQLPQGFLNSPALFSADVVDLLKEFDVSVYVNDIYFSHDTEKEHLKTTRHLHNRGQI-

KKSEIGKRVNFLGFAI

>BFVbta

QYHINPRAKADIQIVIDDLLRQGVLRTPVYPVPKADGRWRMVLDYREVNKVTPLVATQNCHSASILNTLYRGPYKSTLDL

ANGFWAHPIKPTAFTWGKTYCWTVLPQGFLNSPALFTADVVDILKDINVQVYVDDVYVSSATEQEHLDILETIFNAGYIV

KKSKLAKEVEFLGFSI

>CcaFLERV

QYKINSAAVNDIRETIKELTEQGIIRAPLQAVPKADGSWRLVTNFKALNKVTVPDTRYLINCSETCADIGRDRWLSKIDL

ANGYWSIPLAETCFTFGDQYEYRFLPQGYRNSANAFQSSMLKILAGLPVPV-IDDILLAMSSEDEHLELLDRTLGAGLKP

KKMEIGKHVDFLGFKL

>ChrEFV

QNHINHKATPSIQ-VIDNLLAQQLKKTPVYPVPKSEGKWRMVLDYRAINKVIEPIAAQNSFSTSILAQLPKKKFKTTLDL

SN--FAIPIHPTAFT-SLHHVWTRLPQSFINIPALFPADIQQLVLSI-------SLQIQSNSYNKYISEMQDVS------

PKSQPLG-IQFLSVLK

>CmiFLERV_1

QYKIRREDKETIGKIINNLMEQGVIKSPIFLKKKPDGSWRLLLDCKALNECTNPKQGQSISSHGSIEKLTREKYHTTLNI

ANGFWSIPIVETAFTYGQQYQWTRLPEGWCNSTVIFNEAIRRVLDDNKITRFGGVIHFSNDNAESHIKLLKQILEHGLKI

RKSQIGRYVDFLGHQI

>CmiFLERV_2

------RVI--------ELERRDIITSSLLPVHKADGSWQLVVDFRQLNTVTETAVGHSIHLSGDLDNLPKGRYKITLDL

ANRFWSVPLAKTAFT-HGQFYWNQMPQGWKYSPAILNKCQQQTVIGIGVICYVDDIFWADDSPTEHLKKLDGVLELGFNI

KKSQISRYVDYLGHTV

>CoeEFV

QYPINTKAIPSIQVVINELLEQGVLVMAVLPVPKPDGTWRLVLDYRALNKHSEPVRAQNQHSSGILANIERKAYKSSVDL

ANGFWSHPIREMAFTWGFQYLWTRLPQGFLNSPALFSADVISLVGQLGVYCYVDDIYLTHDTEEEHLKILDQVLEAGYVI

KKSKLCRKVDFLGFSL

>CpyFLERV_1

QYPIKGEARASVLEILQQLEYQGVIENPLFPVIKPDGTHRLVFDYRHLNGHCRTFAVQNTHGSALVDSLVRKKYKTTLDI

SNGFYSHNLDPTGFS-GKQKLFKRLPMGYKNSPGVFSARIITLLQLIEAFSYVDDIYLTDDEMMAHLERVGHLIVIGYKF

AKSHIAHLVLFLGYEL

>CpyFLERV_2

QYPIKQEAKKQVREILDQLEYQGVIENPLFPVAKPDNSYRLVLDYRHLNS---TLAVQNAHSAALMNNIVRKKYKTTLDT

SNGFFCQNIAPTAFS-GTQRQFCRLPQGFKNSPGLFSARV-----------YVDDIYLTDDDLDRHLNRVSTIILEGYK-

----------------

>CseFLERV

QYPLNPGAVQEMDMIV-----LGIIRSPIQAVKKPEGGW-PVINFKALNRRTVASRASLINPQGALKTLKVKKY-SCIDL

AN-FFSLRLTRTAFTHGKAYVWQRLPQGYKNSSNVFQSVVTDVLDGLDVTAHIDDVFIADDTEE-HLEKLKQVIQTGLKL

KKCQLGRFVDYLGFQV

>EFVeca

QYRINPKAKADIQIVIDDLLKQGVLKTPVYPVPKPDGRWRMVLDYRAVNKVTPAIATQNCHSASLLNTLYRGQYKTTLDL

ANGFWAHPIQETSFTWGKSYVWTTLPQGFLNSPALFTADVVDLLKDINVEVYVDDVYFSNDTEEEHLKTMDLLFQAGYIV

KKSKLGQHVDFLGFQI

>EIAV

QWPLTKEKLEGAKEIVQRLLSEGKISSPIFVIKKRSGKWRLLQDLRELNKTVQEISRGLPHP----GGLIKCKHMTVLDI

GDAYFTIPLDPTAFTIDKRYVWKCLPQGFVLSPYIYQKTLQEILQPFQLYQYMDDLFMGSNSKKQHKELIIELRAKGFET

DKLQEVPPYSWLGYQL

>ERV-Spuma-Ara

QYQIKPEARRAIQEVINDLVEQGVLVTPILAVAKPGGEYRLVCDYRALNKGSVKFAGTLFNMASQTMQIQRH-YKTTFDL

ANGFWSHPIPETAIT-GVQYCWTRLPQGFVNSPQIFSAQVLRTLDFLIIECYVDDIFTTHATLDDHFNCLHKILQLGYRV

KKSAISKTVKFLGIEI

>ERV-Spuma-Ppe

QWYIKQRGKQSLQETVGSLCYKNILNHPCLVENLTDGDWSSSINTQSLNKLCK---------DSLVKSIN---LAQTLMA

SDQFIIQPLQP-----CSQYQWTRRPQVFRNSPQILSAGVIQTLQGIVNCVYVDNIDKAD-TLEEHLTQIETVIKRGYVI

KKSQLCQTVEFLGTSI

>ERV-Spuma-Rbi

QYPVHKKFYNDLNIIISQKLTQGVLISPLLPVPKPNGSTRMVIDYRVLNASSETVAAQTLNPASTIDNLPRPRWKATLDL

ANGFWSIPISTTAFTFGKQYQYTRLPQGFKNSPVLFSAAVEELLQNINAVPYVDDIYLASD----KLDVLDSVVQEGFVV

AKARIGFEVKFLGFLI

>ERV-Spuma-Smu

QYKINHAAIPSIQIVIDDLVKQGIIKTAVYPVPKPNGSWRLVLDYREVNKVTPVVSAQNTHSVSILHGLVRKRFKTTLDL

ANGFWSHPIDTTAFTWGKQYVWTRLPQGFVNSPALFTADVVSLLSAFNIEVYVDDIYLSHDSEEEHIAVLTQVLDAGYIV

KKSQIARNVTFLGFDI

>ERV-Spuma.1-Cma

QFKINPQAIPSIQIVINYLLKQGIPRTPVYPVPKGEGKWRLVLDYRAVNKVTPAIAAQSCHSTGILMQLTRKKYKTTLDL

SRWVFGPILSPIAFTWGKQHVWTQLPQGFLNSPALFSADVVDLLKEIDVSVYVNDIYFLHDTEKEHLKTTRHLHNRGQI-

KKSEIGKRVNFLGFAI

>ERV-Spuma.a-Ame

QYPVKPQAVAHMQTIVDQLMDQGVIISPVLPVLKPDGTYRFZVDFRILNAHCKTEAVQNQHSSGLLANLHRCKYKTNLDL

GNGYFTQPLSVTAFTFGTHFQFTRIMQGYKNSPSTZSSRIQSLLADINCWQYVDZIYITHENLSEHLQEVDRVIZKGYLI

KKSRIAHZVTFLGFTL

>ERV-Spuma.a-Sto

QWFIDPECRAALQKTIDSLCQQGVLISPMFGIPKAKGGVRLLVDYRELNRVTIPNAAQSASIQQVTQQISRKKYKSTLDL

SNGFWSHPILPTAFT-GHQYVWTRLPQGFRNSPQLFAAGVISALAPIDIDVYVDDVYVTTDTREQHLEALDEIFTHGYIV

KKCQLLKKVEYLGINV

>ERV-Spuma.b-Ame

QYPTPLPAZPHIQKTITQLVDQGVIITPVLPVIKPDHTYRLVLDYRELNAHARTDAVQNLHZSGLLNNILRNKYKTVLDL

GZGFFAQPLAPTAFTHGKHFQMTRLMQGYKNSPGIFSSRIQTLLADINCWQYVDDIYCTDDDLEEHLERVDQIVRKGYIF

KKSLIAHZVTFLGFTL

>ERV-Spuma.b-Sto

QWHIEHRGRASLIKTIDSLEQQGVLISPMFGVAKADNKWRLVIDYTKLNKYTEPNSAQQANAQQMVSQLVRKKYKSCVDL

ANGFWSHPIKETAFT-GYQYVWTRLPQGFRNSPQIFSARVMEILSDLQVTSYVDDIYLTTDBLDEHFELLNEVFKNGYLI

KKSQLCRQVNFLGLSV

>FeLV

QYPMPHEAYQGIKPHIRRMLDQGILKTPLLPVKKPGEDYRPVQDLREVNKRVEDIHPTVPNPYNLLSTLPSHPWYTVLDL

KDAFFCLRLHSFAFEWSGQLTWTRLPQGFKNSPTLFDEALHSDLADFVLLQYVDDLLLAAATRTECLEGTKALLEKGYRA

KKAQICLQVTYLGYSL

>FFVfca

QYHINPKAKPDIQIVINDLLKQGVLITPVYPVPKPNGRWRMVLDYRAVNKVTPLIAVQNQHSYGILGSLFKGRYKTTIDL

SNGFWAHPIVPTAFTWGKQYCWTVLPQGFLNSPGLFTGDVVDLLQGINVEVYVDDVYISHDSEKEHLEYLDILFNAGYII

KKSNIANSVDFLGFQI

>FIV

QWPLTNEKIEALTEIVERLEREGKVKTPVFAIKKKSGKWRMLIDFRELNKLTEEVQLGLPHP----AGLQIKKQVTVLDI

GDAYFTIPLDPTAFTLGRRFVWCSLPQGWILSPLIYQSTLDNIIQPFDIYQYMDDIYIGSNSKKEHKEKVEELRKWGFET

DKLQEEPPYTWMGYEL

>FMuLV

QYPMSQEARLGIKPHIQRLLDQGILVTPLLPVKKPGNDYRPVQDLREVNKRVEDIHPTVPNPYNLLSGLPSHQWYTVLDL

KDAFFCLRLHPFAFEWSGQLTWTRLPQGFKNSPTLFDEALHRDLADFILLQYVDDLLLAATSELDCQQGTRALLQLGYRA

KKAQICQKVKYLGYLL

>HIV1

QWPLTEEKIRALTDICTEMEKEGKISTPVFAIKKKDTKWRKLVDFRELNKRTQEVQLGIPHP----AGLKKKKSVTVLGV

GDAYFSVPLDPTAFTIGIRYQYNVLPQGWKGSPAIFQCSMTKILEPYIIYQYMDDLYVGSDEIEQHRAKIEGLRDWGFTT

KKHQKEPPFLWMGYEL

>HIV2

QWPLTKEKIEALKEICEKMEKEGQLETPTFAIKKKDNKWRMLIDFRELNKVTQEIQLGIPHP----AGLAKKKRISILDV

GDAYFSIPLHETAFTLGKRYIYKVLPQGWKGSPAIFQYTMRQVLEPFILIQYMDDILIASDTGLEHDKVVLQLKELGFST

EKFQKDPPFQWMGCEL

>HTLV1

QFPLNPERLQALQHLVRKALEAGHIENPVFPVKKANGTWRFIHDLRATNSLTISSSPGPPDL----SSLPTTAHLQTIDL

KDAFFQIPLPKFAFTVGTRYAWRVLPQGFKNSPTLFEMQLAHILQPITILQYMDDILLASPSHADLQLLSEATMAHGLPV

NKTQQTPGIKFLGQII

>TraEFVtka.a4

QYHVNPKAKPDIQIVINDLLKQGVLVTPIYPVPKSNGKW-MVLDYREVNKVTPLIATQKQHSAGVLSSLYRGKYKSAIDL

ANGFWAHPITRTAFTWGKQYCWTVLPQGF-NSPAVFTGDVVDLLK-ITVQVYVDDVYLRHDTLKEHLDSLRKMFQVGYIV

HKSALCQSVEFLGFQI

>TraEFVtka.b1

QYHINPKARSDIQIVIDDLIKQGVLITPVYPVPKGNGKWRMVLDYREVNKVTPLVATQNCYSTGILASIYREKYKSVIDL

ADGFWAYRIMRTAFTW--NYFWTVLPQGFLNSPALFTGDVVNLLKGINIQVYIDDVYLSPDTSEEHYDALNQVFTAGYIV

HKSQFFQKVEFLGLQI

>TraEFVtka.b2

KYGIYPKTKLDIQIVINELIKQGFFNNILYP-----REWKLNFFHGAVNKLTPINAIHYCYSTRILALIYWGKYKSTINL

VNGFWAHPILNKIFALGKEYCWIVLPQVFLKSPVLFTGAIGDLRKEISVQVYVGDVCLSHDTFEECYNALDQMLMERYTI

HKSQLFQKLNILGPGL

>TraEFVtka.b4

---------------------------------------------REVNKVTPIIATPNCHSTGISASMYRGKYKSTIDL

ANGFGAHPIARTAFTWGKQYCWTVLPQGFINSPALFTGDIVDLLKEINVQVYVDDVYLSHDTFEEHYNALGQMLMAGYIV

HKSQLFQKVEFLGFQV

>TraEFVtka.b5

KYHINPKAKPDIQIVINDPIRQGVLITPVYPVPKGNRK-RMILDYQKINEVTPIIATPNCHSTRILASIYREKYKSTIDL

ANVFSPHPIARTAFT-EKQYYWTVLPQGFLNSPALFTGNIVDLLKEINVQVYVDDMYLSHDMFEEHYNVLDQMLMTGYIV

HKYQLFLKVKFLGFQI

>TraEFVtka.a3

QYYINPKAKPNIQIVINNL-KQRILLTPIYPVPKNNER-KMVLNYREVNKVTPLITT-NQHSAEILSSLYRDKYKSTIDL

TNDFWAHSTTRTTFT-KKQYC-TVLPQNFLYSPASFTRNVVDLLKEINVQVYVDDVYLSHDIFKKHLDTLKKMLQIRYIV

QTSTLCQSVEFLKFQI

>TraEFVtka.a1

QYHINPKAKPDIQIIVNDLLKQGILITPIYPVPKNSGKWRMVLDYREVNKVTPLIATQNQHSAGILSSLYRGKYKSTIDL

ANGFWAHPITRTAFTWGKQYCWTVLPQGFLNSPALFTGDVVDLLKEINVQIYVDDVYLRHDTFKEHLDALRKMLQAGYIV

QKCALCQSVEFLGFQI

>TraEFVtka.a2

QYHINPKAKPDMQIVVNDLLKQGVLITPVYLVPKSNGRWRMVLDYREVNKVTPLIATQNQHSAGILSSLYRGKYKSTIDL

ANGFWAHPITRTAFAWGKQYCWMVLPQDFI---ALLC-SLRMLMYEINVQVYVDDVYLSNDTIEEHLDALRKMFQAGYIV

QKSALCQSIEFLGFQI

>KoRV

QYPMSKEAREGIRPHIQRFLDLGILVTPLLPVKKPGNDYRPVQDLREVNKRVQDIHPTVPNPYNLLSSLPSHTWYSVLDL

KDAFFCLKPHPFAFEWTGQLTWTRLPQGFKNSPTLFDEALHRDLASFVMLQYVDDLLVAAPTYRDCKEGTRRLLQLGYRV

KKAQLCREVTYLGYLL

>LcaFLERV

QYPLNPGAVEEMDTIVKELSALGIIRSPIQAVKKPEGGWRPVINFKALNRRTVANRASLINPQGTLKTLQVKRFKSCIDL

ANGFFSLSLARTAFTHGKFYVWQRLPRGYKNSPNVFQSAVMEVLDGLGATIYIDDVFIADDTEEL---------------

-------F--------

>LcrFLERV

QYPLNPGAVEEMDKIVTELSALDIIRSPIQAVKKPEGGWRPVINFKALNRRTVANRASLINPQGALKTLQVKRFKSCIDL

ANGFFSLRLAKTAFTHGKAYVWQRLPQGYKNSPNVFQAAVMDVLKDLGVTIYIDDVFLADDTEEEHLQRLRQVVEAGLKL

KKCQFGQFVNYLGFQV

>LDV

QWPLTAQKLDAVQNIIQDLLKDGRIISPIFVIQKKDSKFRMLHDLRAVNALIKALQPGTPWP----GAIPSEWPVIAMDI

SDCFFSIPLAEFAFTIAKRYQWTVLPQGMKNSPYICQQVVAEVIRPIVIIHYMDDILIAAAEERQTEVIFEAVKTKGLKI

AKTQRAPEVSYLGWRV

>LmeFLERV

----------------------------------------------------------DQHSSGILANIERKAYKSSVDL

TNGFWSHPIREMAFTWSFQYLWTCLP-GFLNSPALFSADVISPVGQLGVYCYVDDIYLTHDTEEEHLKILDQVLEAGYVI

KKSKLCRRVDFLGFSL

>loki_Ame_Smc

QYKIPLASYGPVQEIIDDLLDKGIIRAPLWPVLKPNGKWRLTIDYRRLNDQVP---------TQLEQELPRVRYFSTLDV

ASGFWTIPVHVLAFTFGRQFTFTRCPFGYSNSPAEFNIFLNKACPDAGTLIYVDDVLIRNNSLDAHLEEIDHVLDAGAKI

AKCQWCKTVNYVGLLV

>loki_Aro

KYRIPIAAHDPVQDTITEILHAGIIRSPIWPVLKPSGKQSLTVDYRKLNKQVP---------WM--KPLQ-KRTSQAXDI

SNGFCTIPVRELAFSFNVQYTWTVTPFGYANSPAEFNIFFHKAIPDVNIAIYVDDILMWSDSWGEHQQLLSYVLTAGAKI

DKGQWCKRVNYLGFQV

>loki_Ofa

QYRIPLAAYESIQEILDNLLQKQIIRSPIWPVLKPTGKWRLTIDYRPLNKQVP---------IHLDQELAKVKFFSTVDV

ANGFWTMRVNPLAFSFNRQFTWNRCPFGYSNSPAEFNIFLHKAMSDAGNLIYVDDILMRSRTLXEHLAEIRHVLNAGAKL

AKGQWCRTVEYVGLTV

>loki_Sri

QYKIPLASYEPVQEIIDNLLEKGIIRAPLWPVLKPNGKWRLTIDYRKLNQQVP---------TQLEQELPKIRYFSTLDI

ASGFWTIPVHVLAFTFNRQYTFTRCPFGYANSPAEFNIFLNKACPDAGTLIYVDDILMRSSTLDTHLEEIDHVLEAGAKI

SKCQWCKTVNYVGLLV

>MMTV

QWPLKQEKLQALQQLVTEQLQLGHLETPVFVIKKKSGKWRLLQDLRAVNATMHALQPGLPSP----VAVPKGWEIIIIDL

QDCFFNIKLHPFAFSVYQRFQWKVLPQGMKNSPTLCQKFVDKAILTVYIVHYMDDILLAHPSRSIVDEILTSMIQHGLVV

EKIQKYDNLKYLGTHI

>MMuLV

QYPMSQEARLGIKPHIQRLLDQGILVTPLLPVKKPGNDYRPVQDLREVNKRVEDIHPTVPNPYNLLSGLPSHQWYTVLDL

KDAFFCLRLHPFAFEWSGQLTWTRLPQGFKNSPTLFDEALHRDLADFILLQYVDDLLLAATSELDCQQGTRALLQLGYRA

KKAQICQKVKYLGYLL

>MPMV(SRV3)

QWPLTNDKLAAAQQLVQEQLEAGHITTPIFVIKKKSGKWRLLQDLRAVNATMVALQPGLPSP----VAIPQGYLKIIIDL

KDCFFSIPLHPFAFSLMQRFQWKVLPQGMANSPTLCQKYVATAIHKVYIIHYMDDILIAGKDGQQVLQCFDQLKQAGLHI

EKVQLQDPYTYLGFEL

>NviFLERV-1

QYPIKREAKASVKEILTHLENQDVIENPLFPVAKPDHSYHIVLDYRHLNSHTRSFAIHNTHSSTLVNNLTRKKYKTTLGI

SN-FFTQNLAPSTFT-GIQYKFWRLPMGYKNSLGVFAVRIIELLQLVDAVSYIDYIYVTDDEMQQHLARVDRIIISGYKI

KKSKIAFFVLFLGYKL

>NviFLERV-2

QYPIKKEAKASVKEILKQLEYQGVIENPLFPVVKPDNTYRIVLDYRHLNSCTRSFAIQNTHSSGLVNNLTRKKWKTTLDI

SNGFHAQNLAESSFT-GIQFKFRRLPMGYKNSPGIFAARIINILQKIDAVSYVDDIYVTDDEIRIHLARVNKIILHGYKI

KKSKIAFFVLFLGYEL

>PERVA

QYPLSKEAREGIRPHVQRLIQQGILVTPLLPVRKPGNDYRPVQDLREVNKRVQDIHPTVPNPYNLLCALPQRSWYTVLDL

KDAFFCLRLHPFAFEWTGQLTWTRLPQGFKNSPTIFDEALHRDLANFTLLQYVDDLLLAGATKQDCLEGTKALLLLGYRA

KKAQICRRVTYLGYSL

>PfoFLERV

QYPLNPGAVEEMDKIVKELGSLGIIRSPIQAVKKPEGGWRPVINFKALNRRTIVNRASLINPQGTLKTLRVKKFKSCIDL

ANGFFSLRLARTAFTHGKSYVWQRLPQGYKNSPNVFQSAVMEVLGDVGATVYIDDVFIADDTEEEHLERLQKVIEAGLKL

KKCQFGQFVNYLGFQV

>platyfish_EFV

QYPLNPGAVEEMDKIVKELGALEIIRSPIQAVKKPEGGWRPVINFKALNRRTIANRASLINPQGALKTLRVKKFKSCIDL

ANGFFSLRLARTAFTHGKSYVWQRLPQGYKNSPNVFQSAVMEVLGDVGATVYIDDVFIADDTEEEHLERLRKVVEAGLKL

KKCQFGQFVNYLGFQV

>PprFLERV_2

QYPINKKAIPEIEETIKELGQLGVIKSPLQAVPKSDNSFRLVTNFKSLNKVSEPDTRYLINAKDVTNGLPKGKILTKIDL

ANGFWTVPLTETAFTFTKSYCYTRLPQGYMNSPNAFQSIVVSLMEGLPVTVYIDDLLIVNDDPDEHLKIVDEVLTVGFKP

KKIEIGKGVDFLGFAT

>PreFLERV

QYPLNPGAVGEMDKIVKDLGSLGIIRSPIQAVKKPEGGWRPVINFKALNRRTVANRASLINPQGTLKTLRVKKFKSCIYL

ANGFFSLRLARTAFTHGKSYVWQRLPQG-KNSPNVFQSAVMEVLGDVGATVYIDDVFIADDTEEEHLVRLRTVXEAGLKL

KKCQFGQFVNYLGFQV

>PSFVaye

QYAINPKARSSIQAVIDDLLKQGVLTTPVYPVPKPNGQWRLVLDYEVVNKPNPPLTAQNFCSIGLLTTLPKHKYKTTLDL

SSGFEAYPITEWIFL-GVMNAWTRLPQGFLNSPSLLIADVTEILKDINMVVYTDDI------------------------

----------------

>TraEFVtja.a1

QYHINPKAKPDMQIVVNDLLKQGVLITPVYLVPKSNGRWRMVLDYREVNKVTPLIATQNQHSAGILSSLYRGKYKSTIDL

ANGFWAHPITRTAFAWGKQYCWMVLPQDFI---ALLC-SLRMLMYEINVQVYVDDVYLSNDTIEEHLDALRKMFQAGYIV

QKSALCQSIEFLGFQI

>TraEFVtja.b3

QYHINPKARSDIQIVIDDLIKQGVLITPVYPVPKGNGKWRMVLDYREVNKVTPLVATQNCYSTGILASIYREKYKSVIDL

ADGFWAYRIMRTAFTW--NYFWTMLPQGFLNSPALFTGDVVNLLKGINIQVYIDDVYLSPDTSEEHYDVLNQVFTAGYIV

HKSQFFQKVEFLGLQI

>TraEFVtja.a4

QYHVNPKAKPDIQIVINDSLKQGLLVTPIYPVPESNGKWRMVLDYREVNKVTPLIATQNQHSAGILSSLYRGKYKSTIDL

ANGFWAHPITRTAFTWGKQYCWTVLPQGFLNSPALFTGDVVDLLKEINVQVYVDDVYLSHDTFEEHLDSLRKMFQAGYIV

HKSALCQSVEFLGFQI

>TraEFVtja.b4

KYHINPQVKPDIQIVTNDLIRQGALITPVYPIPKGNEKWRMVLDYGAVNKVTPIIATQNVTLQGFPP--YTGENMSAINL

ADDLRAHSICRTYFTWGKQYCWTLSPQGFLNSLASFIVDVVDLLKEINVQVYVDDVYLSHDTFEKHYNALDQMLMARYIV

HKSHLFKQVECLGFQI

>TraEFVtja.b5

-----------------------------------------------MGKVTPIIATQNCHSTGILVSIRRK-YKCTIDL

ANGFWAHPIARTGFS-GKEYCWTVLPPGFLNSPALSTRDIVDPLKEI-VQVYVDDVYLCHDTFEEHYNALDQMLMATHRV

HKSQLFQKVEFLGFQR

>TraEFVtja.b6

---------------------------------------------REVNKVTPIIATPNCHSTGILASMYREKYKSTIDL

ANGFGAHPIARTAFTWGKQYCWTVLPQGFLNSPALFTGDIVDLLKEINVQVYVDDVYLSHDTFEEHYNALGQMLMAGYIV

HKSQLFQKVEFLGFQV

>TraEFVtja.a3

LWF--PSLRNNITLIQKNLIKSSIIYTPIYPVPKNNER-KMVLNYREVNKVTPLITT-NQHSAEILSSLYRDKYKSTIDL

TNDFWAHSTTRTTFT-KKQYC-TVLPQNVLYSPASFTRNVVDLLKEINVQVYVDDVYLSHDIFKKHLDTLKKMLQTRYIV

QTSTLCQSVEFLKFQI

>TraEFVtja.b7

KYHINPKAKPDIQIVINDLIRQGVLITPVYPVPKGNRK-RMILDYQKINEVTPIIATPNCHSTRILASIYREKYKSTIDL

ANVFSPHPIARTAFT-EKQYYWTVLPQGFLNSPALFTGNIVDLLKEINVQVYVDDMYLSHDMFEEHYNVLDQMLMTGYIV

HKYQLFLKVKFLGFQI

>TraEFVtja.a2

QYHINPKAKPDIQIIVNDLLKQGILITPIYPVPKNSGKWRMVLDYREVNKVTPLIATQNQHSAGILSSLYRGKYKSTIDL

ANGFWAHPITRTAFTWGKQYCWTVLPQGFLNSPALFTGDVVDLLKEINVQIYVDDVYLRHDTFKEHLDALRKMLQAGYIV

QKCALCQSVEFLGFQI

>TraEFVtja.b1

QYHINSKAKPDIQIVINHLTKQGVLITPVYPIPEGNGKWRMVLDYREVNKVTPVITSQNYHSKGILASIYRGKYKSTIDL

ANGFGAHPIAKTAFTWGEQYCWTVLPQGFLNSPALFTGDIVDLLKEVNVQVYVDDVYLSHDMFEEHYNALDQMLMAGYIV

CKSQLF-KVEFLGFQI

>RD114

QYPMSKEAHMGIQPHITRFLELGVLRTPLLPVKKPGRDYRPVQDLREVNKRTMDIHPTVPNPYNLLSTLSDRTWYTVLDL

KDAFFCLPLAPFAFEWSGQLTWTRLPQGFKNSPTLFDEALHRDLTDFTLLQYVDDLLLAAPTEEACTRGTKHLLRKGYRA

KKAQICQTVTYLGYIL

>RfRV

QYPMSAEARKGIAPHINRLLEAGILKTPLLPVKKPGKDYRPVQDLREVNKRVEDIHPTVPNPYTLLSHLPSHVWYTTLDL

KDAFFSIALAPFAFEWPGQLTWTRLPQGFKNSPTLFNEALNQDLDSFTLLQYVDDLLLAAPSEAECRQATGDLLQLGYRA

KKAQICRQVTYLGYKL

>RMuLV

QYPISQEARLGIKPHIQRLLDQGILVTPLLPVKKPGHDYRPVQDLREVNKRVEDIHPTVPNPYNLLSGLPSHQWYTVLDL

KDAFFCLRLHPFAFEWSGQLTWTRLPQGFKNSPTLFDEALHRDLADFILLQYVDDLLLAATSELDCQQGTRALLQLGYRA

KKAQICQKVKYLGYLL

>SFVcae

QYPINPKAKASIQTVINDLLKQGVLITPVYPVPKPDGKWRMVLDYREVNKTIPLIAAQNQHSAGILSSIFRGKYKTTLDL

SNGFWAHSITPTAFTWGQQYCWTRLPQGFLNSPALFTADVVDLLKEVNVQVYVDDIYISHDDPREHLEQLEKVFSAGYVV

KKSEIAQHVEFLGFNI

>SFVcja

QYHINTKAKPSIQQVIDDLLKQGVLITPIYPVPKPDGKWRMVLDYRAVNKTVPLIGAQNQHSLGILTNLVRQKYKSTIDL

SNGFWAHPITKTAFTWGKQHVWTRLPQGFLNSPALFTADVVDLLKNIGISVYVDDIYFSTETVSEHLKILEKVFKAGYIV

KKSALLRYVTFLGFSI

>SFVggo

QYPINPKARSSIQVVIDDLLKQGVLVTPVYPIPKPDGRWGMVLDYREVNKTIPLIAAQNQHSAGILATIVRKKYKTTLVL

ANGFWAHPITPTAFIWGKQYCWTRLPQGFLNSPALFTADVVDLLKEINVQAYVDDIYLSHDDPQEHLDQLEKVFQAGYVV

KKSEVAQKVEFLGFNI

>SFVmcy

QYPINPKAKPSIQIVIDDLLKQGVLITPVYPVPKPDGKWRMVLDYREVNKTIPLIAAQNQHSAGILSSIYRGKYKTTLDL

TNGFWAHPITPTAFTWGKQYCWTRLPQGFLNSPALFTADVVDLLKEINVQAYVDDIYISHDDPQEHLEQLEKIFSAGYVV

KKSEIAQRVEFLGFNI

>SFVocr

--------------------------TPVYPVPKPDGKWRMVLDYRAVNKTVPAIGAQNCHAPGILSSLYRAKFKTTLDL

SNGFWSHPITPTAFTWGSQYVWTRLPQGFLNSPALFTADVVDLCKHINVSAYVDDIYVSNDTAEEHLRTLEQLFRAGYIV

KKSKIGVSVDFLGFEI

>SFVppy

QYPINPKAKESIQIVINDLLKQGVLITPVYPVPKPDGRWRMVLDYREVNKTIPLIAAQNQHSAGILASIYRGTYKTTLDL

ANGFWAHPITPTAFTWGKQHCWTRLPQGFLNSPALFTADVVDLMKHINVQVYVDDLYLSHDDPQEHLQVLQQVLHAGYVV

KKSAIAQKVEFLGFNI

>SFVpve

QYPINPKAKPSIQIVIDDLLKQGVLTTPVYPVPKPDGRWRMVLDYREVNKTIPLTAAQNQHSAGILATIVRQKYKTTLDL

ANGFWAHPITPTAFTWGKQYCWTRLPQGFLNSPALFTADAVDLLKEVNVQVYVDDIYLSHDNPHEHIQQLEKVFQAGYVV

KKSEIGQRVEFLGFNI

>SFVssc

QYHINPKAKPSIQIVINDLLKQGVLKTPIYPVPKTEGKWRMVLDYRAVNKTIPLIAAQNQHSAGILTNLVRQKYKSTIDL
SNGFWAHPIDQTAFTWGKQYVWTRLPQGFLNSPALFTADVVDLLKEINVNVYVDDIYVSTETINQHFQVLDKIFQAGYVV
KKSNLCRYVTFLGFTI
>SIV
QWPLSREKIEALTEICKQMEEEGKLSTPVFAIKKKDTQWRMLVDFRELNKATQEVQLGIPHP----AGLQKKKQITVIDI
GDAYYSIPLCKTAFTIGIRYQFNCLPQGWKGSPTIFQNTAANILEEIEIVQYMDDLWLASDDETRHNQQVDIVRKKGLET
KKVQREPPWEWMGYKL
>sloEFV
QYHINYKAKLAIQTVINDLIKQGVLLTPIYPVPKTNGSWRMVLNFRAVNKVIPLIAVQNQYSIEILTQMQREQYKTTLDL
SNGFWAHPIRKMAFTWGKQLVWTRLPQGFINSPALFTANIVDILKEIDVEVYVNDIYFSNVTEEQHLITLKQVLKSGYIV
KKSEIAKEVTFLSFNI
>SnRV
QYPVPDASHASIKETVEALLEQGVLRSAIWPVGKPDGSWRLTIDYRPLNSAVSCPYPTVASTPELFAKLEKKQVYSSLDI
SNGFWSIRLEEFAFTFTQQYTWTRLPQGFHASPGIFHQALYNGLAAIKLLQYVDDILLMSEDRDHHLRSLAILLQLGVKI
KKSHFCKDVQYLGVNV
>spuERV_pol
QYPINKAAINDIQKVINDLIAQGALITPVYPVPKPNGKWHMVLDYRALNRVSPSFNVQNLHV--------HARKFGKAQI

QNGFLGSSYRETAFSWGTQYCWTRLPQGYLNSPALFSADVIQLLKNIGVHSYMDDIYFTNEDLDQHLATLKQIVTAGYII

KKSQICRSVKFLGFLL

>SruFLERV

ERRLNPRAVAEIDITVKELEALGIIRSPILAKKNAEGGWKFVIDFSALNEQTIPTRASLISSQGTLKTLQVGKYKSCINL

ANGFFSLGLSETAFKRGKAYVWQRLPLGYKNSPKVFQSAVMDILGDLEVTIRTGNVFIADDTEEKHLDKLRKVIEAGLKL

KKCQFGQFVNYVGFQV

>SRV1

QWPLTSEKLAAAQQLVQEQLEAGHITTPIFVIKKKSGKWRLLQDLRAVNATMVALQPGLPSP----VAIPQGYLKIIIDL

KDCFFSIPLHPFAFSLMQRFQWKVLPQRMANSPTLCQKYVATAIHKVYIIHYMDDILIAGKDGQQVLQCFDQLKQAGLHI

EKIQLQDPYTYLGFEL

>STLV3

AVPVKPERLQALTDLVSRALEAKHIENPIFPVKKPNGKWRFIHDLRATNSLTRSPSPGPPDL----TSLPQDPHLRTIDL

TDAFFQIPLPAFAFTLGTRYSWRVLPQGFKNSPTLFEQQLSHILAPVLIIQYMDDILLASPALRELTALTKVTNAEGLPM

EKTQATPGIHFLGQVI

>TorFLERV

QHPLPPECIEQLSETIEELEKLGVITTPIMGVKEPDETWRLVHSLIALNTRSCSDTRSMINP------------------

---FLSIPMCPTAFTWRQRYGWKRQLQGLSSAPNVFQSVVEDILEELPVKIYIDGVYFTNDSEEEHLDLLRVII-AGFKI

KKCEIAKHLKFLGFDI

>VMV

QWPLTQEKLEGLKEIVDRLEKEGKVGTPIFCIKKKSGKWRMLIDFRELNKQTEEAQLGLPHP----GGLQRKKHVTILDI

GDAYFTIPLYETCFTMCVRYYWKVLPQGWKLSPAVYQFTMQKILRGWQFGIYMDDIYIGSDGLEEHRGIVNELASYGFML

DKRQEGYPAKWLGFEL

>WDSV

QYPLPKDKTEGLRPLISSLENQGILITPIFPIKKAGDEYRMIHDLRAINNIVAPLTAVVASPTTVLSNLASLHWFTVIDL

SNAFFSVPIHKFAFTFGHQYTWTVLPQGFIHSPTLFSQALYQSLHKIEICIYMDDVLIASKDRDTNLKDTAVMLQEGHKV

KKLQLCQQVVYLGQLL

>WEHV1

QYPLPKEKVEGLRPMIHSLLAQGVLTTPIFPIKKPGEEYRMIHDLRAINEIVAPLTAVVASPTTVLANLSDMTCFTVIDL

SNAFFSVPIHPFAFTFGRQYTWTVLPQGFIHSPTLFSQALFSSLSKIEICIYMDDVLIASRDEETNYKDTATMLHEGHKV

KKLQLCKSVVYLGQLI

>WEHV2

QYPLPKEKVNGLRPMIESLLAQGVLATPIFPIQKPGQEYRMIHDLRAINEIVAPLTAVVASPTTVLANLSDMKWFTVIDL

SNAFFSVPVHPFAFTFGRQYTWTVLPQGFIHSPTLFSQALFSSLSKIEVCIYMDDVLIASTTKDINIKDTVTLLHEGHKV

KKLQLCKTVIYLGQLL

##POL alignment

>BFVbta

PRPQKQYHINPRAKADIQIVIDDLLRQGVLRQQNSEMNTPVYPVPKADGRWRMVLDYREVNKVTPLVATQNCHSASILNT

LYRGPYKSTLDLANGFWAHPIKPEDYWITAFTWGGKTYCWTVLPQGFLNSPALFTADVVDILKDIPNVQVYVDDVYVSSA

TEQEHLDILETIFNRLSTAGYVSLKKSKLAKETVEFLGFSISQNGRGLTDSYKQPPTTLRQLQSILGLINFARNFLPNFA

ELVAPLYWTMDHTLKTIIQALNSTENLEERRPDVDLIMKVHISNTAGYIRFYNHGGQKPIAYNNALFTSTELKFTPTEKI

MATIHKGLLKALDLSLGKEIHVYSAIASMTKLQKTPLSERKALSIRWLKWQTYFEDPRIKFHHDATLPDLQNLPYEAIFY

TDGSAIRSPKPNKTHSAGMGIIQAKFPDFRIVHLWSFPLGDHTAQYAEIAAFEFAIRRATGIRGPVLIVTDSNYVAKSYN

EELPYWESNGFVNNKKTLKHISKWKAIAECKNLKADIHVIHEPGHQPAEASPHAQGNALADKQAVSGSYKVFSNPSLDAE

LEQVLSTPNPQGYPNKYEYKLVNGLCYVDREEGLKIIPPKADRVKLCQLAHDSAHLGRSALLLKLQQKYWWPRMHIDASR

IVLNCTVCAQTNSTNQKPRPPLVIPHDTKPFQVWYMDYIGPLPPSNGYQHALVIVDAGTGFTWIYPTKAQTANATVKALT

HLTGTAVPKVLHSDQGPAFTSSILADWAKDRGIQLEHSAPYHPQSSGKVERKNSEIKRLLTKLLAGRPTKWYPLIPIVQL

ALNNTTPHQLMYGADCNLPFENLDTLDLTREEQLAVLKEVRDGLWTPSPGLLVQERVARPAQLRPKWRKPTPIKKVLNER

TVII-DHLGQDKVVSIDNLK

>EFVeca

PKPQKQYRINPKAKADIQIVIDDLLKQGVLKQQTSPMNTPVYPVPKPDGRWRMVLDYRAVNKVTPAIATQNCHSASLLNT

LYRGQYKTTLDLANGFWAHPIQESDQWITSFTWNGKSYVWTTLPQGFLNSPALFTADVVDLLKDIPNVEVYVDDVYFSND
TEEEHLKTMDLLFQKLQTAGYVSLKKSKLGQHTVDFLGFQITQTGRGLTDSYKTPPNTLKQLQSILGLLNFARNFIPNYS
ELITPLYWETKHTLQKIIKELNASENLEQRKPDVELIVKVHVSPTAGYIKFANKGSIKPIAYHNVVFSKTELKFTITEKV
MTTIHKALLKAFDLAMGQPIWVYSPIHSMTRIQKTPLTERKALSIRWLKWQTYFEDPRLIFHYDDTLPDLQNLPYEVVFY
TDGSSIKSPKKDKQHSAGMGIIAVRYPQMNIIQEWSIPLGDHTAQFAEIAAFEFALKQAIRKMGPVLIVTDSDYVAKSYN
QELDFWVSNGFVNNKKPLKHVSKWKSIADCKKHKADIHVIHEPGHQNDLQSPYAMGNNAADKLAVKASYTVFSVPSLDAE
LHQLLDKPNPKGYPSKYEYTLRDGQVYVKRTDGEKIIPSKDDRVKILELAHKSGHLGKNTMYIKILNKYWWPNLIKDISK
YIRTCTNCIITNTDNVPNKSYIVQEKTGLPFQKYYMDYIGPLPPSDGYYHVLVIVDEGTGYTWLYPTKAQTANATVKALN
HLTGTAIPKVLHSDQGSAFTSATLVAWAKDKGIQMEYSSPYHPQSSGKVERKNSEIKRLLTKLLVGRPTKWYPLIPTVQL
ALNNTTPHQLMYGVDCNLPFQDLSTLDLTREEQLAVLQEIRTALWTPCPGLLVQERVNRPAQLRPKWKKPTPILKVLNPK
TVVIAGPGGQERIVSIDNLK

>FFVfca

PTPQKQYHINPKAKPDIQIVINDLLKQGVLIQKESTMNTPVYPVPKPNGRWRMVLDYRAVNKVTPLIAVQNQHSYGILGS
LFKGRYKTTIDLSNGFWAHPIVPEDYWITAFTWQGKQYCWTVLPQGFLNSPGLFTGDVVDLLQGIPNVEVYVDDVYISHD
SEKEHLEYLDILFNRLKEAGYISLKKSNIANSIVDFLGFQITNEGRGLTDTFKTAPTTLKQLQSILGLLNFARNFIPDFT
ELIAPLYWQIEHSLETLITKLNGAEYLQGRKGDKTLIMKVNASYTTGYIRYYNEGEKKPISYVSIVFSKTELKFTELEKL
LTTVHKGLLKALDLSMGQNIHVYSPIVSMQNIQKTPQTAKKALASRWLSWLSYLEDPRIRFFYDPQMPALKDLPFQHIFY

TDGSAITSPTKEGHLNAGMGIVYFINGNLQKQQEWSISLGNHTAQFAEIAAFEFALKKCLPLGGNILVVTDSNYVAKAYN
EELDVWASNGFVNNRKPLKHISKWKSVADLKRLRPDVVVTHEPGHQKLDSSPHAYGNNLADQLATQASFKVHMTPKLDIE
QIKAIQARLPVGYPKQYTYELQNNKCMVLRKDGWREIPPSRERYKLIKEAHNISHAGREAVLLKIQENYWWPKMKKDISS
FLSTCNVCKMVNPLNLKPISPQAIVHPTKPFDKFYMDYIGPLPPSEGYVHVLVVVDAATGFTWLYPTKAQTSKATIKVLN
HLTGLAIPKVLHSDQGSAFTSEEFAQWAKERNIQLEFSTPYHPQSSGKVERKNSEIKKLLTKLLVGRPLKWYNLISSVQL
ALNNTTPHQLMFGIDCNLPFANKDTLDWTREEELALLQEIRESLWSPYVGQLVQERVYRPSQLRPKWRKPTKVLEILNPR
TVIIVDHLGQRKSVSIDNLK

>SFVmcy

PRPQKQYPINPKAKPSIQIVIDDLLKQGVLIQQNSTMNTPVYPVPKPDGKWRMVLDYREVNKTIPLIAAQNQHSAGILSS
IYRGKYKTTLDLTNGFWAHPITPESYWLTAFTWQGKQYCWTRLPQGFLNSPALFTADVVDLLKEIPNVQAYVDDIYISHD
DPQEHLEQLEKIFSILLNAGYVSLKKSEIAQREVEFLGFNITKEGRGLTDTFKTPPKDLKQLQSILGLLNFARNFIPNYS
ELVKPLYWTEDNSLQHIISVLNQADNLEERNPETRLIIKVNSSPSAGYIRYYNEGSKRPIMYVNYIFSKAEAKFTQTEKL
LTTMHKGLIKAMDLAMGQEILVYSPIVSMTKIQRTPLPERKALPVRWITWMTYLEDPRIQFHYDKSLPELQQIPFAMVFY
TDGSAIKHPDVNKSHSAGMGIAQVQFPEYKIVHQWSIPLGDHTAQLAEIAAVEFACKKALKISGPVLIVTDSFYVAESAN
KELPYWKSNGFLNNKKPLRHVSKWKSIAECLQLKPDIIIMHEKGHQQPMTTLHTEGNNLADKLATQGSYVVHCNPSLDAE
LDQLLQGHYPPGYPKQYKYTLEENKLIVERPNGIRIVPPKADREKIISTAHNIAHTGRDATFLKVSSKYWWPNLRKDVVK
SIRQCKQCLVTNATNLTSPPILRPVKPLKPFDKFYIDYIGPLPPSNGYLHVLVVVDSMTGFVWLYPTKAPSTSATVKALN

MLTSIAIPKVLHSDQGAAFTSSTFADWAKEKGIQLEFSTPYHPQSSGKVERKNSDIKRLLTKLLIGRPAKWYDLLPVVQL

ALNNSTPHQLLFGVDSNTPFANSDTLDLSREEELSLLQEIRSSLWSPSVGQLVQERVARPASLRPRWHKPTAILEVVNPR

TVIILDHLGNRRTVSVDNLK

>SFVcae

PRPQKQYPINPKAKASIQTVINDLLKQGVLIQQNSIMNTPVYPVPKPDGKWRMVLDYREVNKTIPLIAAQNQHSAGILSS

IFRGKYKTTLDLSNGFWAHSITPESYWLTAFTWLGQQYCWTRLPQGFLNSPALFTADVVDLLKEVPNVQVYVDDIYISHD

DPREHLEQLEKVFSLLLNAGYVSLKKSEIAQHEVEFLGFNITKEGRGLTETFKTPPRDLKQLQSILGLLNFARNFIPNFS

ELVKPLYWTTDNSLQNIISMLNSAENLEERNPEVRLIMKVNTSPSAGYIRFYNEFAKRPIMYLNYVYTKAEVKFTNTEKL

LTTIHKGLIKALDLGMGQEILVYSPIVSMTKIQKTPLPERKALPIRWITWMSYLEDPRIQFHYDKTLPELQQVPFSMVFY

TDGSAIKHPNVNKSHNAGMGIAQVQFPEFTVINTWSIPLGDHTAQLAEVAAVEFACKKALKIDGPVLIVTDSFYVAESVN

KELPYWQSNGFFNNKKPLKHVSKWKSIADCIQLKPDIIIIHEKGHQPTASTFHTEGNNLADKLATQGSYVVNINPSLDAE

LDQLLQGQYPKGFPKHYQYQLENGQVMVTRPNGKRIIPPKSDRPQIILQAHNIAHTGRDSTFLKVSSKYWWPNLRKDVVK

VIRQCKQCLVTNAATLAAPPILRPERPVKPFDKFFIDYIGPLPPSNGYLHVLVVVDSMTGFVWLYPTKAPSTSATVKALN

MLTSIAVPKVIHSDQGAAFTSATFADWAKNKGIQLEFSTPYHPQSSGKVERKNSDIKRLLTKLLVGRPAKWYDLLPVVQL

ALNNSTPHQLLFGIDSNTPFANSDTLDLSREEELSLLQEIRSSLWSPSVGQLVQERVARPASLRPRWHKPTPVLEVINPR

AVVILDHLGNRRTVSVDNLK

>SFVpve

PRPQKQYPINPKAKPSIQIVIDDLLKQGVLTPQNSTMNTPVYPVPKPDGRWRMVLDYREVNKTIPLTAAQNQHSAGILAT

IVRQKYKTTLDLANGFWAHPITPDSYWLTAFTWQGKQYCWTRLPQGFLNSPALFTADAVDLLKEVPNVQVYVDDIYLSHD

NPHEHIQQLEKVFQILLQAGYVSLKKSEIGQRTVEFLGFNITKEGRGLTDTFKTPPKDLKQLQSILGLLNFARNFIPNFA

ELVQTLYWTEDNTLNKVIEALNTASNLEERLPDQRLVIKVNTSPSAGYVRYYNESGKKPIMYLNYVFSKAELKFSMLEKL

LTTMHKALIKAMDLAMGQEILVYSPIVSMTKIQKTPLPERKALPIRWITWMTYLEDPRIQFHYDKTLPELKHIPYEGVFC

TDGSAIKSPDPTKSNNAGMGIVHAIYPEYKILNQWSIPLGHHTAQMAEIAAVEFACKKALKVPGPVLVITDSFYVAESAN

KELPYWKSNGFVNNKEPLKHISKWKSIAECLSIKPDITIQHEKGHQPINTSIHTEGNALADKLATQGSYVVNCNPNLDAE

LDQLLQGNNVKGYPKQYTYYLEDGKVKVSRPEGVKIIPPQSDRQKIVLQAHNLAHTGREATLLKIANLYWWPNMRKDVVK

QLGRCKQCLITNASNKTSGPILRPDRPQKPFDKFFIDYIGPLPPSQGYLYVLVIVDGMTGFTWLYPTKAPSTSATVKSLN

VLTSIAIPKVIHSDQGAAFTSSTFAEWAKERGIHLEFSTPYHPQSSGKVERKNSDIKRLLTKLLVGRPTKWYDLLPVVQL

ALNNTTPHQLLFGIDSNTPFANQDTLDLTREEELSLLQEIRASLWSPVVGQLVQERVARPASLRPRWHKPSTVLEVLNPR

TVVILDHLGNNRTVSIDNLK

>SFVggo

PRPQKQYPINPKARSSIQVVIDDLLKQGVLVQQNSTMNTPVYPIPKPDGRWGMVLDYREVNKTIPLIAAQNQHSAGILAT

IVRKKYKTTLVLANGFWAHPITPESYWLTAFIWQGKQYCWTRLPQGFLNSPALFTADVVDLLKEISNVQAYVDDIYLSHD

DPQEHLDQLEKVFQILLQAGYVSLKKSEVAQKTVEFLGFNITKEGRGLTEAFKTPPKDLKQLQSILGLLNFARNFILNFA

ELVKPLYWSNENTLQTIIKALNNADNLEERIPEKRLIIKVNTSPSAGYVRYYNETGKKPIMYLNYVFSKAELKFTLLEKL

LTTMHKALIKAMDLAMGQEILVYSPVVSMTKIQKTPIPERKALPIRWITWMTYLEDPRIQFHYDKTLPELKNIPYNSVFY

TDGSAIRSPDPTKSHNAGMGIVQVKFPELQVINQWSIPLGNHTAQMAEIAAVEFACKKALKITGPVLIITDSFYVAESTN

KELPYWKSNGFVNNKKPLKHVSKWKSIAECLSLKPDITIQHERGHQPIYTSIHTEGNALADKLATQGSYVVNNNPNLDAE

LDHLIQGKYPKGYPKQYTYYMEDGKVKVNRPEGTKIIPPSLERAGIVQKAHNLAHTGREATLLKIANLYWWPNMRKDVVR

QLGRCQQCLVTNAFNQTSGPILRPTRPLKPFDKFFIDYIGPLPPSNGYLHVLVVVDSMTGFTWLYPTKAPTTNATVKALN

VLTSIAVPKVIHSDQGAAFTSSTFADWAKERGIQLEFSTPYHPQSSGKVERKNSDIKRLLTKLLVGRPTKWYDLLPVVQL

ALNNSTPHQLLFGIDSNTPFANQDTLDLTREEELSLLQEIRSSLWSPIGGQIVQERVPRPASLRPRWHKPSRIVDILNER

TVVIVDHLGNNRTVSIDNLK

>SFVppy

PRPQKQYPINPKAKESIQIVINDLLKQGVLIQQNSIMNTPVYPVPKPDGRWRMVLDYREVNKTIPLIAAQNQHSAGILAS

IYRGTYKTTLDLANGFWAHPITPNSYWLTAFTWQGKQHCWTRLPQGFLNSPALFTADVVDLMKHIPNVQVYVDDLYLSHD

DPQEHLQVLQQVLHILHDAGYVSLKKSAIAQKVVEFLGFNITKTGRGLTDAFKSPPQNLKQLQSILGLMNFARNFIPNYA

ERVKPFYWNDELTLQELITLLNQADNLEERKPTTRLIIKVNSSSHAGYIRYYNEGSKKPILYINYVFSKAEEKFSMLEKL

LTTLHKALIKAVDLAMGTEIMVYSPIVSMTKIQKTPLPERKALPVRWITWMTYLEDPRITFHYDKTLPELKDVPYSMVFY

TDGSAIKNPNPTKTHSAGMGVVQGKFPEFQVVNQWSIPLGNHTAQLAEVAAVEFACKQALKITGPVLIITDSFYVAESAN

KELPYWKSNGFVNNKKPLKHVSKWKSIADCLSLKTGITIKHEKGHQPSHTSVHTEGNALADKLATQGSYVVNNIPSLDAE

LDQVLQGNLPKGYPKHFVYTLEEGKVIVKRPEGTKIIPPLADRKLLASQAHKLSHSGREATLLKLSNTYWWPNMRKDVVK

VIGQCQQCLVTNPSNLTSGPILRPERPTKPFDKFFIDYIGPLPPSNGYLHVLVVVDAMTGFVWLYPTKAPSANATVKALN
MLTSIAVPKVIHSDQGAAFTSSTFADWAKEKGIHLEYSTPYHPQSSGKVERKNSDIKRLLTKLLVGRPTKWYDLLSTVQL
ALNNATPHQLLFGVDANIPFANQDTLDLTREEELSLLQEIRESLWTPAVGLLVQERVARPASLRPRWHKPVKILEVLNPR
TVVILDHLGNNRTVSVDNLK

>SFVcja

PRPQRQYHINTKAKPSIQQVIDDLLKQGVLIKQTSVMNTPIYPVPKPDGKWRMVLDYRAVNKTVPLIGAQNQHSLGILTN
LVRQKYKSTIDLSNGFWAHPITKDSQWITAFTWEGKQHVWTRLPQGFLNSPALFTADVVDLLKNIPGISVYVDDIYFSTE
TVSEHLKILEKVFKILLEAGYVSLKKSALLRYEVTFLGFSITQTGRGLTSEFKTSPRTLKELQSILGLFNFARNFVPNFS
EIIKPLYWTSEHTLEEIVSALNHAGNLEQRDNESPLVVKLNASPKTGYIRYYNKGGQKPIAYASHVFTNTELKFTPLEKL
LVTMHKALIKAIDLALGQPIEVYSPIISMQKLQKTPLPERKALSTRWITWLSYLEDPRITFYYDKTLPDLKNVPYAAVFY
TDGSAIRSPDKNKSHSSGMGIVHAVFPELTIEHQWSIPLGDHTAQYAEISAVEFACKKANNISGPVLIVTDSDYVARSVN
EELPFWRSNGFVNNKKPLKHISKWKNISDSLLLKRDIIIVHEPGHKPSYTSIHTQGNNLADKLATQGSYTVNNIPSLDAE
LEQLINGHSVKGYPSRYKYILKEGQVFVLRPEGEKIIPPKSDRLALVKIAHEFSHAGREATVLRLQDKYWWPNMRKDVIS
HIRTCKPCLTTDGSNLTPIPPKPQLRPEKPFDKFFIDYIGPLPPSHGFVYVLVVVDAATGFTWLYPTKAPSTNATITSLN
ILLGTAVPRVLHSDQGSAFTSSTFADWAKEKGIQLEFSTPYHPQSSGMVERKNREIKRLITKLLVGRPTKWYPLLPTIQL
ALNNTTPHQLLFGVDGNVPFANQDTLDLTREEELSLLSEVRTSLWLPSVGLLVQERVARPSQLRPKWKKPTPILEVVNDR
TVVILDNQGQRRTVSIDNLK

>SFVssc

PKPQKQYHINPKAKPSIQIVINDLLKQGVLKQQNSIMNTPIYPVPKTEGKWRMVLDYRAVNKTIPLIAAQNQHSAGILTN

LVRQKYKSTIDLSNGFWAHPIDQDSQWITAFTWEGKQYVWTRLPQGFLNSPALFTADVVDLLKEIPNVNVYVDDIYVSTE

TINQHFQVLDKIFQKLLQAGYVSLKKSNLCRYEVTFLGFTISKYGRGLTEEFQSPPNSLKQLQSILGLLNFARNFIPNFS

ELIKPLYWEPKHSLNNLIIALNHADNLEQRNGEVPLVIKINASNTTGYIRFYNKNGKRPIAYASHVFNHTEQKFTPVEKL

LTTMHKAIIKGIDLAIGQPIEIYSPIVSMQKLQKITLPERKALSTRWLSWLSYIEDPRFLFIYDKTLPDLKEMPYLAVFY

TDGSSIKSPDPTKTHSSGMGIVQAIYPNFQIKHQWSIPLGDHTAQYAEIAAVEFACKKALQVTGPVLIVTDSDYVARSVN

NELNFWRSNGFVNNKKPLKHISKWKSISESLLLHKNITIVHEPGHQPSSTSVHTQGNALADKLAVQGSYTINNIPSLDTE

LRAVLEGKLPKGYPKNLKYEYNSPNLIVIRKEGQRIIPPLSDRPKLVKQAHELAHTGREATLLRLQNQYWWPKMRKDVSH

CLRTCMPCLQTNSTNLTTTRPFQQIRPSKPFDKYYIDYIGPLPPSEGYSYVLVVVDSATGFCWLYPTKAPSTRATVKSLN

FLLGIAVPKILHSDQGSAFTSSDFANWAKEKEITLEFSTPYHPQSSGKVERKNQEIKKLLTKLLVGRPAKWYPLIPSVQL

ALNNTTPHQLLFGVDGNIPFANSDTLDLKREEELALLSEIRTTLWTPSVGLLVQERVYRPSQLRPKWKKPTPILEVLNER

TVVI-DNNGQRRTVSVDNLK

>SFVocr

------------------------------------MNTPVYPVPKPDGKWRMVLDYRAVNKTVPAIGAQNCHAPGILSS

LYRAKFKTTLDLSNGFWSHPITPESYWLTAFTWQGSQYVWTRLPQGFLNSPALFTADVVDLCKHIPNVSAYVDDIYVSND

TAEEHLRTLEQLFRTLMSAGYVSLKKSKIGVSAVDFLGFEITDDGRGLTSAFKQPPSSLKQLQSILGFLNFTRNFVPNYS

ELVKPLYWTLENTLNQVISALNAADNLSERKTGVPLVVKSNCSPTAGYIRFYNQGDRKPIQYVNYIFSKTELKFTPLEKQ
LTVLHKAILKGLDLAGGEDIHFYTPIASISKLQRTPIPERKALHVRWLTWITYLEDPRFHFYYDETLPPLAELPYTSVFY
TDGSAIKNPNPKKAHSAGMGTVEVTYPEYKVLHEWSFPLGDHTAQYAEIAACEFAIKKASLLRGPVLIVSDSVYLVKSFN
EELPFWISNGFLNNKKPLQHISKWKTIAACYQNKKDIFLLHVPGHQKLLTDEHAQGNALADKLAVQSSHKVLFIPSLDAE
LIQVMEGKYPKGYPHKYVYAQDNGKIIVTLPNGQREIPPVGDRLALITKAHNISHMGREAVLAKIQNVYWWPNMKKDVKH
VLTICSQCQQVNSFNLKPQPPQTIARHVHPFDKIYMDYIGPLPPSDGYLYVLVLVDSCTGFTWLYPTKAPSANATVKALT
HLTGTAVPKVLHSDQGSAFTSSTLVDWAKERGIRLEYSTPYHPQSSGKVERKNSEIKRLLTKLLVGRPLRWYPLIPTVQL
ALNNTTPHQLLFGVDCNLPFANKDTLDLTREKQLSLLKELREDLWRPSVRLLVQERVYRPSALRPKWRKPTPILEVHSDR
LVTIKDHLGNIKKVSTDNLK
>TraEFVtja.a1
SRPQRQYHINPKAKPDMQIVVNDLLKQGVLIQKESSMNTPVYLVPKSNGRWRMVLDYREVNKVTPLIATQNQHSAGILSS
LYRGKYKSTIDLANGFWAHPITRESYWLTAFAWSGKQYCWMVLPQDFI----ALLCSLRMLMYEITNVQVYVDDVYLSND
TIEEHLDALRKMFQLLREAGYVSLQKSALCQSTIEFLGFQITSTGRGWTSSFTKPPTTLKDLQSILGFMNFARNFLPNFA
ELIAPLYWTSEHTLTKLIKALNDAAELQERDPTLKLILKCNACPTTGYARFYNEKGISPIQYLSILF--TELKFSILEKL
LTTIHKALLKGLDLAMGQGIMVYSPVVSMTKIQKTLITARKALASRWVTWMTYLEDPRIEFYYDNTLP-LKLLPYASEFY
TDGSAIKSPDKTKTYCSGMGIVQYKYPEPKIIKEWSVPLGDHTAQFAEIAT-EFACKQALKIQGPILIMTDSDYLAKSYN
NELSYWISNRFVNNRKPLTHIGKWKNIADCLKIKPDIFVAHEPGHQETNVSLHAKVNGLADTLAIHASYTVNTVPELDSE

LNQLLSGITIKGSPTQFKYELQGMDVVTIRPEGIRTVPPRSNRAKIIKEA--LSLGGQNTTMMTLQQKFWWPNMRKDVVK

YLRLCEICAPVNPSNIKPSRPILQTRPPKPFERFQMDLIGPLPTSEGYSSVLVIVDVASGYIWLYPTKAQTTKGTIKALN

LLTGTAIPKVLHSDQGAAFISSDLATWAKEKNITLEFSTPYHPQSSGKVERENSEIKRLLTKLLVGWPMKWYNLIPDTQF

ALNNTIPHQLMFGIGCNQPFANRDTFNWTREEQLALLAEIRQSLWLPTVGQLIQESVYRPSQLRPKWRKPTPIVAVLNNH

TVQIVDHLGQPKIVSVDNLK

>TraEFVtka.a2

SRPQRQYHINPKAKPDMQIVVNDLLKQGVLIQKESSMNTPVYLVPKSNGRWRMVLDYREVNKVTPLIATQNQHSAGILSS

LYRGKYKSTIDLANGFWAHPITRESYWLTAFAWSGKQYCWMVLPQDFI----ALLCSLRMLMYEITNVQVYVDDVYLSND

TIEEHLDALRKMFQLLREAGYVSLQKSALCQSTIEFLGFQITSTGRGWTSSFTKPPTTLKELQSILGFMNFARNFLPNFA

ELIAPLYWTSEHTLTKLIKALNDAAELQERDPTLKLILKCNACPTTGYARFYNEKGISPIQYLSILF--TELKFSILEKL

LTTIHKALLKGLDLAMGQGIMVYSPVVSMTKIQKTLITARKALASRWVTWMTYLEDPRIEFYYDNTLP-LKLLPYASEFY

TDGSAIKSPDKTKTYCSGMGIVQYKYPEPKIIKEWSVPLGDHTAQFAEIAT-EFACKQALKIQGPILIMTDSDYLAKSYN

NELSYWISNRFVNNRKPLTHIGKWKNIADCLKIKPDIFVAHEPGHQETNVSLHAKVNGLADTLAIHASYTV---PELDSE

LNQLLSGITIKGSPTQFKYELQGMDVVTIRPEGIRTVPPRSNRAKIIKEA--FSLGGQNTTMMTLQQKFWWPNMRKDVVK

YLRLCEICAPVNPSNIKPSRPILQTRPPKPFERFQMDFIGPLPTSEGYSSVLVIVDVASGYIWLYPTKAQTTKGTIKALN

LLTGTAIPKVLHSDQGAAFISSNLATWAKEKNITLEFSTPYHPQSSGKVERKNSEIKRLLTKLLVGWPMKWYNLIPDIQF

ALNNTIPHQLMFGIGCNQPFANRDTFNWTREEQLALLAEIRQSLWLPTVGQLIQESVYRPSQLRPKWRKPTPIVAVLNNH

TVQIVDHLGQPKIVSVDNLK

>TraEFVtja.a2

PRPQKQYHINPKAKPDIQIIVNDLLKQGILIQKESSMNTPIYPVPKNSGKWRMVLDYREVNKVTPLIATQNQHSAGILSS

LYRGKYKSTIDLANGFWAHPITRESYWLTAFTWSGKQYCWTVLPQGFLNSPALFTGDVVDLLKEITNVQIYVDDVYLRHD

TFKEHLDALRKMLQLLREAGYVSLQKCALCQSTVEFLGFQITNTRRGLMSSFEKSPTTLKTLQSILGFMNFARNVLPNCA

-LIAPLYWTSEHTLTELINALNDATELQERDPTLKLIIKCNACPTTGYARFYNEKGISPIQYLSVLFTKTELKFSNLEKL

LTTIHKALLKVLDLAMRQGIMVYSPVVSMTKIQKTPITAKKNTS------FVFL--------------------------

----------------------------------WEIIL--HS---AETAALEFACKQALKIQGPILITTDSDYLAKSFN

NELSYWISNGFVNNRKPLTHIGKWKNIADCLKIKPDMFMAHESGHQKTNASLHAKGNSLADTLAVHASYTVNTVPELDSE

LNQLLSGITIKGYPTKFKYELQGIDVVTVQPEGIRIVPPRSNRDKINKEAHALSHGGRNTTMMTLQQKYWWQNMRKDIVK

HIRLCDICAQVNPNNIKPSRPIIQNRPPKPFERFQMDFIGPLLPSEGYTSVLVIVDVATGYTWLYPTKTQTTKATVKALN

LLTGTAIPKVLHSDQGAAFTSSDLATWAKEKSITLEFSTPYHPQSSGKV-RKNSEIKRLLTKLLVGQPMKWYSLIPDIQF

ALNNTTPHQLMFGIDCNQPFANQDTFHWTREEQLALLAEIRQSLWILTVGQLVQERIYRPSQL-PKWRKPTPIVAVLNNR

TVQIVDHLGQLKTVSVDNLK

>TraEFVtka.a1

PRPQKQYHINPKAKPDIQIIVNDLLKQGILIQKESSMNTPIYPVPKNSGKWRMVLDYREVNKVTPLIATQNQHSAGILSS

LYRGKYKSTIDLANGFWAHPITRESYWLTAFTWSGKQYCWTVLPQGFLNSPALFTGDVVDLLKEITNVQIYVDDVYLRHD

TFKEHLDALRKMLQLLREAGYVSLQKCALCQSTVEFLGFQITNTRRGLMSSFEKSPTTLKTLQSILGFMNFARNVLPNCA
-LIAPLYWTSEHTLTELINALNDAAELQERAPTLKLIIKCNACPTTGYARFYNEKGISPIQYLSVLFTKTELKFSNLEKL
LTTIHKALLKVPDLAMRQGIMVYSPVVSMTKIQKTPITAKKALASRWVT-MAYLEDPRIEFYYDNTLPELKLLPYASIFY
TDGSAIKSPNKTKTYCSGMGIVQYKYPKPKVIKEWSVPLGDHTAQ-AETAALEFACKQALKIQGPILITTDSDYLAKSFN
NELSYWISNGFVNNRKPLTHIGKWKNIADCLKIKPDMFMAHESGHQKTNASLHAKGNSLADTLAVHASYTVNTVPELDSE
LNQLLSGITIKGYPTKFKYELQGIDVVTVQPEGIRIVPPRSNRDKINKEAHALSHGGRNTTMMTLQQKYWWQNMRKDIVK
HIRLCDICAQVNPNNIKPSRPIIQNRPPKPFERFQMDFIGPLLPSEGYTSVLVIVDVATGYTWLYPTKTQTTKATVKALN
LLTGTAIPKVLHSDQGAAFTSSDLATWAKEKSITLEFSTPYHPQSSGKV-RKNSEIKRLLTKLLVGQPMKWYSLIPDIQF
ALNNTTPHQLMFGIDCNQPFANQDTFHWTREEQLALLAEIRQSLWILTVGQLVQERIYRPSQL-PKWRKPTPIVAVLNNR
TVQIVDHLGQLKTVSVDNLK
>TraEFVtja.a4
PQPQKQYHVNPKAKPDIQIVINDSLKQGLLVQKESSLNTPIYPVPESNGKWRMVLDYREVNKVTPLIATQNQHSAGILSS
LYRGKYKSTIDLANGFWAHPITRESYWLTAFTWSGKQYCWTVLPQGFLNSPALFTGDVVDLLKEITNVQVYVDDVYLSHD
TFEEHLDSLRKMFQLLREAGYVSLHKSALCQSIVEFLGFQITSTGRGLTSSFQKPPNTLKELQSILGFMNFARNFLPNFA
ELVAPLYWTSEHTLTKLINALNEAAELQERDPTLRLIIKCNASPTTGYARFYNEKAVSPIQYLSILFTKTELKFSPLEKL
LTTIHKALLKGLDLAMGQGIMVYSPVVSMTKIQKTPITERKALASRWVTWMSYLEDSRIEFYYDNTLPELKLLPYASILY
SDGFAVRSPDKTKVHCSEMGIVQYKYPEPIIIKEWSIPLGDHTAQFAEIAAFEFAFKQALKILGHILIITDSDYLAKSFN

NESKYWILNGFVNNRKPLTHIGKWKNIADCLKIKPDIFVAHEPSHQKPNVSLYAKGNSLADKLAVHASYTVNTVPELDSE

LNQMLPGITIKGYPTKFKYELQGTDVITTQPEEIRIVPPKSNRDKIIKEAHTLSHGGHNTTMMTLQQKYWWPNMKKDVVR

QLHLCNVCTQVNLSNIKPSVPLIQAHPPKPFERFQMDFIGPLSLSEGYTSVLVIVDVATGYVWLYPTKAQTTKATIKALN

LLTGPAIPKVLHSDQGAAFISSNLATWAKEKNITLEFSTPYHPQSSGNVERKNSEIKRLLTKLLVGRPMKWYSLIPDIQF

ALNNTTPHQLMFGIDCNQPFANQDTFDWTHEEQLALLTEIRQSLWIPTVGQLVQEKVYRPSQLHPKWRKPTPIVAVLNNH

TVQIVDHLGQPKTVNADNLK

>TraEFVtka.a4

PRPQKQYHVNPKAKPDIQIVINDLLKQGVLVQTQSSMNTPIYPVPKSNGKW-MVLDYREVNKVTPLIATQKQHSAGVLSS

LYRGKYKSAIDLANGFWAHPITRESYWLTAFTWSGKQYCWTVLPQGF-NSPAVFTGDVVDLLK-IATVQVYVDDVYLRHD

TLKEHLDSLRKMFQLLR-VGYVSLHKSALCQSIVEFLGFQITSTGRGLTSSFKKPPNTLKKLQSILGFMNFARNFLPNFA

ELVAPLYWTSELTLTKLNNALNEEAELQERDPTLKLIIKCNASPTTGYARFYNEKGVSPIQYLTILFTKTELKFSPLEKL

LTTIHKVLLEGLDLAMGQGIMVYSPVVSMTKIQKTPIISRKALASRWVTWMSYLEDSRIEFYYDNTLPELKLLPYASILY

SDGFAVRSPDKTKVHCSEMGIVQYKYPEPIIIKEWSIPLGDHTAQFAEIAAFEFAFKQALKILGHILIITDSDYLAKSFN

NESKYWILNGFVNNRKPLTHIGKWKNIADCLKIKPDIFVAHEPSHQKPNVSLYAKGNSLADKF-----------------

--------------------------------------------------------------------------------

--------------------------------------------------------------------------------

--------------------------------------------------------------------------------

--------------------------------------------------------------------------------

--------------------

>TraEFVtja.b1

SQASKQYHINSKAKPDIQIVINHLTKQGVLIQQESTMNTPVYPIPEGNGKWRMVLDYREVNKVTPVITSQNYHSKGILAS

IYRGKYKSTIDLANGFGAHPIAKESYWITAFTWAGEQYCWTVLPQGFLNSPALFTGDIVDLLKEVNNVQVYVDDVYLSHD

MFEEHYNALDQMLMILKEAGYVSLCKSQLF-KRVEFLGFQITNKGRGLTD-FIDTSNDPETITEIWGFLNFARIFLPNFS

ELITPLCWTKEHTLDRIVTDLNMGAELVEQKPK-----RGNAMPLLQLVILDFTMMEEILQYMSIIFTKTESKFTPLEKL

LAVIHKGLLKALNLALGQDIRIYSPVVSMQKIPKTSITARKALASRWVTWMTYLEDPRTEFYYDNTLPELKQLPYTAVFY

TDGSVVKFPKEGKILSAGMGVVLCNFPEYKLIKQWSTPLEDHTAQFAEITAMEFAFKQALKIQGPVLVVTDSDYLAKSFN

NELSYWISNGFLNNRKPLIHIGKWKCIAECLKIKPDISVGREPSHQKLNTSSHTIGNSLADKLAVQASYKVDVIPELDSE

LNQLLLGTFPKGCPSKYIYELKDGQITAARPEGNRIIPPICARTQIILQAHQVAHSGHVILLLKLKNKYWWPNMRRDVIK

TLATCETCHRENKPTSAPQMPVSQERLDKPFEKFQMDYIGPLPPSEGYLYILVIADMATGYLWLYPTKAQTAKATIKALN

LLTGTVIHRVLHSDQGSAISSFDLATWAKDKGIQLEFNTSYHPQSSGKVGGKDSEIKKLLTKLLAGSPIKWYNYISDIQF

ALNNITPHQLMYGVDCNQPFANQDTFDWTREEQLAILKEICESLWTPEVGLLVQERVYKPSQLS-KWQKLTPIVTVLNN-

TVQIVDHLG-----------

>TraEFVtja.b6

---------------------------------------------------------REVNKVTPIIATPNCHSTGILAS

MYREKYKSTIDLANGFGAHPIARESYCTTAFTWAGKQYCWTVLPQGFLNSPALFTGDIVDLLKEINNVQVYVDDVYLSHD

TFEEHYNALGQMLMILKEAGYVSLHKSQLFQKQVEFLGFQVTNKGRGLTVSYK--------------------------

------------------------------------------------------------------------------

------------------------------------------------------------------------HTAVFY

TDGSAMKSPKEGKIHPAGMGVVLCNFPEYKLIKQWSTPLGDHMAQFTKITVMELAFTQALKIQGPVLVVTNSDHLAKSFN

NELSYWISNGFLNNRKPLIHIGKWKCIAECLKIKPDILVVHEPSHQKPKTSSHTMGNYLAGKLAVQVSYKVDVI--LDSE

LNQLLLRTFPKGYPSKYIYKLKDGQVIVARPEGNRIIPPVSARTQIILQAHQAAHSGSEISLLKLKSKYRWPNMRQDVVK

TLATCETCHRVNKPTSRHKYQSHRGRPDKPFEKFQMDDIGPLPPSEGYLHILVIVDTATGYLWLYSTKAQTAKATIKALN

LLTGTVIPRMLHSDQGSAFTSFDVATWAKDKGMQMEFSTPYHPQSSG-VERKNSEIKSF--------------------

------------------------------------------------------------------------------

-------------------

>TraEFVtka.b4

--------------------------------------------------------REVNKVTPIIATPNCHSTGISAS

MYRGKYKSTIDLANGFGAHPIARESYCTTAFTWAGKQYCWTVLPQGFINSPALFTGDIVDLLKEINNVQVYVDDVYLSHD

TFEEHYNALGQMLMILKEAGYVSLHKSQLFQKQVEFLGFQVTNKGRGLTVSYK--------------------------

------------------------------------------------------------------------------

---------------------------------------------------------------------------FFY

TDGSAMKSPKKGKIHPAGMGVVLCNFPEYKLIKQWSTPLGDHMAQFTKITVMELAFTQALKIQGPVLVVTNSDHLAKSFN

NELSYWISNGFLNNRKPLIHIGKWKCIAECLKIKPDILVVHEPSHQKPKTSSHTMGNYLAGKLAVQVSYKVDVI-QLDSE

LNQLLLRTFPKGYPSKYIYKLKDGQVIVARPEGNRIIPPVSARTQIILQAHQAAHSGSEISLLKLKSKYRWPNMRQDVVK

TLATCETCHRVNKPTLRHKYQSHRGRPDKPFEKFQMDDIGPLPPSEGYLHILVIVDTATGYLWLYSTKAQTAKATIKALN

LLTGTVIPRMLHSDQGSAFTSFDVATWAKDKGMQMEFSTPYHSQSSG-VERKNSEIKSF---------------------

-------HQLMYGVDCNQLLTGLEKNNWPSSKKFVNLLLILLAL--QKLACWSRKGCIGPHNLSGRNRHPL-FVTVLNNP

TVQIVDHLGRLKTISVDNLK

>TraEFVtja.b7

PRSQKKYHINPKAKPDIQIVINDLIRQGVLI-QDSTLNTPVYPVPKGNRK-RMILDYQKINEVTPIIATPNCHSTRILAS

IYREKYKSTIDLANVFSPHPIARESYWITAFTRE-KQYYWTVLPQGFLNSPALFTGNIVDLLKEINNVQVYVDDMYLSHD

MFEEHYNVLDQMLMILKETGYVSLHKYQLFLKQVKFLGFQITNKRQGLTSSYKTCPTTLKQLQSILGFLNFARNFLPNFS

KFITPLYWTKEHTLDKIVTDLNTAAELVEQKPKNKLIMKCNASPTTGYIRFYNNRENSPIQYVSIIFTKTESKFTPLEKL

F--------------------------------------------------------------------------------

-------------------------------------------------------------------FVVTDSDYLTKSFN

SELSYWISNGFLNNKKPLVHTRK-KCIDKCLKIKPAISVVHEPSHQNLNTSSHTMDNSLTDKLTVQASYKLDVIPKLGSE

LNQLL----PKGCPSKYIYKLKDGHVIVARPEGNRIIPPVSACTQIILQAHQAAHSGREISLLKAKKQFYWPNMRQNVVK

TLATCKKCHRVNKPMSAPQMPISQRQPDKPFEKFQMDYIGPLSPSEGYLYILFIVDIATGYLWLYPTKAQTAKTPIKALN

LLTGTVIPRVLHTGQGSTCTSSDLAT-AKDKEIQLEFSTPYHFQNSGKVKGKKQEDETFDT---YGKAYK-VVLYFRYPV

CLNNTTPHQLMYGVDYNQHFASQDTFDWTRKEQLAILKKICESLSTPEVGLLAQERVYRPLQLPPKWRKLTPIVTVLNN-

TVQIVDHLGQLKTISVDNLK

>TraEFVtka.b5

PRSQKKYHINPKAKPDIQIVINDPIRQGVLI-QDSTLNTPVYPVPKGNRK-RMILDYQKINEVTPIIATPNCHSTRILAS

IYREKYKSTIDLANVFSPHPIARESYWITAFTRE-KQYYWTVLPQGFLNSPALFTGNIVDLLKEINNVQVYVDDMYLSHD

MFEEHYNVLDQMLMILKETGYVSLHKYQLFLKQVKFLGFQITNKRQGLTSSYKTCPTTLKQLQSILGFLNFARNFLPNFS

KFITPLYWTKEHTLDKIVTDLNTAAELVEQKPKNKLIMKCNASPTTGYIRFYNNRENSPIQYVSIIFTKTESKFTPLEKL

F----------------------------------------------------------------------------

---------------------------------------------------------------FVVTDSDYLTKSFN

SELSYWISNGFLNNKKPLVHTRK-KCIDKCLKIKPTISVVHEPSHQNLNTSSHTMDNSLTDKLTVQASYKLDVIPKLGSE

LNQLLLGTFPKGCPSKYIYKLKDGHVIVARPEGNRIIPPVSACTQIILQAHQAAHSGREISLLKAKKQFYWPNMRQNVVK

TLATCKKCHRVNKLTSAPQMPISQRKPDKPFEKFQMDYIGPLSPSEGYLYILFIVDIATGYLWLYPTKAQTAKIPIKALN

LLTGTVIPRVLHTGQGSTCTSSDLAT-AKDKEIQLEFSTPYHFQNSGKVKGKKQEDETFDT---YGKAYK-VVLYFRYPV

CLNNTTPHQLMYGVDYNQHFASQDTFDWTRKEQLAILKKIYESLSTPEVGLLAQERVYRPLQLPPKWRKLTPIVTGLNN-

TVQIVDHLGQLKMISVDNLK

>TraEFVtja.b3

PRPQRQYHINPKARSDIQIVIDDLIKQGVLIQQENTMNTPVYPVPKGNGKWRMVLDYREVNKVTPLVATQNCYSTGILAS

IYREKYKSVIDLADGFWAYRIMRDSYWITAFTGTIVNYFWTMLPQGFLNSPALFTGDVVNLLKGISNIQVYIDDVYLSPD

TSEEHYDVLNQVFTLLREAGYVSLHKSQFFQKQVEFLGLQITDKGRGLTQSFKTPPANFETITEYFRVFKFCKVFTTFW-

-LITPLY-AQEHTLNKLIANLNAATELNER--------------------------------------PDIKFTPLEKL

LTVMHKGLLKALELAMGQDICVYSPVVSIQKIRKTPITARKALASRWITWMAYLEDPQLKFYYDNTLPELKQLPYVAVFY

TDGSAIKSPKKGKVHSAGMGIAQYVFPEHRLIRQWSTPLGNHTAQFAEIAAIEFAFKQALKVQGPVLIVTDSDYLAKSFN

NGLGYWI-NGFLNNKNPLTHTGKWKSIAECFKLEPDISVVQERSHQRLNTSSHTMDNILADKLATQASYKVNTVPELDSE

LNQLLEGKFLKGSPSKCTYELRDNQIVIIWPGGSRIIPPPSTQPYIISQAHQSAHSGK-ILLLKLKNKY-WPNMRQDVIK

TLAMCKPCHRVNQPSSKRQTPILQGWPDKPFVKFQMDYIGPLPPSEEYLHVLVIVDMATGYVWLYPIKTQTAKATIKALN

LLTGTTIPRVLHSDQRSAFASSNLADWAKDKGIQLEFSTPYHPQSN-----------------LAERPMK-YKYIPDIQF

ALNNTTPHQLMYGVDCNQPFANQDTFDWT-HEQLAILKEIRESVWIPTVGLLVQGRVYRPSQLQPKWQKPTPVAAVLNNK

TVQIVDHLGQLKTV------

>TraEFVtka.b1

PRPQRQYHINPKARSDIQIVIDDLIKQGVLIQQENTMNTPVYPVPKGNGKWRMVLDYREVNKVTPLVATQNCYSTGILAS

IYREKYKSVIDLADGFWAYRIMRDSYWITAFTGTIVNYFWTVLPQGFLNSPALFTGDVVNLLKGISNIQVYIDDVYLSPD

TSEEHYDALNQVFTLLREAGYVSLHKSQFFQKQVEFLGLQITDKGRGLTQSFKTPPANFETITEYFRVFKFCKV-LPHFG

LLYINFL--------QIFINLLIMQPLSMKGSLIKGVMKCNASSTTGYIRFYNDEENSPIQYISIIFTKTEIKFTPLEKL

LTVMHKGLLKALELAMGQDICVYSPVVSIQKIRKTPITARKALASRWITWMAYLEDPQLKFYYDNTLPELKQLPYVAVFY

TDGSAIKSPKKGKVHSAGMGIAQYVFPEHRLIRQWSTPLGNHTAQFAEIAAIEFAFKQALKVQGPVLIVTDSDYLAKSFN

NGLGYWI-NGFLNNKNPLTHTGKWKSIAECFKLEPDISVVQERSHRRLNTSSHTMDNILADKLATQASYKVNTVPELDSE

LNQLLEGKFLKGSPSKCTHELRDNQIVIIWPGGSRIIPPPSTQPYIISQAHQSAHSGK-ILLLKLKNKY-WPNMRQDVVK

TLAMCKPCHRVNQPSSKPQTPILQGWPDKPFVKFQMDYIGPLPPSEEYLHVLVIVDMATGYVWLYPIKTQTAKATIKALN

LLTGTTIPRVLHSDQRSAFASSNLADWAKDKGIQLEFSTPYHPQSNKK---KNSEFN------LAERPMK-YKYIPDIQF

ALNNTTPHQLMYGVDCNQPFANQDSFDWT-HEQLAILKEIRESVWIATVGLLVQGRVYRPSQLQPKWQKPTPVAAVLNNK

ILILADKLATQASYKVNTVK

>TraEFVtka.b3

--------------------------------------------------------------------------------

--------------------------------------------------------------------------------

----------------------------------------------------FLPPITLKQLQSILGFLNFARNFL-NFG

ELITPIYWTQEHTLHKSIANLNAATELNEKKPDKRLIMKCNVSPTTGYIRYYNDEENSPVQYMSTIFTKTELKFTPLEKL

LTVIHKGLLKAFDLAMGQAIHVYSPVVSMQKIQKTPITARKTLASRWVTWMTYLEDPWLEFYYDTTLPELKQLPYAAVFY

TDGSTIKSLKEGKVHSAGTGIAQYVFPEHRLIRQWSTPLGDHTAQFAEIAAIEFAFKQALKVQGLVLIVTDSDYLAKSFN

NELRSWIS-------YKETFDSYWEMEKHCKMLQTQAR--HISGTAKIKYFSHNGSGQIGNTSLIKGCYKI---PRAGSE

LNQLLSGTFPKGYPSKYIYKLKDGQVIVARPEGNRIIPPVPARTQIIL-VHQAAHSGREISLLKLKNKY--PNMRWDVVK

TLAICETCHRVNQSSSKPQMPVVQGRPDKPFVKCQMDYIGPLPPSEGYLHVLVIVDMATGYAWQYPTKTQMAYATSKALN

-LTGTAIPRLLHSDQGSAFTSSDLADWAKDKEIQLEFSTPYHPQSSRKVERKNNEIK-LLTKLLAGIPMKWYKCLPDIQF

ALNNTTPHQLTYAVDCDQPFANQDTFDWTREEQLVILKEIRESLWIPTVGLLVRERVYRPSQL-PKWQKPTPVVAVLNNR

TVQVVDHLGQVKMVNVDNLK

>TraEFVtja.b2

--------------------------------------------------------------------------------

--------------------------------------------------------------------------------

----------------------------------------------------FLPPITLKQLQSILGFLNFARNFL-NFG

ELITPIYWTQEHTLHKSIANLNAATELNEKKPDKRLIMKCNVSPTTGYIRYYNDEENSPVQYMSTIFTKTELKFTPLEKL

LTVIHKGLLKAFDLAMGQAIHVYSPVVSMQKIQKTPITARKTLASRWVTWMTYLEDPWLEFYYDTTLPELKQLPYAAVFY

TDGSTIKSLEEGKVHSAGTGIAQYVFPEHRLIRQWSTPLGDHTAQFAEIAAIEFAFKQALKVQGLVLIVTDSDYLAKSFN

NELRSWIS-------YKETFDSYWEMEKHCKMLQTQAR--HISGTAKIKYFSHNGSGQIGNTSLIKGCYKI---PRAGSE

LNQLLSGTFPKGYPSKYIYKLKDGQVIVARPEGNRIIPPVPARTQIIL-VHQAAHSGREISLLKLKNKY--PNMRWDVVK

TLAICETCHRVNQSSSKPQMPVVQGRPDKPFVKCQMDYIGPLPPSEGYLHVLVIVDMATGYVWQYPTKTQMAYATSKALN

-LTGTAIPRLLHSDQGSAFTSSDLADWAKDKEIQLEFSTPYHPQSSRKVERKNNEIK-LLTKLLAGIPMKWYKCLPDIQF

ALNNTTPHQLTYAVDCDQPFANQDTSDWTREEQLVILKEIRESLWIPTVGLLVRERVYRPSQL-PKWQKPTPVVAVLNNR

TVQVVDHLGQVKMVNVDNLK

>TraEFVtja.b4

PRPQK-YHINPQVKPDIQIVTNDLIRQGALIQQDSTMNTPVYPIPKGNEKWRMVLDYGAVNKVTPIIATQNVTLQGF--P

PYTGENMSAINLADDLRAHSICRESHWITYFTWAGKQYCWTLSPQGFLNSLASFIVDVVDLLKEIKNVQVYVDDVYLSHD

TFEKHYNALDQML--MIKARYVSLHKSHLF-KQVECLGFQITNKGRGLTNSYKPPPMTLKQLESILILLRFSKKILTNFS

ELIAPLYWTKEHTSDRIVTDLNRVTESVKLKREKGLIIKGNATPTTGYIRFCNDGENSPIQYISISFMKPESKFKTLDE-

--------------------------------------------------------------------------------

------------------------------------------------------------------VTVIDNDFWINLFN

NELSFWISSGFLNNRKPPVHI-KGKCVAEFLKIKTDLSVLHEASHQKLNTSSHIMDNTLAYKLAVQALYKVDVIPKLNSE

LNQLL-GTFPKGSPSKYIYERKDGQVIV---EGNKIISPVSASTQIILQAHQTAHSGHEVSLHKLKNKYWLPDMRRDVVK

TIAMCEACHRVNKPTSAPQMPVSQRGPEKPFERFHMDYIGPLPPSEGYLRILVIVDIATGYFSLYPTKAQTDKAPMKFLN

LLTGTVIPRVLHSGQGSALTSSNLVPCGKDKGMQLEFSNPFTSKRVGSGKEKQDEA---FDQTFCWKAYKRHNYISDMQF

VLNNTAPHQLMYGFNCNQPLANQDTFDCTNKK------------------------------------------------

--------------------

>sloEFV

PKPQKQYHINYKAKLAIQTVINDLIKQGVLLHQNSSMNTPIYPVPKTNGSWRMVLNFRAVNKVIPLIAVQNQYSIEILTQ

MQREQYKTTLDLSNGFWAHPIRKESYWLMAFTWEGKQLVWTRLPQGFINSPALFTANIVDILKEIPDVEVYVNDIYFSNV

TEEQHLITLKQVLKILLKSGYVSLKKSEIAKEEVTFLSFNITKEGHGLTAKFRSAPKTLKQLQSILGLLNFAHNVITDFA

ELTKPLYWMEKEGLQEIIKKLNNASYLENRDIQKPLIIKLNSSPTAGYIRMYNKGGKKPIQYVNFIFTPAEIKFKPTEKL
LTTMHKAIIKGLDLSQGAQVHIYSPLASPTHIQKTPLPERKGLHSQWITWMTHFKNPQLIFHHDPTLPDIQNLPYTAVYY
TDGSAIKNPNPQKTHSAGIGIVKGKFPNFSIIKQWRFPLGDHTAQYAEISALEFAVKKAMMDKGPILIVTNSMYLAKSFN
EELDIWISNGFVNNKKPLQHISKWKVIANCKQNKPSIHMVHEPGHQKQGTSIHTKGNLLADQLAVQSSHMVGMVPSLDKE
LEQVLDSPNPKGYPVKYIYLLENGNVIIEQDEGKRIIPPVMERVKLAQQAHNTIHGGWEATLIKLKNKYWWPNMIKTVRS
VVANCEKCQVTNASSQIPTPPKTIIHPDKPFEKFYMDYIGPLPSSHGHKHILVVDDARMGYCWLFPTKAQNANATVKALN
FLSGTAIPKVLHSDQGSAFTSATLQQWTKDRGIQLEFSTPYHPQSSGKVERKNGKIKRVLTKLLYGWPQKWYPLIPFVQL
SINNITPHKLMFGVDSNLPFANVDDANLSREEQLSLLQELREELWKPFIGQFIQERVQKYTPLCPRWKKPTKILTVFDDH
TVEILDPLGQRRKVSIDNLK
>TraEFVtja.a3
--PSLRNNITLIQKLNLIYKSSIIYKNKEYLKKKNSINTPIYPVPKNNER-KMVLNYREVNKVTPLITT-NQHSAEILSS
LYRDKYKSTIDLTNDFWAHSTTRESY-LTTFT-SKKQYC-TVLPQNVLYSPASFTRNVVDLLKEITNVQVYVDDVYLSHD
IFKKHLDTLKKMLQLLRETRYVSLQTSTLCQSTVEFLKFQITNTKRGLTSSFKKPLSILKILQSILDFINFTKNVLPNFA
KLVAPLY-TSKHTLTKLVNALNDTAKLQERDPILKLIIKCNASPATSYTKFYNKKKNSPIQYLSILFTKTKLKFSNLKKL
LTTIHKALLKRLDLTIGQKIIVYSPVVSMAKIQKTPITSKKTLASQWVTQMSYLKDPKIKFYYDNSLPKLKLLPYASVYY
TDGSTIKSPNKTKTYCSKMSIIQV-----------------HTANYEKIKC---SFKRSKNYSFKICLQTSSNTESYFNN
HGLSYYISIKILNIKYSQKTINKIKNISNCLKVKSDIFVAQEPGHQKTNVPLHAKDNTLADTLAVCTSYTVNTVAELNSK

LNQFLSKIIIKNYPTKFKYELQETNVITI-PKNIKILPPKSNKNKIIKKTQTLSYERHNTTMMILQQKH--PNIKKNVVK

YLRLYNTCTQVNPSNIKPSGPILQTRPPKPFKQFQINFIGPLPPPKNILD----------YTPLRPKPRQLSRPTYLVLQ

FPKCYTLIKGRHS---------FLPIWPLRLKKNILHNSILLTTPKTVKKNKINKIKRLLTKLLIKRPIK-YSLIPSIQF

ALNNTTPHQLMFKIDYNQPFANRDTFN-TPKKQLALLSKIHQSLSIPVIGQLVQKRVYRPSQLRPKWRKSTPIVAVLNNH

TVQIVDHLGQSKTVSVENLK

>TraEFVtka.a3

PQPQKQYYINPKAKPNIQIVINNLKQRILLKKKKNSINTPIYPVPKNNER-KMVLNYREVNKVTPLITT-NQHSAEILSS

LYRDKYKSTIDLTNDFWAHSTTRESY-LTTFT-SKKQYC-TVLPQNFLYSPASFTRNVVDLLKEITNVQVYVDDVYLSHD

IFKKHLDTLKKMLQLLREIRYVSLQTSTLCQSTVEFLKFQITNTKRGLTSSFKKPLSTLKILQSILDFINFTKNVLPNFA

KLVAPLY-TSKHTLTKLVNALNDTAKLQERDPILKLIIKCNASPATSYTKFYNKKKNSPIQYLSILFTKTKLKFSNLKKL

LTTIHKALLKRLDLTIGQKIIVYSPVVSMAKIQKTPITSKKTLASQWVT-MSYLKDPEIKFYYDNSLPKLKLLPYASVYY

TDGSTIKSPNKTKTYCSKMSIIQV-----------------HTANYEKIKC---SFKRSKNYSFKICLQTSSNTESYFNN

HGLSYYISIKILNIKYSQKTINKIKNISNCLKVKSDIFVAQEPGHQKTNVPLHAKDNTLADTLAVCTSYTVNTVAELNSK

LNQFLSKIIIKNYPTKFKYELQETNVITI-PKNIKILPPKSNKNKIIKKTQTLSYERHNTTMMILQQKH--PNIKKNVVK

YLRLYNTCTQVNPSNIKPSGPILQTRPPKPFKQFQINFIGPLPPPKNILD----------YTPLRPKPRQLSRPTYLVLQ

FPKCYTLIKGRHS---------FLPIWPLRLKKNILHNSILLTTPKTVKKNKINKIKRLLTKLLIKRPIK-YSLIPSIQF

ALNNTTPHQLMFKIDYNQPFANRDTFN-TPKKQLALLSKIHQSLSIPVIGQLVQKRVYRPSQLRPKWRKSTPIVAVLNNH

TVQIVDHLGQSKTVSVENLK

>TraEFVtja.b5

---------------------------------------MGKVTPIIATQNCHSTGILVS

I-RRKYKCTIDLANGFWAHPIARESYWITGFS-AGKEYCWTVLPPGFLNSPALSTRDIVDPLKEIKNVQVYVDDVYLCHD

TFEEHYNALDQMLMILKEATHVPLHKSQLFQKQVEFLGFQRTNKGMRLTDAYKTTSTTLEQFQSILEFLNFARNFLPNFS

KSIISSYLTKAHTLDRVFVDLNTQAEL---KPEKSLIIKCNISPTTSSIRFYNDGEHSPMQNMSVIFVKTKSKFTPLKKH

LS----------------------------------------------------

-----------------------------------------------------

-----YISNGFLNDRKPLVHIGIWNYIADALRLSQTFQCFMSQAIK--NYILLLTQWIIVWQIS----------PHINCM

LLENPQSYFQKAIDINIFTNLKMIESLLLGLKATRLFPLYLPVLKLFNKQAKLHIQAVRFHFLKLKNKYQWPNMR-NVAK

TLAMWETCHRVNKSTSEPQMPVSQGQPDKPFEKFQTD-ISPLPPSEGYLRLLVIVDMATGCLWLYPTKAQTAKAPIKALN

LLTGTVIPRVLYSGQGSAFTSSDLAA-AKDKGIQLEFSTPYHSTVRSGKEKQDETFDETLSKAKMKSEKNTY--APDIQF

VLNNTTAHQLMYGVDCNEPLANQVTFD--TEEQLAILKEIRESL--TKLSLLVQERVYRPSQL--KWRKLSPNVTILNN-

TVETVDHLGQLKMGSVDSLK

>ERV-Spuma-Smu

PKRQSQYKINHAAIPSIQIVIDDLVKQGIIKEQYSQMNTAVYPVPKPNGSWRLVLDYREVNKVTPVVSAQNTHSVSILHG

LVRKRFKTTLDLANGFWSHPIDTDSYWITAFTWQGKQYVWTRLPQGFVNSPALFTADVVSLLSAFSNIEVYVDDIYLSHD

SEEEHIAVLTQVLDTLYKAGYVSLKKSQIARNKVTFLGFDITHNGRGLSDNYKKRPCSLKQLRSIIGLFNYAMLFIPNFN

ILIAPLHWNMKEELQDLLTAVNN-DYLTQREPTQPLSVYVSISSEAAYIRIYNSAEIVPIVLLSYVFTITERKYLPLEKL

LTAVTVAVLKSRDLAQGQNIYIYTSVASLATLKKETIPEKRAFKCRWLKWLTLFEDPQLNFVYDATLPMLDDLPYTYLYY

TDGSAVNSNKEGVNRSAGVGIA--KYSDMSLLKTWQLPIGDHSSQYAEIYALVFALKEALSDKGPVLVVTDSAYLARSAN

EDLRIWRSNGFLTAKKPLMHIAKWKAISAYLNYKPDIEIVHEPAHRKLGSSAHTLGNQLVDSLAVSQKYAVNQVASLDAE

LNACLDDPNPPGYPRIYAYKVQDEHCIVTIKEVDYILPPKEERVRIANKAHAHPHLGRDNVLQALKKKYWWPNMRKTVSY

VVGNCKPCLLVNNPNHQKPPHIVKEIPNKPFDLIYLDHIGPLPPSHGYLYVLVCVDACTRFTWLYPVRATNSNTTLSCLN

FLVGAGKPKILHSDGGPAFTAVKTQEWAHALGIKWEFSSPYHPQSSGVVERKNAEIKRLLTKLLYSRPTQWYPLLPQVQL

GINNITPFELMYGVPMNTDFVDSSISAPGRLEQLSFLQELRDALWTPQVGHFVQERVSTIKPLRPRWKPPAEIKTVINER

TFIIVDKQGRQRTVSIDNLK

>ERV-Spuma.1-Cma_0

PKPQKQFKINPQAIPSIQIVINYLLKQGIPRQETSEMNTPVYPVPKGEGKWRLVLDYRAVNKVTPAIAAQSCHSTGILMQ

LTRKKYKTTLDLSRWVFGPILSPKIA----FTWCGKQHVWTQLPQGFLNSPALFSADVVDLLKEIPDVSVYVNDIYFLHD

TEKEHLKTTRHLHNI--ERGQHSLKKSEIGKREVNFLGFAITNEGRDLTD-YEQPPKTLKQLQSILGFLNFAHPFISNFA

ELVKSLHW--ENQLDDLITAIKQAALLTERDPTKPLAVKLHVSPEGLYVRLYNMADRFPFQYTSIIF-KAEKRFTLTEKL

LTVMQYALIKSFDIAQGQMIHVYSPLRCPETLQRHTIPERKVLSSRWLKWMSHIENPQIKFHYDEELPDLASLQYKQIYY

LYGSATTNEHKRQ---ARMDAVQAIFPDYQVLNVWSIPLGQHLAQYAEVAALEFALQQIPMDQTPRLIITDSDYVSKRYN

SKLEFWESNGFCNAKKPLHHISLWKSISELKKIKPWVHVTHEPGH-CIGTSVRRAGNAAADSLAKKAS-MINRVPTIDTD

LGQCINLPNPPGYKKK-----LDGIYWITKPEGEFQIPPTTERHMITERAHPHAHFGRDATLAVLK-KCWWPYMIQTVQQ

VLQYCSKCVTYNSANRAPIPHDKRTIPESPFDILFIDYIGPLPKCPGQDYVLVIIDGATSFVWLYPTTGPTAQATVRALT

DFCKIAIPKKIHSDQGPAFTADISKEFAKKYNIQWEYSTPYHPQNSGKVERANGEVKAALTKLSGSCPGK-YAYILLVQL

GFNNRTPFKLLFGVPMNVEF--NLTSDLSREEQLPLLAEIQYTLWHPLVGLLVQERVTTP--LRPRWKPPTLIIKVLSDR

VVEIVDKKGNLKQGSIDNLK

>ERV-Spuma-Cbo

-----QFKINPQAISSIQIVINYLLKQGVPR------NTPVYPVPKGEGKWRLVLDYRAVNKVTPAIAAQNCHSTGILMQ

LTRKKYKTTLDLSRWVFGPILSPKIANGQPLLGVAKQHVWTQLPQGFLNSPALFSADVVDLLKEF-DVSVYVNDIYFSHD

TEKEHLKTTRHLHNI--ERGQHSLKKSEIGKR-VNFLGFAIT-----------QPPKTLKQLQSILGFLNFAHPFISNFA

--VKSLHW--ENQLDDLITAI--AALLTERDPTKPLAVKLHASPEAGYIRLY-VGDRSPFQYISIVFKGAEKRFTSTEKL

LVVTQYALIKSFDIAQGQMIHVYSPLRSPETLQSHSIPERKALSS-WLKWMSHLENPQIRFHYDEELPDLTSSPYKRIYY

IDRRAITNEHKRQ---ARMGIVQDIFPCYQVLNVWSIPLGQHQARYAEAAALEFALQQIPMDQNPILIITDSDYVSKRYN

SKLEFWESNWFCNAKKPLHHISLWKSISELKKIKPWVHVTHEPGHRCIRTSVHTAGNAVADSLAKKAS-TINRKPTIDTN

LEQCINEPNLPGYYKNYKYHKDDGIYWITKPEGEFQIPPTTEGHLVTERAHSHAHFGRDATLAVLK-KHWWPYMIQKVQQ

VLQSYSRCITYNSANQAPIPHGKRSIPESPFDILFIDYI-LLPKCPGQEYVLVIIDEATSFVWLYPTAGPTAQATV-ALT

DFCETAIPKKIHSDQGPAFMADICKEFA-KYNIQWEYSTPYHPQSSGKVERANGEVKAALTKLSGSCPGK-YAYILLVQL

GFNNRTPFEFLFGVPMNVEF--NLTSDLSR-EQLALLAKIH-TLWHPLVGLLIQERVATQRPLQPQWKPPTPIIKIPSD-

VVEIVDKKGNVRQV-IDNLK

>ChrEFV

PIKQKQNHINHKATPSIQ-VIDNLLAQQ-LKKQTSPMNTPVYPVPKSEGKWRMVLDYRAINKVIEPIAAQNSFSTSILAQ

LPKKKFKTTLDLSNIF-AIPIHPNDYWITAFT-KSLHHVWTRLPQSFINIPALFPADIQQLVYLMSHMMTSISLQIQSNN

SYNKSY---------ISEMQDSSLPKSQPL-GDIQFLSV-LKSQNKAEAQKISKEPGSLKQLQSVLGLFNFARNFVPDFA

VLTELLCWNGEAALSTLINSVNKTKYLAE---NVKLLVKCYASQMAGFAGLHNESETTPISFLSFIFTPAETKFAPTERI

LTIIHKTLLKASDLAV-SIIKIYSPVTPPTKLQKIPLSERNALNSKWITWPSHFKNPQL-FIYDPNLPDLSHLPYTYVYY

TDGSAIKSSKINFQYSSWTGIIKGKFPSYTEVNKWSFSAGDHPAQYAEVTVFIFAYKDASKQVMPVLIVTDSDYVFKAYT

KESQIRISNGFHNAKKPIKRIVKWQQITSLY--VPKSHVIHEPGHQTPDPTKATTGNTIADPLATKASREVNVIPTENKD

LKQVKDLGNSK-YTVKYRYKTINDKPMILLPEGMEILP-YDQHFFYAIQTHQSLDLG-ESTITKLSKNI-WPSLRLTVDS

VLGSCETCLNTHSANVSP-PPFKLDKPENPFDKIYMDYVSPLPSSQEYLHLLVLVDYLTGF-FDTPHQRSTANTMVHALY

VFISVAIPKVTHADQGAAFTSGTLTKWAADHNIVLEFSTPYHPQS--KVEQKTSVIKCALTKLFAGRPQKRYPLIPLLQF

GINNIIPYQLLFGATPNTPFSGSNLTTKFNSTSANFFFPPCQPLLSPFGLTSLSRRYTEGTCLRPHWKKPTKFLQFLNPR

TAIV-EHLGQPRTVSIDNLR

>TraEFVtka.b2

SRPQEKYGIYPKTKLDIQIVINELIKQGFFNKTALIRQNILYP-----REWKLEFFHGAVNKLTPINAIHYCYSTRILAL

IYWGKYKSTINLVNGFWAHPILNESYWIKIFALSGKEYCWIVLPQVFLKSPVLFTGAIGDLRKEINSVQVYVGDVCLSHD

TFEECYNALDQMLMILKEERYISLHKSQLFQKRLNIL-------GPGLTDA-KTSPTTLNQLQSILEFVNFARNFLLNFC

TLITPSCGTKEHTLDRILTVFNTGTHLVEQKPKMKLIMKCNASPSTCYIRFYNDGENSPIQYKAIFFAETESKFTPLKKL

GFKI----------------------------------------------------------------------YVSIIY

----------------------------------------KHTKLH--IQAVRFRLS-----------------------

--------------------------------------------------------------------------------

--------------------------------------------------------------------------RQTLVE

YETRCGDCCYMNKPTLVPQMPVLQ-QLDKPFEKFQMDYINPLKPSEGYICILVIVDMASGYIWLYPFKAQTAKAPIKVLN

LLTLTVIPRVLHSGQATELRT-------REYNCLVLLITP----KEWDVTRRNSEIKKLLS----GRPIKQYNYIPDIQF

ALNNTTPHQLMYGGDCNQPLANQDTFDWTQNNKLAILKKICK--FDSEDSLLVQERMYILSQLQRKLTTLITSEKLLISR

--------------------

##ENV alignment

>FFVfca

VMTLKEWMEWNAHKYRCYTLCATSTRIMFWILFFLLCFSIVTLSTIISILRYQWKEAITHPGPVLSWQKLPVEVNISGIP

QGLFFAPQPKPIFHKERTLGLSQVILIDSDTITQGH--IKQQKAYLVSTINEEMEQLQKTVLPFDLPIKDPLTQKYIEKR

CFQKYGHCYVIAFNGWPSQDLIQDQCPLPPAFPKELVGSGMLFRPINPYDICNMPRAVLLLNKTYYTFSLWEGDCGHPYA

CRFWRDWGWLAYTDSFPSPICINLRRIQEAGLGLANAITTVAKISDLNDQKLAKGVHLLRDHVVTLMEANLDDIVSLGEG

IQIEHIHNHLTSLKLLTLENRIDWRFINDSWIQEELGVSDNIMKVIRKTARCIPYNVKQTRNLNTSTAWEIYLYYEIIIP

TTIYTQNWNIKNLGHLVRNAGYLSKVWIQQPFEVLNQECGTNIYLHMEECVDQDYIICEEVMELPPCGNGTGSDCPVLTK

PLTDEYLIEPLKNGSYLVLSSTTDCGIPAYVPVVITVNDTISCFDKEFKRPLYAPSVPQLELRVPRLTSLIAKIKGIQIE

ITSSWETIKEQVARAKAELLRLDLHEGDYPEWLQLLGEATKDVWPTISNFVSGIGNF

>SFVggo

PMSLQQWIIWNKMHYFLYTCCATSSRVLAWMLLACILFIIIIVSCFITLSRIQWNKDIQVLGPVIDWNPKYVEVNMTSIP

QGVFYQPHPEPIIHTERVLGLSQVLMINSENVANSANLSQETKVLLTEMINEEMQSLSDVMIDFEIPLGDPRDQEYIHRK

CYQEFAHCYLVKYKTWPNEGLIVDQCPLPGALPYEWGGLNRLFRNISVLDVCSRPEMVLLLNKTYYTFSLWEGDCNHPYS

CRLWRDFGFLSYMKNFPGPQCINYSKLRSMGYALTGAVQTLAQISDINDQNLQQGIYLLRDHIVTLMEATLHDISIMEGM

FAVQHVHTHLNHLRTMLMERRIDWTYMSSSWLQTQLQKSDDEMKVIKRTARSLVYYVKQTYNSLTATAWEIGLYYELIIP

RHIYLNNWQVVNIGHLIKSAGQLTHVTVSHPYEIINRECSNTLYLHLEECRRLDYVICDVVKIVQPCGNSSDSDCPVWAE

PVKEPHVISPLKSGSYLVLASSTDCQIPPYVPSVVTVNETTQCFGVTFKKPLLEPQLPHLQLRLPHLVGIIAKIKGIKIE

VTSSGESIKDQLERAKAELLRLDIHEGDTPAWIRQLAAATEDVWPAAASALKGIGNF

>SFVpve

PMTLQQWIIWNKMNYLLYTCCATSSRVLAWMLLVCVLLIVVLVSCFLTISRIQWNRDIQVLGPVIDWNPKYIEVNMTSIP

QGVYYEPHPEPIVVTERVLGLSQVLMINSENIANNANLTQEVKKLLAEVVNEEMQSLSDVMIDFEIPLGDPRDQEYIHRK

CYQEFAHCYLVKYKTWPTEGLIADQCPLPGALPKEWQGKNALFKEINVLDVCSKPELVILLNTSYYSFSLWEGDCNHPYA

CRFWRDFGFLAYQKNFPAPICINYAKLKSMGYALTGAVQTLSQISDINDENLQQGIYLLRDHVITLMEATLHDISVMEGM

FAVQHLHTHLNHLKTMLLERRIDWTYMSSAWLQQQLQKSDDEMKVIKRIAKSLVYYVKQTYNSPTATAWEIGLYYELTIP

KHVYLNNWNVVNIGHLVQSAGQLTHVTIAHPYEIINKECTETKYLHLKDCRRQDYVICDVVEIVQPCGNSTDSDCPVWAE

AVKEPFVVNPLKNGSYLVLASSTDCQIPPYVPSIVTVNETTSCYGLNFKKPLFEPRLPNLQLRLPHLVGIIAKIKGLKIE

VTSSGESIKDQIERAKAELLRLDIHEGDTPAWIQQLAAATKDVWPAAASALQGIGNF

>SFVppy

PMTLQQWLVWDRMQYLLYTCCATTTRTLAWLFLFCVLLIVVLVTCFITIARIQWNQDIQVYGPVIDWNPKNVEVNMTSIP

QGVYYEPHPEPIIVKERVLGLSQVIMINSETVANSANLTQEAKVLLADMVNEELQGLADVMIDFEIPLGDPRDQDYIHRK

CFQEFAHCYLVKYKDWPSEKLIVDQCPIPGALPPSWEGKSLLFREANTLDICNIPEAILLLNTTYYNFSLWEGDCGHPYA

CRFWRDFGFLAYLGHFPSPICINYVKLRSMGYSLTGAVQTLSKISDINDENLQQGLYLLRDHLVTLMEATLHDISLMEGM

LAVQHLHTHLNHFKTMLLERRIDWTFINSDWLQQQLQQPTDHMKIIKRTARSLVYYVEQTSNSPTATSWEVGIYYEIIIP

KHIYLNNWQIKNIGHLIHSAGQLTHVTIDHPYEILNRECEETKYLHLEQCIKQDYVICDIVERVQPCGNTTGTDCAVYAK

AIKSPYTILPLKNGSYLVLSDSTSCNILPYIPSIVTVNETVECFGVLFKKPLYTPHIPPLRLRLPHLLGIIAKLKNIKIE

VTSTQENIKDQIERAKAELLRLDIHEGDSPAWIKQLAAATEDVWPTLATGLKSIGNF

>SFVcae

PMNLQQWLLWKKMNYLAYACCATSTRVMCWLFLICVLLIIVFVSCFVTVARIQWNRDINVFGPVIDWNISYISINMSSIP

QGVMYTPHPEPIILKERVLGISQVLMINSENIANVANLSQETKVLLTDMINEELQDLSNQMIDFELPLGDPRDQDYIHHK

CYQEFAHCYLVKYKKWISEGIIVDQCPLPRALPKEWQGNARLFRSFNPLDVCNRPEAVLLLNTTYFTYSLWEGDCNHPYA

CRFWRDFGFLSYLNAFPGLKYINIEKLRSMGYSLTGAVQTLSQISDINDERLQQGVSLLRDHVVTLMEAALHDITIMEGM

LAIQHVHTHLNHLKTILLMRKIDWTFIKSNWIKEQLQKTEDEMKIIRRTAKSLVYYVTQTSSSTTATSWEIGIYYEITIP

KHIYLNNWQVINIGHLVESAGHLTLIRVKHPYEVINKECTYEQYLHLEDCISQDYVICDTVQIVSPCGNSTTSDCPVTAE

KVKEPYVVSALKNGSYLVLTSRTDCSIPAYVPSIVTVNETVKCFGVEFHKPLFEPQVPHLKLRLPHLVGIIANLQNLEIE

VTSTQESIKDQIERAKSQLLRLDIHEGDFPAWIQQLASATRDVWPAAARALQGIGNV

>SFVaxx

PLTLQEWMLWNKKRYLCYLACATTTRIVGWIVFVCLLIAVICITCFVTMARMQWKQAIITHGAVIDWNEKWIEINATGIP

QGVYLTPHPKPIIVKERILGLSQIVLINSESIASSMEIKQEHKSLLTKIINEEMKSLRDVMLDFELPLDDPKTQKYIQQR

CFQEFKDCYLVKYNTWPTDYVLQDMCPLPGAMPREWSGQNALFRNITRIDYCMLPEAVILLNTTKTDYSLWEGDCNHPYT

CRHWRDFGWLAYNGNFPSPVCENWNKLQKAGYAITNSVKQIAQISDLNDESIVSGLYLLRDHVVTLMEATLHDVSALEDS

IAIQHFHTHLMQLKLLLMENRVDWSYIDTQWIKTQLQLNDEDMKVLRRTARALVYNIDHIEDTKTSTIWEIAMYYEIIVP

SVIYSTNWNVQNVGHLIASAGSLTLVKVKHPYEIINQECGIIKYLHIENCQETDYVICDTIEEVQPCGNQTGSDCPVLAE

PVPDGFHIESLKNGSYIYMSHYQDCSLTPYIPQVVTVNATIKCLGRNLKPPLLTPQVPRLKIQLPHLVGVITKLKGIQVK

ITSTWETIKDQIDRAQAELLRLDLHEGDSANWLKQLSKATEDIWPAAAATFGKVGDF

>SFVcja

PMSLTEWILWNKKRYTCYLACATTTRIMIWILFCVIVLTVALIICFTTAARIQWRHAIITPGPVIDWNERYVEVNATGIP

QGVILLPHPKPIIQKNRVLGLSQILLINSESLASIFNIKQEHKSILTEIIQEEMRSLQDITLNFDLPIGNPKTQHYIQSR

CFQEFKDCYLVKYQDWPTDDVLADMCPLPGALPAEWQGQNRLFRNLSRIDYCKLPEAVVLLNSTKYDYSLWEGDCEHPYT

CRHWRDFGWLAYNNHFPSPVCVNWQKLQKAGYAITNAVTQIAKITDLNNEAIVSGIYLLKDHIVTLMEATLHDVSALGNV

VTIQHFHTHLAQFKLLLVENRIDWNYIDSRWIQDQLGLDEADMKILRRTARALIYNVEEIDFRPTSTTWEIALYYEIIVP

GKVYSTNWEVHNIGHLVDSAGSLTLVTIQHPYTIVNQECGETKYLHMEECTEQDYKICEQVTEVLPCGNLTGSDCPVLAK

TVKPGYVIESLRNGSYIYMAHYQDCGIKPYVPQIVTVNATVKCLGYEIQPPLLTPQVPSLKLRLPHLVGILAKLKNIQIQ

VTSTWESIKDQVEKSQTELLRLDIHEGDTPAWIKQLAESTKDIWPTTANIFGKVGEF

>SFVssc

PMTLHEWLKWKKTNYMCYLACATTTRIMGWIIFTLIIASVILVTCFVVMARIQWRNAITVPGIILDWNENIVEINTTSLP

QGILFEPHPKPIIGKERVLGLSQVILINSESIATSLEIKQEHKHILVEMIKEELLSLQNVMLNFDLPLGDPKTQQYISQR

CFQEFKHCYLVAYNEWPTDDVVQDMCPLPGALSAYWQGQNKLFRNITRLDYCKYPEAVILLNTTKSDYSLWEGDCNHPYT

CRHWRDFGWLAYQGHFPSPICENWGKLQAVGFTITNTVSKIARIIDLNNEHLVSGLYLLKDHLVTLMESTLHDISILGNA

VAIQHFHTHLTQLKLLLMENRMDWTFIDSSWIQDQLKLSDEDMKILRRASRALVYKVEEIGEGVTSTIWEIGIYYEIIIP

RVIYSTNWKIMNLGHLVYSADNLVQINVEQPYEILNVECGKSTYLHIDKCEEQDYVICEVIQEKQPCGNQSGSDCPVKAR

TIEKGYTIQPLKNGSYVVMSHFQDCHIKPYIPQIVTVNATVKCLGEVFQPPLVLPVTTSLKLQLPHLVGIITKLKGFQVQ

ITSTWESIKGQVEQAQAELLRLDLHEGDSGQWIKQLASASKDIWPAAATVLGKIGDF

>SFVocr

PMTLQQWLRWRNMNFRLYRLCATTTRTMGWCIGLFCLLLILLFSLVIVILRLQWRNAIVTPGPIIAWNPVDIEINITALP

QGMLLVPHTKPVVKKERALGFSQIIIMSSDSMANSMGLKKEDIHLLVDLLNEEMEQLQNIILEFDLPIGDPHDQSYIEQR

CKAALQHCYVVEREGWPTDGAILDQCPLPDALPPDWQGQNRLFRSITTFDVCQRPEMVFLLNTTYYTYSLWEGDCNHPYA

CRHWGDFGYLAFTDMFPAPTCINFHKIQSIGFNLANAISTVSKISDLNDNQLAKGMHVLRNHLVTLMEATLHDISKFESG

LALQHLHTHLAQLRSTLQENRVDWSILDTAWIQSELNTDDNTMKLIKRTAKAMVHHVQQTQKSLRATTREVGIYFEIIIP

AAIYTQNWQPLNLGHLVFNSGQLTQVFVEQPYNLVSMECNIPTYLHIEECVNQDYLICDIVEEVLPCGNQTGSDCPVMAK

AVKAPFVITPLKNGSYVILADTSACTIPAYSPVLVTTNDTLQCYGHILKRPLYEPRVPDLVIRLPHLVGVIAQLKDLKFQ

VTSSWESIKDQIARSKELLLQLDLHEGSAPEWINRLAAAAADIWPATGQALKGLGDF

>EFV

PMTLPEWMQWRYRQYWMYLLCATSPRVMAWLLFVCVLISVMIIAVIVTVFRMQWKAAIDVPGPVLFWNDRAVNINITHIP

QGVFLEPFPKPIIDKERVLGISQIVMIDSGSIAQSMNLDLYMKHLLVDMINEEMVALSNVVLPFELPVGDPSTQDYIHKR

CYQQFAHCYIVWQPGWPTSEIIQDQCPLPDALPPTWKGQARLFRELNPLDFCTKPEAVMLLNQSYYTWSLWEGDCYHPYA

CRHWRDFGFLAYQKMFPSPICINFRKLQTSGLSMNQAISTLAKISDLNDENLAAGIHLLQEHIVTLMEATVHDISMLEAA

HGLQILHTHLSTLRLLLTENRVDWNLIDSTWIQQQLQADEALMNVIRRTARSMTYRVIQQINRPDMTLWELGIYYELIIP

KKVWLTNWKIQNIGHLIKNAGHLARVELQHPYEIVNQDCEQLTYLELKGCQELDYLVCEEILQHEPCGNQTGSDCPVTAQ

KIKDPYVIYPLKNGSYLIMSSHTDCAIPPYEPVLVTVNDTVRCFGTTLKKPLFQPHIPQLQVRLPHLVGLIAKIKGLKIE

ITSTWENIKDQIKRSEAELLRLDLHEGDYAEWTKQLGKALEDIWPAAAQTVSKIGDF

>BFVbta

PMTLQQWLQWRYNLYWIYLCCATSTRIMAWIVFILTVLSILLISVLIAVFRLQWKGAIESPGPILVWNQKPVQVNFTSIP

QGLFLEPHPKPIISKERVLGLSQVVMVDSSTLTQKLNLEGEAKSLLIKTINEELISLQDVVLNFDLPLGDPHTQEYIAKR

CYQHFGHCYVVHIPGWPTREIIQDQCPLNNALPGTWLGKGEWFRDLTTYDFCKKPEAVFGLNKTYYSWSLWEGDCGHVYG

CRYWRDLGYLAYLGAFPSPICINWRRLQITGQSMNQAITTLSKLSDLNDENLAAGIHLLQDHIVTLMEATLHDVSLLGHM

TSIQHLHTHLATFKNLLIGNRVDWSVLENKWIQEELKYTDEVMNVIRRTARSITYDVQNVKNTSDSTMWEIYIYYELILP

ERIWIRNWQVANLGHLTHNSGYLTHVTIHHPYEIVNQDCEELTFLHLVDCHEQDYLICEEVMEVEPCGNLTGSDCPVLAE

NIQAPYVLHPLKNGSYLLMASHTDCSLPPYEPVVVTVNDSLECYGKPLKRPLFAPQIPQLRVRLPHLVGIIAKLKSLKIK

VTSTWESIKDQIHRSEQELLRLDLHEGDYSDWILQLGNALEDVWPVAASAVSTIGTL

>TraEFVtja.b2

PITLWEWLLWRHATYCSHLACATTTRLIGRLLV-IILLAIVLAATVIALFRLQWRNAVETPGPVITWNPRPENIQAYPLQ

QGIVFEPHPRPIMAKERFLAASQVVMTDTCTVLSETNLDSRQAEFLTQMINEERKDLQSLILPFDLPMGDPTSQDYIQKR

CFQTMGHCYLVDFGQWPTDNLIQDQCPLPGSLPPDWQGSGEVFQSLNPMDSCMRPRAVILLNSTYFKWSLWEGNCGYPYT

CRHWRDFGFLAYHNHFPSPICINFRRIQAAGLGLTGAITMIAQLSDLNDQNLASGIHLLKDHIVTLMEAIISDIDLLAGA

IEVEHLHTHVSHCKNMFLDSRIDWKVIGSEWIQKGLQLSDKVMKIIRSTATSITYRVEQSRDEAQATAWEL---------

-----HNWELKNIGHLVRNSGSLTRV-IEHPYQILNQDCQRTRYLKLVDCCIKDYLVCEEVIEMLPCGNQTGQDCPVKAL

KIEDSFHIQPWKDGSYLLMTDKTECGLTPYAPSIITVNNTVVCYCEELKRTFLRPHIAQLSMRLPHLIGIIAKLKNIAIE

ITSTSEGIKDQIARAREELLRLDLHEGDLSEWLNQLAQATQDIWSATETTIQGIGNV

>TraEFVtka.b3

PITLWEWLLWRHATYCSHLACATTTRLIGRLLV-IILLAIVLAATVIALFRLQWRNAVETPGPVITWNPRPENIQAYPLQ

QGIVFEPHPRPIMAKERFLAASQVVMTDTCTVLSETNLDSRQAEFLTQMINEERKDLQSLILPFDLPMGDPTSQDYIQKR

CFQTMGHCYLVDFGQWPTDNLIQDQCPLPGSLPPDWQGSGEVFQSLNPMDSCMRPRAVILLNSTYFKWSLWEGDCGYPYT

CRHWRDFGFLAYHNHFPSPICINFRRIQAAGLGLTGAITMIAQLSDLNDQNLASGIHLLKDHIVTLMEAIISDIDLLTGA

IEVEHLHTHVSHCKNMFLDSRVDWKVIGSEWIQKGLQLSDKVMKIIRSTATSITYRVEQSRDEAQATAW-L---------

-----HNWELKNIGHLVRNSGSLTRVWIEHPYQILNQDCQRSRYLKLVDCCIKDYLVCEEVIEMLPCGNQTGQDCPVKVL

KIEDSFHIQPWKDGSYLLMTDKTECGLTPYAPSIITVNNTVVCYCKELKRTFLRPHIAQLSVRLPHLIGIIAKLKNIAIV

ITSTSEGIKDQIARAREELLRLDLHEGDLPEWLNQLAQATQDIWSATETTIQGIGNV

>TraEFVtja.b3

PMTLQEWILWRHATYCSYLACATTTRIIGWLLLFIILLAIVLATTMITLFRLQWRTVVETPGAVITGNP-PVNIQAYPLH

QGIFSEPHPRPIMAKERVLATSQAVMIDTGTILSETNLDSRHAEFLTQMINEEMKESQSLTLPFDPTMGDPTSQDYIQKR

CFQTMGHCYLVDFGQWPTDNLIQDQCPLPGSLPLDWQGSGEIFRSLNPMDSCMRPRAVVLLNSSYFTWSLWEGDCGHPYK

CRHWRDFGFLTYHSHFPSPICINFRRIQAAGLGLLGTITMIAQLSDLNDQNLASGIHLLKDHIVTLMEATISDIDLLKGA

IEVEHLHSYVSHFKNMLINNRVDWKVIGSEWIQKGYSFQMRKLGELQPALPIEWNKLERSPRLLLGKYIYIYIYYEIIIP

KEVFTQN-ELKNIGHLVWNSRYLTRVWIEHPYQSLNQDCQRTRYLKLGDCYIKDYLVCEEVIEMFPCGNQTGQDCPIKAL

KIDDSFHIQSLKNGSYLFMADKTDCRLKPYAPSIITVNDTVVYYGEELKRAILRPHIPQLTVRLPKLTGIIAKLKSIQIV

IMSTSEGIKDQIARAREELLRLELHKGDLPEWLNQLAQATQDIWPARATTIQGIGNF

>TraEFVtka.b1

PMTLQEWILWRHATYCSYLACATTTRIIGWLLLFIILLAIVLATTMITLFRLQWRTVVETAGAVITGNPRPVNIQAYPLH

QGIFSEPHPRPIMAKERVLATSQAVMIDTGTILSETNLDSRHAEFLTQMINEEMKESQSLTLPFDPTMGDPTSQDYIQKR

CFQTMGHCYLVDFGQWPTDNLIQDQCPLPGSLPLDWQGSGEIFRSLNPMDSCMRPQAVVLLNSSYFTWSLWEGDCGHPYK

CRHWRDFGFLIIVIFPVLFVSTNFRRIQAAGLGLLGTITMIAQLSDLNDQNLASGIHLLKDHIVTLMEATISDIDLLKGA

IEVEHLHSYVSHFKNMLINNRVDWKVIGSEWIQKGLQLSDEVMKIIRRTATSITYRVEQTREEPQATAWE----------

----------------------------------LNQDCQRTRYLKLGDCYIKDYLVCEEVIEMLPCGNQTGQDCPIKAL

KIDDSFHIQSLKNGSYLFMADKTDCRLKPYAPSIITVNDTVVYYGEELKRAILRPHIPQLTVRLPKLTGIIAKLKSIQIV

IMSTSEGIKDQIARAREELLRLELHKGDLPEWLNQLAQATQDIWPARATTIQGIGNF

>TraEFVtja.b6

PMTHQEWLLWRQKQDCSYLACTTTTRVLC-ILLFTLLLAIILAALMISLFRLQWRTVVETLGPTITWNFRPVNIQVYPLP

QGIYY-PHPRPILAKERVLAASEVVMIDTATILPETGLNSEHEKLLTQMINEEMKDLQELTLPFDLPIGDSTIQKYTQKR

CFQTMGHCYIINLEQSPTDNLIQDQCPLPR-----WQGESGLFRSLNPMDSCMRPRAVILLNST-YTWSLWEGDCGHLYT

CRHWRDFGFWAYHQHFPSPICINFGRIEAAGLGLMGAITTIVRLRNLNDQNLASGIHLLRDHIVTWMEATINDIGLLT-A

IEVEHIHTHISQLKNTLINN-VDWKVISSYWIQNGL-LTEEIMEIIRRTATSITYRVEQTRNGPLATTWEK---------

KEVYTQN-EIKNIGDLVRTSGHLTKIWIEHLYQILNQDCQ-TRYLKLIDCYIKDYLICKEVVEMLPCGNQTGQDCPIKAL

KIEESF-----KNGGYLFMTDKTDCGLEPYVPSIVTVNDTVMCHSKELKRAILRPHIPQLPVRLSHLIGMTTKLKGIEIT

ITSTSEGIKSQVARARGELLHLDLHEGDLPE-LN-LTQATQDIWPLAANMIKSIGNF

>TraEFVtka.b4

PMTHQEWLLWRQKQDCSYLACTTTTRVLC-ILLFTLLLAIILAALMISLFRLQWRTVVEIPGPTITWNFRPVNI-VYPLP

QGIYY-PHPRPILAKERVLAASQVVMIDTATILPETGLNSEHEKLLTQMINEEMKDLQELTLPFDLPIGDPTIQKYTQKR

CFQTMGHCYIINLGQSPTDNLIQDQCPLPR-----WQGESGLFRSLNPMDSCMRPRAVILLNST-YTWSLWEGDCGHLYT

CRHWRDFGFWAYHQHFPSPICINFQRIEAAGLGLTGAITTIVRLRNLNDQNLASGIHLLRDHIVTWMEATINDIGLLTEA

IEVEHIHTHISQLKNTLINN-VDWKVISSYWIQNGL-LTEEIMEIIRRTVTSITYRVEQTRNGPLATTWEKYIYYEIIIT

KEVYTQN-EIKNIGDLVRTSGHLTKIWIEHLYQILNQDCQ-TRYLKLIDCYIKDYLICKEVVEMLPCGNQTGQDCPIKAL

KIEESFYNVPQQR------AQKSN---------------------------LRPHIPQLPVRLSHLIGMTTKLKGIEIT

ITSTSEGIKSQVARARGELLHLDLHEGDLPEWLN-LTQATQDIWPLATNMIKSIGNF

>TraEFVtja.b1

PMTRQEWLLWRQKP---YLAYATTTRVLCWILLF-ILLAIMLAALTILLFRLQWRTVVKTPSPIITWNFRPVNIQVYPLP

-GIYYEPHPRPI----------------------QKMGLNLEHAKLLTQKINEEMKDLQ-VTLPFDLPIEDPTTQEYIQKR

CFQTMGHCYIINFGQVPNDNLIQDQCPLPGNLPPEWQGESGLFWSLNPMDSCMRPRAVILLNSTYYTWSLWEGDCSHSYT

CRHWRDLGFLAYHRHFPSPICINFQRIQAAGLGLTGAITTIAR-SPTNDQNLASGIHLLRDHIVTLMEATINDIGLLTEV

IEVEHIHTHISQFKNMLRNNQVDWKVISSDWIQNGLQLTDKIMKIIRRTAISITYRIEQTRNGPLATTWEIYIYNEIIIP

KEVYTQNWEIKNIGHLVKNSGHLTKIWIEHLYQILNQDCQRTRYLKLIDCYIKDYLICKEVVEMLPCGNQTGQDCPIKAL

KIEDSFHIQSLKNGSYLFMTDKTDCGLEPYVPSIVTVNNTVMCYGKELKRAV--PHIPQLSVRLPHLIGIIAKLKGIEIT

ITSTSEGIKSQVA-ARGELLCLDPHEGDLPEWLNQLAQVIQDIWPLAANTV------

>TraEFVtka.b5

-------------NYSYLSWATTTRVLCWILFFILLMAIILAALMISFFKFQWRTVVETPGAIITWNFRPVNIQVYPLP

QGIYYEPYPRPIMAKERVLATSQVVMIDTAT-------------------------------------------------

------------------------------NLPPE-KGKNGIFQNLNPMDSYMRPRAVI-LNSTYYTWSLWEGDCGHPYT

CRHWRDFVFLAYHRHFPSPICINFRKIQYKQLGLTGAITT-ARLSDLNDQNLASRIHLLRDHIVTLMETTVNDIGLLTEA

IEAELIHTHIS-FQNMLINN-VDWKVISSD-IQNGLQLTDKIMKIIRRIATSMTYRIEQTKNEPLATTWEIYIYYEIIIP

KEVYTQN-EIKNTGHLVKNSEHLTKIWIENPYQILNQYCQQTRYLKLIDCYIKDYLICKEVVEMPPYRNQTGQDCPIKAL

KIKESFHIQPLKNRNCL---QKIDCRLEPYIPPIITVSDTV---GKELRRAILRPNIPQLSVRLPPLIGITAKLKDIEIT

ITSTSERIKSQVARARGKLLHLNLHDGDLPEWLNQLAQATQDIWPLATNTIKGIGNF

>TraEFVtja.b7

PMTL-EWLLWRQKPNYSYLSWATTTRVLCWILFFILLMAIILAALMISFFKFQWRNVVETPGAIITWNFRPVNIQVYPLP

QGIYYEPYPRPVMAKERVLATSQVVMIDTAT-------------------------------------------------

------------------------------NLPPE-KGKNGIFRNLNPMDSYMRPRAVI-LNSTYYTWSLWEGDCGHPYT

CRHWRDFVVLAYHRHFPSPICI------YKQLGLTGAITT--ALSDLNDQNLATRIHLLRDHIVTLMETTVNDIGLLTEA

IEAELIHTHISQFQNMLINN-VDWKVISSD-IQNGLQLTDKIMKIIRRIATSMTYRIEQTKNEPLATTWEIYIYYEIIIP

KEVYTQN-EIKNTGHLVKNSEHLTKIWIEHPYQILNQDCQQAKYLKLIDCYIKDYLICKEVVEMPPYRNQTGQDCPIKAL

KIKESFHIQPLKNRNCL---HKIDCRLEPYIPPIITVSDTVMCYSKELRRAILRPDIPQLSVRLPPLTGITAKLKDIEIT

ITSTSERIKSQVARARGKLPHLDLHDGDLPEWLNQLAQATQDIWPLATNTIKGIGNF

>TraEFVtja.b5

PMTL-EWLLWRQNPNYS-LVCATTTRVLCWILLFILLMAIILAALMISLFRLQWRSVVETPSPIIN-NLRPVNIQVYPLP

-GIYYEPQLRPIMAKERMLAASQVVMIDTAT-------------------------------------------------

------------------------------------------------------------------------------YT

CRHWRDFGFLAYHRHFPRPICINFWRIHATGQGLTRTIAKMAQFSDLRDQNLASGIHLLRDHRVILMETTINDIGLLTEA

SETEHIHAHIWQFKNMLINNQAD-KVISTDLIQNGVQITDEIMKVIRRTATSVTYRKDQARNEPQATTWKICIYYEIIIP

REMYTQNWEIKNIGHLFNNSRHRTK------------------------------------VVVMLPCGTQTG-DSPIKAL

NAEESIHIQPLKNESYLFMTDKTDSRLEPYVPSIVTVNDKVTCYGKELKRVILRPHIPDLSVRLLHLIG--GKLKGIEIT

ITGTSKARQSQVAQAREELLCLDLHEGDLPEWLNQLAQATQDFWPLAANMIKGVGNF

>TraEFVtja.b4

PMTL-EWLLWTQKPNYSYLACTTIIRVLC-ILVFILLMAITLAALMV-FFRPQWSTVAKASSPIITWNCKPLNMPVYPLP

QDIYY-PHQRPVMTKERVLAASQIVMIDSTS-------------------------------------------------

------------------------------NLLPK-QGENEKFQSLNSMDSYMRLRAVILVNCTYYT-SLWEGDCEHPYT

YRP-RDLEIWAYHSHFHSPICINFWIIQATVLGLTEA-TTIARLGDLNDQNLTSGIHLLRDHIVTWMEATINDIGLLTEA

IETERMHTHISQFKNMFINNKE--KERSSDWTQNGLHHTDEILKIIQRAATSITYKIEQIQN-------------EIIIA

KDVYRQNCQIKNIGHLVKNSGYLTKIWIEHPYKILNQDCQRTTYLKLIDCYIKDYLICKEFVEMLPCGNQTGQDCPKKEL

KIEESFHIQPLKNESYLFMTDKTVCGLEPYAPSIVTVNDTVMCYSKELKR--LTPHIPHLSVRLPHLIGIITKLKGIEIT

ITSNSEEMKSQVVQAREK----------------------------------------

>TraEFVtja.a2

PMAMQEWMQWKQKQYWLYLTCATLMRVLGW--------------------RLQWKAAIEAPGPVINWTTKPVNITTHPLP

QGIFFEPHPWPILTKEKVLAISQMIIINTEPITVGTDLTTAQA-YLTQMVNKEMRDLQSLTLPFDLPIGDPTSQEYIKRK

CFQNLGHCYLI---------------------------------------------------------------------

-----DFGYLAFLRIFPSPICINFRKVQTAGLGLAGAISTVTRLSDLNDK-LASGIYLLKEHVITLMETTIADVGLVAGA

IQVEHFHNHISQFKHMLINNQVDWNVMDSN-IQEELQLSKEIMRIMHRTANSITYRVEQTKNFLHTTAWEIYVYYELIIP

KEVYTQNWGIKNLGHLVRNSGYLTKVCVEHPYQILNRDCEETRFLQLKDCYIKNYLIC-KVKEVQPCGNQTGQDCLVKAS

KISEPWAIQPLKSGSYLLMADSTDCGIEPYVPALITVNDSVTCYSYEFKKALLIPQIPPLRIRLPHLLGIIAKLKNLEIT

ITSIAETINDQLERTKNELLRLDTHEGDLPHWIWQLATAAKDFQPTTAHTIQDIGHF

>TraEFVtka.a1

--------------YWLYLTCATLMRVLGWVFLLVLFL---------TLFRLQWKAAIEAPGPVINWTTKPVNITTHPLP

QGIFFEPHPWPILTKEKVLAISQTIIINTEPITVGTDLTTAQA-YLTQMVNKEMRDLQSLTLPFDLSIGDPTSQEYIKRK

CFQNLGHCYLIDFGV----------------------------------------------------------------

-----DFGYLAFLRIFPSPICINFRKVQTAGLGLAGAISTVIRLSDLNDK-LPSGIYLLKEHVITLMETTIADVGLVAGA

IQVEHFHNHISQFKHMLINNQVDWNVMDSN-IQEELQLSKEIMRIMHRTANSITYRVEQTKNFLHTTAWEIYVYYELIIP

KEVYTQNWGIKNLGHLVRNSGYLTKVCVEHPYQILNRDCEETRFLQLKDCYIKDYLIC-KVKEVQPCGNQTGQDCLVKAS

KISEPWAIQPLKSGSYLLMADSTDCGIEPYVPALITVNDSVTCYSYEFKKALLIPQIPPLRIRLPHLLGIIAKLKNLEIT

ITSIAETINDQLERTKNELLRLDR--------------------------------

>TraEFVtja.a3

SMTMQEWMQWKQKQYWLYLTCATLTRILGWVLLLVLLLTIIIAAIITSLFRLQWKATIEAPGPVINWTTKLVNITTHPLP

QEIFFKPHPRPILAKERVLAISQVIIINTESITAGTDLTTAQAEYLTQMVNKKLKDLQSLILPFDLPIEDPTSQKYIKKE

CFQYLRHCYLVDFGVWPDSTFLQDQCPI--MLSPDWNGKNKLFLNVTLTDSCLRLLLPILINSTRYGWSL-DGDCEHPYA

CQQWRDFRYLAFLRNFPSPICINFRKIQTAGLGLAGAISTVARLSDLNDQKLASSIHLLKEYVITLMEATIADAGLVARA

IQMEHSHNHLSQFKHMLINN-VD-NVINSN-IQKELQLSKKTMKIIHRTTNSITYRIKQMRNFLHAAA-EIYGYYKLIIP

RKVYTQN-KIKNLGHLIRNSRYLTKVWVKHPYQILNRDCKEIRFLQVKDCYVKDYLICEEVRKVQSCENQTGQDCLVKAL

KITEA-TIQPLKSKSYLLMTNSTDFRIEPYVPTLITVNDSVTCYGYEFKKALLIPQIPPLRIHFPHLLEIIAKLKNLEIT

ITSTAKAINDQLERTKNELLRLDIHKRDLPHWIQQLATATKNIWPATAHAIQGTENI

>TraEFVtka.a3

SMTMQEWMQWKQKQYWLYLTCATLTRILGWVLLLVLLLTIIIAAIITSLFRLQWKATIEAPGPVINWTTKPVNITTHPLP

QEIFFKPHPRPILAKERVLAISQVIIINTESITAGTDLTTAQAEYLTQMVNKKLKDLQSLILPFDLPIEDPTSQKYIKKE

CFQYLRHCYLV-------------------MLSPDWNGKNKLFLNVTLTDSCLRLLLPILINSTRYGWSL-DGDCEHPYA

CQQWRDFRYLAFLRNFPSPILLNFRKIQTAGLGLAGAISTVARLSDLNDQKLASSIHLLKEHVITLMEATIADAGLVARA

IQMEHSHNHLSQFKHMLINN-VD-NVINSN-IQKELQLSKKTMKIIHRTTNSITYRIKQMRNFLHAAA-EIYGYYKLIIP

RKVYTQN-KIKNLGHLIRNSRYLTKVWVKHPYQILNRDCKEIRFLQVKDCYVKDYLICEEVRKVQSCENQTGQDCLVKAL

KITEA-TIQPLKSKSYLLMTNSTDFRIEPYVPTLITVNDSVTCYGYEFKKALLIPQIPPLRIHFPHLLEIIAKLKNLEIT

ITSTAEAINDQLERTKNELLRLDIHKRDLPHWIQQLATATKNIWPATAHAIQGTENI

>TraEFVtka.a2

--------------HWLHSSRAILTRILGWVFLVVLFLAIITTTIITTLFRLQWKAAIETPGPVINWTTKPVNVTTHPLS

QGIFFEPHP-PILAKERVFAISQVIMINTETIIAETDLTTAQAEYLTQMVNEEMRNLKSLILPFDLPIGDPTSQ-YIRKK

CFQDLGHCYLVDFGTWPDSTFIQDQGPIPGLLSPNWNGENKLFQNVTLTDSCTRPLLPILLNSTRYEWSLWEGDCGHPYA

CLHWRDFGYLA--SYFPSPMCK---KVQTTVLGLVSAINTVARLSDLNDQNLASGIHLLKDHIITPMEATIADVSLVTGA

IQVEHFHNHISQFKHTLMNN-VDWNIMDSAWIQEELKLSEEIMRIMRRTANSITYRVEQTRNLLNASAWKIYVYYELTTP

KEVYTQHWEIKNLGHLVRNSGYLTIVWVEHPYQILNRDCEKTRFLQLKDCYIKDYLIYEEVKEVQLCGNQTGKNCLVKAS

KISEPWVIQPLKNGSDLLMTDSTDCGIEPYVSALIIVNDS-------FKRALLIPQVPPLRIRLPHLLGIIAKLKNFEVT

ITSTAEAINDQLERTKNELLRLDIHEGDLPHWIQQLATATKDIWPAAAHAIQGIGNF

>TraEFVtja.a1

--------------HWLHSSRAILTRILGWVFLVVLFLAIITTTIITTLFRLQWKAAIETPGPVINWTTKPV------LS

QGIFFEPHP-PILAKERVFAISQLIMINTETIIAETDLTTAQAEYLTQMVNEEMRNLKSLILPFDLPIGDPTSQ-YIRKK

CFQDLGHCYLVDFGTWPDSTFIQDQGPIPGLLSPNWNGENKLFQNVTLTDSCTRPLLPILLNSTRYEWSLWEGDCGHPYA

CLHWRDFGYLASLNIFPSPMCK---KVQTTVLGLVSAINTVARLSDLNDQNLASGIHLLKDHIITPMEGTIADVSLVTGA

IQVEHFHNHISQFKHTLMNN-VDWNIMDSAWIQEELKLSEEIMRIMRRTANSITYRVEQTRNLLNASAWKIYVYYELTTP

KEVYTQHWEIKNLGHLVRNSGYLTIVWVEHPYQILNRDCEKTRFLQLKDCYIKDYLIYEEVKEVQLCGNQTGKNCLVKAS

KISEPWVIQPLKNGSNLLMTDSTDCGIEPYVSALIIVNDS-------FKRALLIPQVPPLRIRLPHLLGIIAKLKNFEVT

ITSTAEAINDQLERTKNELLRLDIHEGDLPHWIQQLATATKDIWPAAAHAIQGIGNF

>sloEFV

VLNLQDWMVWNR--YFLYTLCATSTQILCWFFFGLIIIGLILGFILSAVFRLQWKNAIHHPGPIISWNPRNVHLEICGLQ

QGMFWEQFPKPIIHKKRTLGISQILLIDTPLVWY---IPLKDKKILTQLIDNEFAQLQEIVLPFTLPLDQPYTQEYQQKG

CFQEFGHCYLVKY--WLTSKIIQDHCLIPTALPSEW--QNNLFKSPTITQFCNHPELIYFLNTTYTTYSLWEGDC-HPYT

CQFWRDWRQLAYSKKFPAPIC-NTQRLEKVSLMMANSTATVSKLSDLNEYLFADGLHILKDHVVTLLEANMKDTQHIDEL

TTAMLILSYIQNFRIPSTEGRIDWRILNGTWINEGLNIPHHGMQIVKRMSCSNIYDIKKTIK----SIWEIGIYYQIILP

NKVFYTNWQVLNIGHLVKTGTQLTLTKIHQPYTHISE-CSELYYLEPKGCEQRDYLICEEINLHQTCGNKT-SKCPVTGK

AV--PYLFIPLKNGSYVVMSYTIDCNIPPYQSSIFTINDTVTCFEKILKKHL---HIPKIQLRLPHLVGILAKLKKIEVK

ATDTWASIEEQIEDTKSDLLRLELHKGDTPEWIKQLGEALEDVWPAAASATKTIASF

>CmaEFV

--------------YSGYAAYATSTRIAMWIIFTLLILMTMLGVTCTVIVRLQWKYAIERQGPTITWNPLLVNVPITGLK

QGLY-EPFPKPIVAKERVLGISQILILDSDHMAEANNLGHVGKEILTQLLNE---------IPFEIPLDGPQTQQYMQKK

RCHEFAHYYWIDYKEWPESQVIADHCPHHGALLNDWQGKGRWFKPMDDLSFCRHPELTVFLNGTYHKHSCMEGDCQHPYA

CQFYRDTGLLLHDHTFPGFWCINLQKIQEAGLIPDSSITKTAKISDLNDK-LAKGLHLLRDHLITFPEHTI-DVIQMSQS

IIAVMTHSHIQNLRILLTEGKVDWDTLNSTWIQEQLRVSDEMMTLIRRTARGLAYDIQQRVDKPEKGVWEISLYYEIVIP

R-IYSTNWKIINYGHLVYTGNRGGRVWLKHPCTLITQGCGEVKYLEVRECYEQDYLICDEVIKHEPCGNQTGSRCPIMVE

PIVSPYLIGPLKNGNYIVTTSLDECSIPPYQPSLITVNETVTCYGYEFKSPLENIHMPPLSVSLPHMIGIIADLRQIKIE

LASSWDSVHDVTERANTELLRIDLREGYTPQWLNRLSESIADIWPAAAGAIKGIANG

>ChrEFV

-LSLMEWMRWDLERYYCYVSCANTTRVMT-SYVSLILLGIIITASFITICRIQWQQTIEAQGPTIFIGEDP-NITVTTIP

QGHHWDPYPKPIETKERVVAISQVLLLNTKSILDAAKITDKMKTKIKAAMHKEM----DLADQTYLPMEDSFNQAYEDKL

CFDNFGDCYVVDYQE-PIKEIIQDQCPPKVAFPEDWEGKGKIFRPFKMDSFFKEPELVMHLNRTYDAFNSWENDNPHSYN

CDFQHNFLLISKDLHLIPLI--NIYTL--FKLLIEKSMNKLIQL-DWNNQQLENGFHLLQTHVITLFESTRYDLQILMA-

-----IQQQLEKIKNQARMGHMDCAIVSWNWLQEQLNLTDAQMTLVKETSSARINNVIMGED-----HYEIHIHCELIIP

KEIYATNQKLLN--YLVSQNQHLLKLTIGSQYQYLNYETGQLKYLQVQGCTEDKFLMCDKIKEVPPCGISNNSNCPVYVQ

TIKDPYLIIPLQNSSYIILIE-SNCYIPAYQPIVITTKYPVHCQENVLSPPLLVLDLPQIHWRLPHLLGTLTELKGYKLK

FYNLYDNLQDILDQIRSTLLRYNVHERDLLAWLTQVSKALEDIVPAAVNLISKVTAG

##GAG alignment

>TraEFVtja.a2

EIHLQDAQGQPLSQPEWVPMQAPTYPIHDLLITGPLNQLQAAFDLLDCALGPE-HGPLHEGCMQTPINHIRAVIGNTPNN

PKDIALWLGRAIPAIEGMFPIPDKPTRIRVINALLAGLAGMDLKQGQAPAWDAAIALIYQRTHGAAAAHTLAETLTSIYR

SDGILVAFNMEMIFSERDFALVWGIVRGLLPGQALVTNAQQQLNAYPDDQAKAQAFPQIILGLYQLLGLNMHGQSI

>TraEFVtka.a1

---LQDAQGQPLSQPEWVPMQAPTYPIHDLLITGPLNQLQAAFDLLDCALGPE-HGPLHEGCMQIPINHIRAVIGNTPNN

PKDIALWLGRAIPAIEGMFPIPDKPTRIRVINALLAGLAGMDLRQGQAPAWDAAIALIYQRTHGAAAAHTLAETLTSIYR

SDGILVAFNMEMIFSERDFALVWGIVRGLLPGQALVTNAQQQLNAYPDDQAKAQAFPQIILGLYQLLGLNMHGQSI

>TraEFVtja.a4

--RLQDVQGQPLPQSDWIPIQGPADPIHDFLITVPLNQLQVAFDLLDYAPGPKMHAPLHEGHIQIPINHIRAVIGNTPNN

PKDIALWLGRAIPAIEGVFPIPDEPTRVRVINALLAGLAGMDLRQGQAPTWDAAIALIYHRTHGAAAAHTLAKTLASIYC

SDGILVAFNMGMMFSDRDFTLVWGITRGLLPGQALVTNAQQQLNAYPDDQAKAQAFPQIILGLYQLLGLNMHGQSI

>TraEFVtka.a4

--------------------------------------------------------------------------------

---------------------------VRVINALLAGIAGMNLRQGQAPTWDAAIALIYQRTHGAAAAHTLAETLGSIYH

SDGILVAFNMGMMFSDRDFLLAWGIIRGLLPEQTLVTNAQQQLNAYPDDQAKAQAFPQIILGLYQLLGVNMHGQSI

>TraEFVtka.a2

KICLQDAHGQPLPQPEWVPIQGPADPIHDLLITGPLNQLQAAFDLLDCAAGPERHGPLYEGHMHIPVNHIRAVIGNTANN

PNDIALWLGSAIPAIEGVFPIPDKPTRIRVINALLAGLAGMDLRQGQAPTWDAAIALIYQRTHVVAAAHTLAETLASIYC

SDGILVAFNMGMMFSDQDFALVWGIVHNLLPGQALVTNAQQLLNAYPDDQAKAQAFPQIILGLYQLLGLNMHGQSI

>TraEFVtja.a1

KICLQDAHGQPLPQPE-VPIQGPADPIHDLLITGPLNQLQAAFDLLDCAAGPERHGPLYEGHMHIPVNHIRAVIGNTVNN

PNDIALWLGSAIPAIEGVFPIPDKPTHIRVINALLAGLAGMDLRQGQAPTWDAAIALIYQRTHVAAAAHTLAETLASIYC

SDGILVAFNMGMMFSDQDFALVWGIVHNLLPGQALVTNAQQLLNAYPDDQAKAQAFPQIILGLYQLLGLNMHGQSI

>TraEFVtja.a3

RIHLQNAQGQPLPQPEWVPIQDPADPIHDLLITGPLH-LQAVFDLLECAPGPKKHGPLHERHMQIPINHIRAVIENTPNS

PKDIALWLDRAIPAIERMFAIPDEPTRIRVSNTLLAGLAKIDLRQRQAPT-DTTIALIYQKTLRAAATHTLAETLTSIYR

SDKILVTFNMKMMFSDRDFALVWGIIRGLLP-QALVTNAQ-QLNAYPDDQAKTQAFPQIILRLYQLLGLNIHGQSI

>TraEFVtka.a3

RIHLQNAQGQPLPQPEWVPIQDPADPIHDLLITGPLH-LQAVFDLLECAPGPKKHGPLHERHMQIPINHIRAVIENTPNS

PKDIALWLDRAIPAIERMFAIPDEPTRIRVSSTLLAGLAKIDLRQRQAPT-DTTIALIYQKTLRAAATHTLAETLTSIYR

SDKILVTFNMKMMFSDRDFALVWGIIRGLLP-QALVTNAQ-QLNAYPDDQAKTQAFPQIILRLYQLLGLNIHGQSI

>TraEFVtka.b1

KVLLQDP-GQHLPQPQWQIALGNVDLANDYLIAELYNQMIQVFMGLDLLPGPKRHGPLREVGLQIPRNHVRAVVGNTPTN

PQDVTLWLRRALPAIEGIFPVPDEQTRIRVINALLAGLASVHLQPGQTPT-DAAIALIYQCTNGAAATHTLAETLATVYR

SEEILVAFNMGMMFSGRDFTLVWDIVCGFSPGQAIVTNAQQQLDTFPNDQA-ADAFLQIILGLYQLLGLNVHGQSI

>TraEFVtja.b3

----------------------------------------------------------------IPRNHVRAVVGNTPTN

PQDVTLWLRRALPAIEGIFPVPDEQTRIRVINALLAGLASVHLQPGQAPT-DAAIALIYQCTNGAAATHTLAETLATVYR

SEEILVAFNMGMMFSRRDFTLVWDIVCGFSPGQAIVTNAQQQLDTFPNDQA-ADAFLQIILGLYQLLGLNVHGQSI

>TraEFVtja.b7

EILFQDAQGQPSPQLQWRVLPGDIDLVHDYLITGPHNQIVQVFVGIEPSAGPERHGLLQERRLQVPINHITAVVRNTPTN

PQDVTLWLGRAVPAIEGVFPVPDEPTRIRVINTLLAGLAGIHLQPGQAPSWDDAIALIYQCTHGAAATHTLAETLATIYR

SDGILVAFNMGMMFSGRDFTLNWKIIRGFLPGQAIVTNAQQQLD--------------------------------

>TraEFVtka.b5

EILLQDAQGQPSPQLQWRVLPGDIDLVHDYLITGPHNQIVQVFVGIEPSAGPERHGLLQERRLQ--INHITAVVRNTPTN

PQDVTLWLGRAVPAIEGVFPVPDEPTRIRVINALLAGLAGIHLQPGQAPSWDDAIALIYQCTHGAAAAHTLAETLATIYR

SDGILVAFNMGMMFSGRDFTLIWRIIRGFLPGQAIVTNAQQQLDQRPQPFHK-------LFWLHQLLGLNVHGQSV

>TraEFVtka.b2

EILLQDGQGQTLPQLEWV-LSGNIDLVHDYLIAGTHNQIVQVFIGIEPSAGPERHGPL-EGRLQIRINQRSTVVGNMSTN

PQDVTLWLGRAVPAIEE-FLVANEPTRIWVINVLLAGLAGIHLQLGQTSSWDDAIALIYQHTHGAAVVHTLAEALATIYR

SDGILVAFNIGMVFSGRDCTLLWGIIRGFLPGKAIVINAQAKLDSFPDNQAKTTAFPQIILGLY-LLGLNVHGQSI

>TraEFVtja.b4

KTLSKDALGQPLSHLQWQVLPEGIDLVHNYLIAQTNNQIVQIFLDIVPSAGPERHGPL-EGGFQ---NHTRAVVGNMPTN

PENVTLWLG-AMPAIEGVFLVPDESTRIRLINALLVGLVGMHLQLGQASTWEYAISLIYQCTHGTAASHTLMETLANIYL

SEGILIAFNIENMFSGRHITLIWGIVWGFLPGQTIILNAQQQPDAFPDSQAKAMAFPQIILGLYELLRLNDHGKSI

>BFVbta

RIHLQDPAGQPLPIPQWEPIPRTANPRTQPVVSAPMATLENILNNFHIPHGVSRYGPLEGGDYQLPITHIRAVIGETPAN

IREVPLWLARAVPALQGVYPVQDAVMRSRTVNALTVRHPGLALEPLECGSWQECLAALWQRTFGATALHALGDTLGQIAN

SDGIVMAIELGLLFSDDNWDLVWGICRRFLPGQAVCVAVQARLDPLPDNATRIVMISHIIRDVYAILGLDPLGRPM

>EFVeca

RLRLVDNTGQPLAQPEYEDTGLPAENRG-IVVAVSHNAARNIFNNVQPAGGPNRHGPLHDGQFQLPITHIRAVIGETPAQ

IRDVPLWLAQSIPALTGVYPAMDAGTLTRLVNAITARHPGLALGMNEAGSWHEAVHLIWQRTFGATALHALSDVLKGIAQ

RNGVVMALEMGLMFTNDDWDLTWSVIRRCLPGQASVVTIQARLDALPNNQARIIQAGFIIREVYEVLGLDPLGRPL

>FFVfca

TIRLQDNTGQPLQVPGYDLEPGIINLREDILIAGPYNLIRTAFLDLEPARGPERHGPFGDGRLQIPINHLRSVIGNTPPN

PRDVALWLGRSTAAIEGVFPIVDQVTRMRVVNALVASHPGLTLTENEAGSWNAAISALWRKAHGAAAQHELAGVLSDINK

KEGIQTAFNLGMQFTDGNWSLVWGIIRTLLPGQALVTNAQSQFDLMGDDIQRAENFPRVINNLYTMLGLNIHGQSI

>SloEFV_gag

QIDLQDGNRQPLPQPTYTPAPGPVDLTTDILLNVSYAQLMAKLGDVPFLTGVYRHGPLYTGHWLLPMNHLRAVVGATPND

PQAIALWLGRNVQAIEGVMPINNGPMRKQVVNALLASHATLHVTDQEAQDWNSTIAAIYQRAHGTLALHHLPTVLKDIAN

SDGVIVAFTMGMMFSNDDYALVSGIIRPLLPGQAAVVAVQAQLDILPDDNAKASAFPEIVTEVYQTLGLNILGQPM

>PSFVaye

CLAFQYQNGQPSPVPP-RLIAEPVNLQNDYLVEASHNLLIQTFHNIAPG---VKHGPLSDGNYQIPINHITAVIGQSPEN

PEEISFWLGKNRAGLTGIFPVNNPNIRLRVINTFVGSHPTLILQQGEADTWEDTIVNLHQRAHGAVARHRLPGLITVIFK

KEGVQVAYSMGLMLS-KDFDLVWGVIGGLLSGQALVTNIQGQY--LLPNTARQEQFPGVVAT--NLLGLDVFGKSM

>SFVcae

SIFLQDDSGQPLQQPRWRPEGRPVNPLVHNTIEAPWGELRQAFEDLDVAEGTLRFGPLANGNWIIPIQHIRAVTGNTPTN

PRDIPMWLGRHSAAIEGVFPMTTPDLRCRVVNALIGGSLGLSLEPIHCVNWAAVVAALYVRTHGSYPIHELANVLRAVVT

QEGVATGFQLGIMLSNQDYNLVWGILRPLLPGQAVVTAMQQRLDQEVNDAARITSFNGHLNDIYQLLGLNARGQSI

>SFVcpz

RLILQNDDNEPLQRPRYEVIQRAVNPHTMFMISGPLAELQLAFQDLDLPEGPLRFGPLANGHYVIPIQHIRSVTGEPPRN

PREIPIWLGRNAPAIDGVFPVTTPDLRCRIINAILGGNIGLSLTPGDCLTWDSAVATLFIRTHGTFPMHQLGNVIKGIVD

QEGVATAYTLGMMLSGQNYQLVSGIIRGYLPGQAVVTALQQRLDQEIDDQTRAETFIQHLNAVYEILGLNARGQSI

>SFVpve

RLILQDEDNEPLQRPRHEIIPRAVNPHTMFVLSGPLAELQLAFQDLDLPEGPLRFGPLANGHYVIPIQHIRSVTGEPPRN

PREIPIWLGRNAPAIDGVFPTTTPDLRCRIINALLGGNLGLSLTPGDCITWDSAVATLFIRTYGQYPLHQLGNVLKGIAD

QEGVATAYTLGMMLSGQNYQLVSGIIRGYLPGQAVVTAMQQRLDQEIDDQTRAETFIQHLNAVYEILGLNARGQSI

>SFVggo

RLILQDDDGEPLQQPRYEVVPRAVNPRTQFVISGTLAELRLAFLELDLPEGALRYGPLANGHYIIPIQHIRAVTGEPPRN

PREIPIWLGRNAPAIEGVFPVNSPEVRCRVINAIIGGNLGLALTPTECATWDSAVATLFIRTHGTYPMHQLGNVIKGIVD

QEGVATGYTLGMMLSGQNFPLVYGIIRGFLPGQAVVTAIQQRLDQEVDDQTRSDTFIQHPNAVYEILGLNARGQSI

>SFVppy

RIILQDDDGEPLQVPRWEEVLRPVNPLAHFVISAPWDQLRRAFHDLDVGNGALRFGPLANGNYIIPIQHIRAVTGEVPNN

PRDIPMWIGRNAPAIEGVYPVTTPDLRARIINALIGGKSGIHLTAPEAVTWASAVAAIFTRTHGSFPMHNLSAILTGIAN

GEGVESAYNLGMMLSNGDFNLVYGIVRGLLPGQAAVAYMQQRLDAEPSDALRAQNFIQHLHLVYEILGLNHRGQSI

>SFVaxx

RVAFQDNQGNPLPRPEWEYINRDVRPLNDSIIGCTFRQAQQAFNNIDLARTPSRYGPLSNGLFLLPIAQIKAVIGEAPTD

ARQIPLWVAKHAAAIEGTFPTGSADVRCRVLNALLTSHGGMTLSPNECGTWSLAAAALYQRIYGVIPLHDLPHTMAEVAR

REGILVAFNMGMTFTNNSFDVVWGIIRPLLPGQASVAMLQGYLDQYQGQQQKAQAFPTLLRRTFETLGLNYLGQSI

>SFVssc

RIILQDESGNPLSQPRWELVDRMFNPLRDPILETTLDEMDRVFDGINLSPGTERYGPLCDGNFLLPISQIRTVIGNTPVD

PKKVPLWIAKSASAIEGVMPTNTPDIRCRLVNALLPQHGGLILQPHECNSWTQIASALYTRVNGMIPLHALPQTLSQVTK

EEGILVAYQIGMTFTGQNFPLTWGILRPLLPGQAVVAMMQGYLDQYPTDDLKAVNFASILRRVFDILGLNYMGQNI

>SFVcja

RVVFQDTSGNPLSQPQWMDEDRQMNPLQDPVVGCTFNQASRVFDQIDIGEGPSRFGPLADGMFLLPISQIKAVIGETPTD

HKAVPLWVAKHAAAIEGVFPTGSPEVRCRVLNSLLTGHGGMMLHPVDCVSWTNAASVLFQRVHGVVPLHQLPKTLEEVAK

TEGLLVAYNIGMTFTSNNFDLIWGIIRPLVPGQAAVAMLQGYLDQYPRPQDKIEHFPRFLRRTFEVLGLNFLGQSI

>SFVocr

EVLLRDTLG-PLQQPRYRYAAQQADLRNDIILHLNYQDAIIIFDMIIPSEGVHRHGPMFDGLWIIPINVIRSVCGDTPSN

PQDIPLWMGRIIPAIEGVFPIDNPNLRMRVVNALLALHPGLAITELNAQTWGQVLAVLHMRALGHTALHQLPALLETIVK

TDGILPAYNMGMEVTQQDFSYVWGILRTLLPGQAFVLSMQNELDRLPAAQ-RPGMFPGLLQRTLDILGLNSRGQNI

>CboEFV

----------------------------------------------------------------LPFSNIRAAVGQTPMD

IKKIFSWMAERINILEGVLPHMNNATRRQVVNSLVPYQ--LSLNEEECVSWDQIISCLYTKAHGHIPTAKLGEELQRISS

EQGIKTAFQSGLAMTNQNYGHLWGTIKNLVPGQAPLAEITRRLEALPSDQEHIRQFSTIVDTVYRMLDLDPTGKRT

>CmaEFV

RLELQDQGGANLPIPQWQHQDGRIERTE-IVIHANFAETLNWLGQPDINTGEDRHGPMAHEPFTLPFSNI-AAVGPTPMD

IKNIFSWVAERINVLEGVLPHMNNAARRQVVNSLVPYQ--LSLNEEECVSWD-MISCLYSKAHGHIPTAKLGEELQRISS

EQGIKTAFQLGLAMTNQNYGHVWRIIKNLVPGQAPLAEITRRLEALPSDQERIRQF-TIVDMVYRMLDLDPTGRRT

##POL-ENV alignment

>BFVbta

PRPQKQYHINPRAKADIQIVIDDLLRQGVLRQQNSEMNTPVYPVPKADGRWRMVLDYREVNKVTPLVATQNCHSASILNT

LYRGPYKSTLDLANGFWAHPIKPEDYWITAFTWGGKTYCWTVLPQGFLNSPALFTADVVDILKDIPNVQVYVDDVYVSSA

TEQEHLDILETIFNRLSTAGYIVSLKKSKLAKETVEFLGFSISQNGRGLTDSYKQKLMDLQPPTTLRQLQSILGLINFAR

NFLPNFAELVAPLYQLIPKAKGQCIPWQLKTIIQALNSTENLEERRPDVDLIMKVHISNTAGYIRFYNHGGQKPIAYNNA

LFTSTELKFTPTEKIMATIHKGLLKALDLSLGKEIHVYSAIASMTKLQKTPLSERKALSIRWLKWQTYFEDPRIKFHHDA

TLPDLQNLPLLHYEAIFYTDGSAIRSPKPNKTHSAGMGIIQAKFEPDFRIVHLWSFPLGDHTAQYAEIAAFEFAIRRATG

IRGPVLIVTDSNYVAKSYNEELPYWESNGFVNNKKKTLKHISKWKAIAECKNLKADIHVIHEPGHQPAEASPHAQGNALA

DKQAVSGSYKVFSNPSLDAELEQVLSTNPQGYPNKYEYKLVNGLCYVDREEGKIIPPKADRVKLCQLAHDHLGRSALLLK

LQQKYWWPRMHIDASRIVLNCTVCAQTNSTNQKPRPPLVIPHDTKPFQVWYMDYIGPLPPSNGYQHALVIVDAGTGFTWI

YPTKAQTANATVKALTHLTGTAVPKVLHSDQGPAFTSSILADWAKDRGIQLEHSAPYHPQSSGKVERKNSEIKRLLTKLL

AGRPTKWYPLIPIVQLALNNTPNTRQKYTPHQLMYGADCNLPFENLDTLDLTREEQLAVLKEVRDGLWTPSPGLLVQERV

ARPAQLRPKWRKPTPIKKVLNERTVII-DHLGQDKVVSIDNLKPAAHQYWIYLCCATSTRIMAWIVFILTVLSILLISVL

IAVFRLQWKGAIESPGPILVWNVQVNFTSIPQGLFLEPHPKPIISKERVLGLSQVVMVDSSTLTQKLNLEGEKSLLIKTI

NEELISLQDVVLNFDLPLGDPHTQEEYIAKRCYQHFGHCYVVHIPGGKEWPTREIIQDQCPLNNWDYYNQPPPRQLLNPE

ALPGTWHTLGKGEWFRDLTTYDFCKKPEAVFGLNKTYYSWSLWEGDCHVYGCRYWRTYDLGYLAYLGAFPSPICIEYKVT

SIYAECVKRLQITGQSMNQAITTLSKLSDLNDENLAAGIHLLQDHIVTLMEATLHDVSLLGHMTSIQHLHTHLATFKNLL

IGNRVDWSVLENKWIQEELKYTDEVMNVIRRTARSITYDVQNVKNTSDSTMWEIYIYYELILPERIWIRNWQVANLGHLT

HNSGYLTHVTIHHPYEIVNQDCEELTFLHLVDCHEQDYLICEEVMEVEPCGNLTGSDCPVLAENIQAPYVYLHPLKNGSY

LLMASHTDCSLPPYEPVVVTVNDSLECYGKPLKRPLPQIPQLRVRLPHLVGIIAKLKSLKIKVTSTWESIKDQIHRSEQE

LLRLDLHEGDYSDWILQLGNALEDVWPVAASAVSTIGTLLEKAAGTLFGNVFSILAYAKPVIIGIILIILLLLVIRILRW

L

>EFVeca

PKPQKQYRINPKAKADIQIVIDDLLKQGVLKQQTSPMNTPVYPVPKPDGRWRMVLDYRAVNKVTPAIATQNCHSASLLNT

LYRGQYKTTLDLANGFWAHPIQESDQWITSFTWNGKSYVWTTLPQGFLNSPALFTADVVDLLKDIPNVEVYVDDVYFSND

TEEEHLKTMDLLFQKLQTAGYIVSLKKSKLGQHTVDFLGFQITQTGRGLTDSYKSKLLDITPPNTLKQLQSILGLLNFAR

NFIPNYSELITPLYQLIPLAKGIYIPWILQKIIKELNASENLEQRKPDVELIVKVHVSPTAGYIKFANKGSIKPIAYHNV

VFSKTELKFTITEKVMTTIHKALLKAFDLAMGQPIWVYSPIHSMTRIQKTPLTERKALSIRWLKWQTYFEDPRLIFHYDD

TLPDLQNLPLSEYEVVFYTDGSSIKSPKKDKQHSAGMGIIAVRYQPQMNIIQEWSIPLGDHTAQFAEIAAFEFALKQAIR

KMGPVLIVTDSDYVAKSYNQELDFWVSNGFVNNKKKPLKHVSKWKSIADCKKHKADIHVIHEPGHQNDLQSPYAMGNNAA

DKLAVKASYTVFSVPSLDAELHQLLDKNPKGYPSKYEYTLRDGQVYVKRTDGKIIPSKDDRVKILELAHKHLGKNTMYIK

ILNKYWWPNLIKDISKYIRTCTNCIITNTDNVPNKSYIVQEKTGLPFQKYYMDYIGPLPPSDGYYHVLVIVDEGTGYTWL

YPTKAQTANATVKALNHLTGTAIPKVLHSDQGSAFTSATLVAWAKDKGIQMEYSSPYHPQSSGKVERKNSEIKRLLTKLL

VGRPTKWYPLIPTVQLALNNTPNAKIGKTPHQLMYGVDCNLPFQDLSTLDLTREEQLAVLQEIRTALWTPCPGLLVQERV

NRPAQLRPKWKKPTPILKVLNPKTVVIAGPGGQERIVSIDNLKKTPHHYWMYLLCATSPRVMAWLLFVCVLISVMIIAVI

VTVFRMQWKAAIDVPGPVLFWNVNINITHIPQGVFLEPFPKPIIDKERVLGISQIVMIDSGSIAQSMNLDLYKHLLVDMI

NEEMVALSNVVLPFELPVGDPSTQDQYIHKRCYQQFAHCYIVWQPGRRVWPTSEIIQDQCPLPDYDLYLQPPRYGLLKDR

ALPPTWTPKGQARLFRELNPLDFCTKPEAVMLLNQSYYTWSLWEGDCHPYACRHWRLLDFGFLAYQKMFPSPICIQYKVQ

SLYQECIQKLQTSGLSMNQAISTLAKISDLNDENLAAGIHLLQEHIVTLMEATVHDISMLEAAHGLQILHTHLSTLRLLL

TENRVDWNLIDSTWIQQQLQADEALMNVIRRTARSMTYRVIQQINRPDMTLWELGIYYELIIPKKVWLTNWKIQNIGHLI

KNAGHLARVELQHPYEIVNQDCEQLTYLELKGCQELDYLVCEEILQHEPCGNQTGSDCPVTAQKIKDPYVWIYPLKNGSY

LIMSSHTDCAIPPYEPVLVTVNDTVRCFGTTLKKPLPHIPQLQVRLPHLVGLIAKIKGLKIEITSTWENIKDQIKRSEAE

LLRLDLHEGDYAEWTKQLGKALEDIWPAAAQTVSKIGDFLGKIADGIFGTTFSLLTYAKPVIIGIVVIVLLILIIRILSW

L

>SFVocr

------------------------------------MNTPVYPVPKPDGKWRMVLDYRAVNKTVPAIGAQNCHAPGILSS

LYRAKFKTTLDLSNGFWSHPITPESYWLTAFTWQGSQYVWTRLPQGFLNSPALFTADVVDLCKHIPNVSAYVDDIYVSND

TAEEHLRTLEQLFRTLMSAGYIVSLKKSKIGVSAVDFLGFEITDDGRGLTSAFKEKLVNIQPPSSLKQLQSILGFLNFTR

NFVPNYSELVKPLYNLVATAQGNRISWQLNQVISALNAADNLSERKTGVPLVVKSNCSPTAGYIRFYNQGDRKPIQYVNY

IFSKTELKFTPLEKQLTVLHKAILKGLDLAGGEDIHFYTPIASISKLQRTPIPERKALHVRWLTWITYLEDPRFHFYYDE

TLPPLAELPLKEYTSVFYTDGSAIKNPNPKKAHSAGMGTVEVTYNPEYKVLHEWSFPLGDHTAQYAEIAACEFAIKKASL

LRGPVLIVSDSVYLVKSFNEELPFWISNGFLNNKKKPLQHISKWKTIAACYQNKKDIFLLHVPGHQKLLTDEHAQGNALA

DKLAVQSSHKVLFIPSLDAELIQVMEGYPKGYPHKYVYAQDNGKIIVTLPNGREIPPVGDRLALITKAHNHMGREAVLAK

IQNVYWWPNMKKDVKHVLTICSQCQQVNSFNLKPQPPQTIARHVHPFDKIYMDYIGPLPPSDGYLYVLVLVDSCTGFTWL

YPTKAPSANATVKALTHLTGTAVPKVLHSDQGSAFTSSTLVDWAKERGIRLEYSTPYHPQSSGKVERKNSEIKRLLTKLL

VGRPLRWYPLIPTVQLALNNTPNVSSGKTPHQLLFGVDCNLPFANKDTLDLTREKQLSLLKELREDLWRPSVRLLVQERV

YRPSALRPKWRKPTPILEVHSDRLVTIKDHLGNIKKVSTDNLKLTPHQFRLYRLCATTTRTMGWCIGLFCLLLILLFSLV

IVILRLQWRNAIVTPGPIIAWNIEINITALPQGMLLVPHTKPVVKKERALGFSQIIIMSSDSMANSMGLKKEIHLLVDLL

NEEMEQLQNIILEFDLPIGDPHDQSYYIEQRCKAALQHCYVVERE-GKGWPTDGAILDQCPLPDWDYYLQPPAFVKLKDR
ALPPDWTTQGQNRLFRSITTFDVCQRPEMVFLLNTTYYTYSLWEGDCHPYACRHWGLYDFGYLAFTDMFPAPTCIEYKVY
SAFQECMIKIQSIGFNLANAISTVSKISDLNDNQLAKGMHVLRNHLVTLMEATLHDISKFESGLALQHLHTHLAQLRSTL
QENRVDWSILDTAWIQSELNTDDNTMKLIKRTAKAMVHHVQQTQKSLRATTREVGIYFEIIIPAAIYTQNWQPLNLGHLV
FNSGQLTQVFVEQPYNLVSMECNIPTYLHIEECVNQDYLICDIVEEVLPCGNQTGSDCPVMAKAVKAPFVSITPLKNGSY
VILADTSACTIPAYSPVLVTTNDTLQCYGHILKRPLPRVPDLVIRLPHLVGVIAQLKDLKFQVTSSWESIKDQIARSKEL
LLQLDLHEGSAPEWINRLAAAAADIWPATGQALKGLGDFLQSTVGSLLGTGLSFLSYLKPILIGIGLIFLVVILFKIISW
L
>SFVcja
PRPQRQYHINTKAKPSIQQVIDDLLKQGVLIKQTSVMNTPIYPVPKPDGKWRMVLDYRAVNKTVPLIGAQNQHSLGILTN
LVRQKYKSTIDLSNGFWAHPITKDSQWITAFTWEGKQHVWTRLPQGFLNSPALFTADVVDLLKNIPGISVYVDDIYFSTE
TVSEHLKILEKVFKILLEAGYIVSLKKSALLRYEVTFLGFSITQTGRGLTSEFKDKIQNITSPRTLKELQSILGLFNFAR
NFVPNFSEIIKPLYSLISTAEGNNIKWYLEEIVSALNHAGNLEQRDNESPLVVKLNASPKTGYIRYYNKGGQKPIAYASH
VFTNTELKFTPLEKLLVTMHKALIKAIDLALGQPIEVYSPIISMQKLQKTPLPERKALSTRWITWLSYLEDPRITFYYDK
TLPDLKNVPIIEYAAVFYTDGSAIRSPDKNKSHSSGMGIVHAVFKPELTIEHQWSIPLGDHTAQYAEISAVEFACKKANN
ISGPVLIVTDSDYVARSVNEELPFWRSNGFVNNKKKPLKHISKWKNISDSLLLKRDIIIVHEPGHKPSYTSIHTQGNNLA
DKLATQGSYTVNNIPSLDAELEQLINGSVKGYPSRYKYILKEGQVFVLRPEGKIIPPKSDRLALVKIAHEHAGREATVLR

LQDKYWWPNMRKDVISHIRTCKPCLTTDGSNLTPIPPKPQLRPEKPFDKFFIDYIGPLPPSHGFVYVLVVVDAATGFTWL

YPTKAPSTNATITSLNILLGTAVPRVLHSDQGSAFTSSTFADWAKEKGIQLEFSTPYHPQSSGMVERKNREIKRLITKLL

VGRPTKWYPLLPTIQLALNNTYSVHYKKTPHQLLFGVDGNVPFANQDTLDLTREEELSLLSEVRTSLWLPSVGLLVQERV

ARPSQLRPKWKKPTPILEVVNDRTVVILDNQGQRRTVSIDNLKLTPHQYTCYLACATTTRIMIWILFCVIVLTVALIICF

TTAARIQWRHAIITPGPVIDWNVEVNATGIPQGVILLPHPKPIIQKNRVLGLSQILLINSESLASIFNIKQEKSILTEII

QEEMRSLQDITLNFDLPIGNPKTQHEYIQSRCFQEFKDCYLVKYQNNKPWPTDDVLADMCPLPGWDYYLEISNIRPLRSR

ALPAEWNTQGQNRLFRNLSRIDYCKLPEAVVLLNSTKYDYSLWEGDCHPYTCRHWRYIDFGWLAYNNHFPSPVCVKYEVY

SLYGECIQKLQKAGYAITNAVTQIAKITDLNNEAIVSGIYLLKDHIVTLMEATLHDVSALGNVVTIQHFHTHLAQFKLLL

VENRIDWNYIDSRWIQDQLGLDEADMKILRRTARALIYNVEEIDFRPTSTTWEIALYYEIIVPGKVYSTNWEVHNIGHLV

DSAGSLTLVTIQHPYTIVNQECGETKYLHMEECTEQDYKICEQVTEVLPCGNLTGSDCPVLAKTVKPGYVHIESLRNGSY

IYMAHYQDCGIKPYVPQIVTVNATVKCLGYEIQPPLPQVPSLKLRLPHLVGILAKLKNIQIQVTSTWESIKDQVEKSQTE

LLRLDIHEGDTPAWIKQLAESTKDIWPTTANIFGKVGEFLSGTFGGLFGT----LGYIKPIILGIVILLLIVIVVKIISW

L

>SFVssc

PKPQKQYHINPKAKPSIQIVINDLLKQGVLKQQNSIMNTPIYPVPKTEGKWRMVLDYRAVNKTIPLIAAQNQHSAGILTN

LVRQKYKSTIDLSNGFWAHPIDQDSQWITAFTWEGKQYVWTRLPQGFLNSPALFTADVVDLLKEIPNVNVYVDDIYVSTE

TINQHFQVLDKIFQKLLQAGYVVSLKKSNLCRYEVTFLGFTISKYGRGLTEEFQEKLRNISPPNSLKQLQSILGLLNFAR

NFIPNFSELIKPLYELISTAQGQSISWALNNLIIALNHADNLEQRNGEVPLVIKINASNTTGYIRFYNKNGKRPIAYASH
VFNHTEQKFTPVEKLLTTMHKAIIKGIDLAIGQPIEIYSPIVSMQKLQKITLPERKALSTRWLSWLSYIEDPRFLFIYDK
TLPDLKEMPLHQYLAVFYTDGSSIKSPDPTKTHSSGMGIVQAIYEPNFQIKHQWSIPLGDHTAQYAEIAAVEFACKKALQ
VTGPVLIVTDSDYVARSVNNELNFWRSNGFVNNKKKPLKHISKWKSISESLLLHKNITIVHEPGHQPSSTSVHTQGNALA
DKLAVQGSYTINNIPSLDTELRAVLEGLPKGYPKNLKYEYNSPNLIVIRKEGRIIPPLSDRPKLVKQAHEHTGREATLLR
LQNQYWWPKMRKDVSHCLRTCMPCLQTNSTNLTTTRPFQQIRPSKPFDKYYIDYIGPLPPSEGYSYVLVVVDSATGFCWL
YPTKAPSTRATVKSLNFLLGIAVPKILHSDQGSAFTSSDFANWAKEKEITLEFSTPYHPQSSGKVERKNQEIKKLLTKLL
VGRPAKWYPLIPSVQLALNNTYSPKIKLTPHQLLFGVDGNIPFANSDTLDLKREEELALLSEIRTTLWTPSVGLLVQERV
YRPSQLRPKWKKPTPILEVLNERTVVI-DNNGQRRTVSVDNLKYTPHQYMCYLACATTTRIMGWIIFTLIIASVILVTCF
VVMARIQWRNAITVPGIILDWNVEINTTSLPQGILFEPHPKPIIGKERVLGLSQVILINSESIATSLEIKQEKHILVEMI
KEELLSLQNVMLNFDLPLGDPKTQQEYISQRCFQEFKHCYLVAYNTQKPWPTDDVVQDMCPLPGWDYYLEIKNIRPLKAR
ALSAYWHPQGQNKLFRNITRLDYCKYPEAVILLNTTKSDYSLWEGDCHPYTCRHWRFKDFGWLAYQGHFPSPICEKYTVY
SLFGECLNKLQAVGFTITNTVSKIARIIDLNNEHLVSGLYLLKDHLVTLMESTLHDISILGNAVAIQHFHTHLTQLKLLL
MENRMDWTFIDSSWIQDQLKLSDEDMKILRRASRALVYKVEEIGEGVTSTIWEIGIYYEIIIPRVIYSTNWKIMNLGHLV
YSADNLVQINVEQPYEILNVECGKSTYLHIDKCEEQDYVICEVIQEKQPCGNQSGSDCPVKARTIEKGYTYIQPLKNGSY
VVMSHFQDCHIKPYIPQIVTVNATVKCLGEVFQPPLPVTTSLKLQLPHLVGIITKLKGFQVQITSTWESIKGQVEQAQAE
LLRLDLHEGDSGQWIKQLASASKDIWPAAATVLGKIGDFLGGTAGSIFG----IFGYLKPIFIGLTILILIVLVFKILSW

L

>SFVppy

PRPQKQYPINPKAKESIQIVINDLLKQGVLIQQNSIMNTPVYPVPKPDGRWRMVLDYREVNKTIPLIAAQNQHSAGILAS

IYRGTYKTTLDLANGFWAHPITPNSYWLTAFTWQGKQHCWTRLPQGFLNSPALFTADVVDLMKHIPNVQVYVDDLYLSHD

DPQEHLQVLQQVLHILHDAGYVVSLKKSAIAQKVVEFLGFNITKTGRGLTDAFKEKLLNISPPQNLKQLQSILGLMNFAR

NFIPNYAERVKPFYSLISTAKSNNILWQLQELITLLNQADNLEERKPTTRLIIKVNSSSHAGYIRYYNEGSKKPILYINY

VFSKAEEKFSMLEKLLTTLHKALIKAVDLAMGTEIMVYSPIVSMTKIQKTPLPERKALPVRWITWMTYLEDPRITFHYDK

TLPELKDVPPSQYSMVFYTDGSAIKNPNPTKTHSAGMGVVQGKFNPEFQVVNQWSIPLGNHTAQLAEVAAVEFACKQALK

ITGPVLIITDSFYVAESANKELPYWKSNGFVNNKKKPLKHVSKWKSIADCLSLKTGITIKHEKGHQPSHTSVHTEGNALA

DKLATQGSYVVNNIPSLDAELDQVLQGLPKGYPKHFVYTLEEGKVIVKRPEGKIIPPLADRKLLASQAHKHSGREATLLK

LSNTYWWPNMRKDVVKVIGQCQQCLVTNPSNLTSGPILRPERPTKPFDKFFIDYIGPLPPSNGYLHVLVVVDAMTGFVWL

YPTKAPSANATVKALNMLTSIAVPKVIHSDQGAAFTSSTFADWAKEKGIHLEYSTPYHPQSSGKVERKNSDIKRLLTKLL

VGRPTKWYDLLSTVQLALNNAYSPILKHTPHQLLFGVDANIPFANQDTLDLTREEELSLLQEIRESLWTPAVGLLVQERV

ARPASLRPRWHKPVKILEVLNPRTVVILDHLGNNRTVSVDNLKLTANQYLLYTCCATTTRTLAWLFLFCVLLIVVLVTCF

ITIARIQWNQDIQVYGPVIDWNVEVNMTSIPQGVYYEPHPEPIIVKERVLGLSQVIMINSETVANSANLTQEKVLLADMV

NEELQGLADVMIDFEIPLGDPRDQDQYIHRKCFQEFAHCYLVKYKDPKGWPSEKLIVDQCPIPGWDYYIPFQMMRPLKER

ALPPSWSTEGKSLLFREANTLDICNIPEAILLLNTTYYNFSLWEGDCHPYACRFWRYKDFGFLAYLGHFPSPICIKYSVY

SLYQECINKLRSMGYSLTGAVQTLSKISDINDENLQQGLYLLRDHLVTLMEATLHDISLMEGMLAVQHLHTHLNHFKTML

LERRIDWTFINSDWLQQQLQQPTDHMKIIKRTARSLVYYVEQTSNSPTATSWEVGIYYEIIIPKHIYLNNWQIKNIGHLI

HSAGQLTHVTIDHPYEILNRECEETKYLHLEQCIKQDYVICDIVERVQPCGNTTGTDCAVYAKAIKSPYTEILPLKNGSY

LVLSDSTSCNILPYIPSIVTVNETVECFGVLFKKPLPHIPPLRLRLPHLLGIIAKLKNIKIEVTSTQENIKDQIERAKAE

LLRLDIHEGDSPAWIKQLAAATEDVWPTLATGLKSIGNFLSDAAQGIFGTAFGILGYVKPILIGVGIILLIVVIFKIISW

I

>SFVmcy

PRPQKQYPINPKAKPSIQIVIDDLLKQGVLIQQNSTMNTPVYPVPKPDGKWRMVLDYREVNKTIPLIAAQNQHSAGILSS

IYRGKYKTTLDLTNGFWAHPITPESYWLTAFTWQGKQYCWTRLPQGFLNSPALFTADVVDLLKEIPNVQAYVDDIYISHD

DPQEHLEQLEKIFSILLNAGYVVSLKKSEIAQREVEFLGFNITKEGRGLTDTFKQKLLNITPPKDLKQLQSILGLLNFAR

NFIPNYSELVKPLYTIVANANGKFISWQLQHIISVLNQADNLEERNPETRLIIKVNSSPSAGYIRYYNEGSKRPIMYVNY

IFSKAEAKFTQTEKLLTTMHKGLIKAMDLAMGQEILVYSPIVSMTKIQRTPLPERKALPVRWITWMTYLEDPRIQFHYDK

SLPELQQIPPSEFAMVFYTDGSAIKHPDVNKSHSAGMGIAQVQFIPEYKIVHQWSIPLGDHTAQLAEIAAVEFACKKALK

ISGPVLIVTDSFYVAESANKELPYWKSNGFLNNKKKPLRHVSKWKSIAECLQLKPDIIIMHEKGHQQPMTTLHTEGNNLA

DKLATQGSYVVHCNPSLDAELDQLLQGYPPGYPKQYKYTLEENKLIVERPNGRIVPPKADREKIISTAHNHTGRDATFLK

VSSKYWWPNLRKDVVKSIRQCKQCLVTNATNLTSPPILRPVKPLKPFDKFYIDYIGPLPPSNGYLHVLVVVDSMTGFVWL

YPTKAPSTSATVKALNMLTSIAIPKVLHSDQGAAFTSSTFADWAKEKGIQLEFSTPYHPQSSGKVERKNSDIKRLLTKLL

IGRPAKWYDLLPVVQLALNNSYSPSSKYTPHQLLFGVDSNTPFANSDTLDLSREEELSLLQEIRSSLWSPSVGQLVQERV

ARPASLRPRWHKPTAILEVVNPRTVIILDHLGNRRTVSVDNLKLTAYQYLAYSCCATSTRVLCWIVLVCVLLLVVFISCF

VTMSRIQWNKDIAVFGPVIDWNVEVNMTSIPQGVLYVPHPEPIILKERILGLSQVMMINSENIANTANLTQEKVLLADMI

NEEMNDLANQMIDFEIPLGDPRDQKQYQHQKCFQEFAHCYLVKYKTTKGWPSSTVIADQCPLPGWDYYVPFEQIRPLKDR

AMPAEWDKQGKADLFRQINTLDVCNRPEMVFLLNSSYYEFSLWEGDCHPYSCRFWRYKDFGFLAYLNSFPSPICIKYKIS

SLYLECMNRLRSMGYALTGAVQTLSQISDINDERLQHGVYLLRDHVVTLMEAALHDVSIMEGMLAIQHVHTHLNHLKTML

LMRKIDWTFIRSDWIQQQLQKTDDEMKLIRRTARSLVYYVTQTSSSPTATSWEIGIYYEIVIPKHIYLNNWQVINVGHLL

ESAGHLTHVKVKHPYEIINKECSDTQYLHLEECIREDYVICDIVQIVQPCGNATESDCPVTALKVKTPYIQVSPLKNGSY

LVLSSTKDCSIPAYVPSVVTVNETVKCFGVEFHKPLPQVPHLKLRLPHLTGIIASLQSLEIEVTSTQENIKDQIERAKAQ

LLRLDIHEGDFPDWLKQVASATRDVWPAAASFIQGVGNFLSNTAQGIFGSAVSLLFYAKPILIGIGVILLIALLFKIISW

L

>SFVcae

PRPQKQYPINPKAKASIQTVINDLLKQGVLIQQNSIMNTPVYPVPKPDGKWRMVLDYREVNKTIPLIAAQNQHSAGILSS

IFRGKYKTTLDLSNGFWAHSITPESYWLTAFTWLGQQYCWTRLPQGFLNSPALFTADVVDLLKEVPNVQVYVDDIYISHD

DPREHLEQLEKVFSLLLNAGYVVSLKKSEIAQHEVEFLGFNITKEGRGLTETFKQKLLNITPPRDLKQLQSILGLLNFAR

NFIPNFSELVKPLYNIIATANGKYITWQLQNIISMLNSAENLEERNPEVRLIMKVNTSPSAGYIRFYNEFAKRPIMYLNY

VYTKAEVKFTNTEKLLTTIHKGLIKALDLGMGQEILVYSPIVSMTKIQKTPLPERKALPIRWITWMSYLEDPRIQFHYDK

TLPELQQVPPSEFSMVFYTDGSAIKHPNVNKSHNAGMGIAQVQFKPEFTVINTWSIPLGDHTAQLAEVAAVEFACKKALK

IDGPVLIVTDSFYVAESVNKELPYWQSNGFFNNKKKPLKHVSKWKSIADCIQLKPDIIIIHEKGHQPTASTFHTEGNNLA

DKLATQGSYVVNINPSLDAELDQLLQGYPKGFPKHYQYQLENGQVMVTRPNGRIIPPKSDRPQIILQAHNHTGRDSTFLK

VSSKYWWPNLRKDVVKVIRQCKQCLVTNAATLAAPPILRPERPVKPFDKFFIDYIGPLPPSNGYLHVLVVVDSMTGFVWL

YPTKAPSTSATVKALNMLTSIAVPKVIHSDQGAAFTSATFADWAKNKGIQLEFSTPYHPQSSGKVERKNSDIKRLLTKLL

VGRPAKWYDLLPVVQLALNNSYSPSSKYTPHQLLFGIDSNTPFANSDTLDLSREEELSLLQEIRSSLWSPSVGQLVQERV

ARPASLRPRWHKPTPVLEVINPRAVVILDHLGNRRTVSVDNLKLTAYQYLAYACCATSTRVMCWLFLICVLLIIVFVSCF

VTVARIQWNRDINVFGPVIDWNISINMSSIPQGVMYTPHPEPIILKERVLGISQVLMINSENIANVANLSQEKVLLTDMI

NEELQDLSNQMIDFELPLGDPRDQDQYIHHKCYQEFAHCYLVKYKKPSPWISEGIIVDQCPLPRWDYYLKIQNIRPLKAR

ALPKEWNNQGNARLFRSFNPLDVCNRPEAVLLLNTTYFTYSLWEGDCHPYACRFWRYKDFGFLSYLNAFPGLKYIEYEVY

SLYMECMNKLRSMGYSLTGAVQTLSQISDINDERLQQGVSLLRDHVVTLMEAALHDITIMEGMLAIQHVHTHLNHLKTIL

LMRKIDWTFIKSNWIKEQLQKTEDEMKIIRRTAKSLVYYVTQTSSSTTATSWEIGIYYEITIPKHIYLNNWQVINIGHLV

ESAGHLTLIRVKHPYEVINKECTYEQYLHLEDCISQDYVICDTVQIVSPCGNSTTSDCPVTAEKVKEPYVQVSALKNGSY

LVLTSRTDCSIPAYVPSIVTVNETVKCFGVEFHKPLPQVPHLKLRLPHLVGIIANLQNLEIEVTSTQESIKDQIERAKSQ

LLRLDIHEGDFPAWIQQLASATRDVWPAAARALQGIGNVLSNTAQGIFGTTVSILSYAKPILIGIGVILLIAFLFKIVSW

L

>SFVpve

PRPQKQYPINPKAKPSIQIVIDDLLKQGVLTPQNSTMNTPVYPVPKPDGRWRMVLDYREVNKTIPLTAAQNQHSAGILAT

IVRQKYKTTLDLANGFWAHPITPDSYWLTAFTWQGKQYCWTRLPQGFLNSPALFTADAVDLLKEVPNVQVYVDDIYLSHD

NPHEHIQQLEKVFQILLQAGYVVSLKKSEIGQRTVEFLGFNITKEGRGLTDTFKTKLLNVTPPKDLKQLQSILGLLNFAR

NFIPNFAELVQTLYNLIASSKGKYIEWQLNKVIEALNTASNLEERLPDQRLVIKVNTSPSAGYVRYYNESGKKPIMYLNY

VFSKAELKFSMLEKLLTTMHKALIKAMDLAMGQEILVYSPIVSMTKIQKTPLPERKALPIRWITWMTYLEDPRIQFHYDK

TLPELKHIPPSQYEGVFCTDGSAIKSPDPTKSNNAGMGIVHAIYNPEYKILNQWSIPLGHHTAQMAEIAAVEFACKKALK

VPGPVLVITDSFYVAESANKELPYWKSNGFVNNKKEPLKHISKWKSIAECLSIKPDITIQHEKGHQPINTSIHTEGNALA

DKLATQGSYVVNCNPNLDAELDQLLQGNVKGYPKQYTYYLEDGKVKVSRPEGKIIPPQSDRQKIVLQAHNHTGREATLLK

IANLYWWPNMRKDVVKQLGRCKQCLITNASNKTSGPILRPDRPQKPFDKFFIDYIGPLPPSQGYLYVLVIVDGMTGFTWL

YPTKAPSTSATVKSLNVLTSIAIPKVIHSDQGAAFTSSTFAEWAKERGIHLEFSTPYHPQSSGKVERKNSDIKRLLTKLL

VGRPTKWYDLLPVVQLALNNTYSPVLKYTPHQLLFGIDSNTPFANQDTLDLTREEELSLLQEIRASLWSPVVGQLVQERV

ARPASLRPRWHKPSTVLEVLNPRTVVILDHLGNNRTVSIDNLKPTSHQYLLYTCCATSSRVLAWMLLVCVLLIVVLVSCF

LTISRIQWNRDIQVLGPVIDWNIEVNMTSIPQGVYYEPHPEPIVVTERVLGLSQVLMINSENIANNANLTQEKKLLAEVV

NEEMQSLSDVMIDFEIPLGDPRDQEQYIHRKCYQEFAHCYLVKYKTPKSWPTEGLIADQCPLPGWDYYIKVEITRPLKKR

ALPKEWSSQGKNALFKEINVLDVCSKPELVILLNTSYYSFSLWEGDCHPYACRFWRSKDFGFLAYQKNFPAPICIEYEVY

SLYQECKLKLKSMGYALTGAVQTLSQISDINDENLQQGIYLLRDHVITLMEATLHDISVMEGMFAVQHLHTHLNHLKTML

LERRIDWTYMSSAWLQQQLQKSDDEMKVIKRIAKSLVYYVKQTYNSPTATAWEIGLYYELTIPKHVYLNNWNVVNIGHLV

QSAGQLTHVTIAHPYEIINKECTETKYLHLKDCRRQDYVICDVVEIVQPCGNSTDSDCPVWAEAVKEPFVQVNPLKNGSY

LVLASSTDCQIPPYVPSIVTVNETTSCYGLNFKKPLPRLPNLQLRLPHLVGIIAKIKGLKIEVTSSGESIKDQIERAKAE

LLRLDIHEGDTPAWIQQLAAATKDVWPAAASALQGIGNFLSGAAHGIFGTAFSLLGYLKPILIGVGVILLIILIFKIVSW

I

>SFVggo

PRPQKQYPINPKARSSIQVVIDDLLKQGVLVQQNSTMNTPVYPIPKPDGRWGMVLDYREVNKTIPLIAAQNQHSAGILAT

IVRKKYKTTLVLANGFWAHPITPESYWLTAFIWQGKQYCWTRLPQGFLNSPALFTADVVDLLKEISNVQAYVDDIYLSHD

DPQEHLDQLEKVFQILLQAGYVVSLKKSEVAQKTVEFLGFNITKEGRGLTEAFKAKLLDITPPKDLKQLQSILGLLNFAR

NFILNFAELVKPLYSLISSAKGKYIEWQLQTIIKALNNADNLEERIPEKRLIIKVNTSPSAGYVRYYNETGKKPIMYLNY

VFSKAELKFTLLEKLLTTMHKALIKAMDLAMGQEILVYSPVVSMTKIQKTPIPERKALPIRWITWMTYLEDPRIQFHYDK

TLPELKNIPPSQYNSVFYTDGSAIRSPDPTKSHNAGMGIVQVKFSPELQVINQWSIPLGNHTAQMAEIAAVEFACKKALK

ITGPVLIITDSFYVAESTNKELPYWKSNGFVNNKKKPLKHVSKWKSIAECLSLKPDITIQHERGHQPIYTSIHTEGNALA

DKLATQGSYVVNNNPNLDAELDHLIQGYPKGYPKQYTYYMEDGKVKVNRPEGKIIPPSLERAGIVQKAHNHTGREATLLK

IANLYWWPNMRKDVVRQLGRCQQCLVTNAFNQTSGPILRPTRPLKPFDKFFIDYIGPLPPSNGYLHVLVVVDSMTGFTWL

YPTKAPTTNATVKALNVLTSIAVPKVIHSDQGAAFTSSTFADWAKERGIQLEFSTPYHPQSSGKVERKNSDIKRLLTKLL

VGRPTKWYDLLPVVQLALNNSYSPSLKHTPHQLLFGIDSNTPFANQDTLDLTREEELSLLQEIRSSLWSPIGGQIVQERV

PRPASLRPRWHKPSRIVDILNERTVVIVDHLGNNRTVSIDNLKLTPHQYFLYTCCATSSRVLAWMLLACILFIIIIVSCF

ITLSRIQWNKDIQVLGPVIDWNVEVNMTSIPQGVFYQPHPEPIIHTERVLGLSQVLMINSENVANSANLSQEKVLLTEMI

NEEMQSLSDVMIDFEIPLGDPRDQEQYIHRKCYQEFAHCYLVKYKTPQPWPNEGLIVDQCPLPGWDYYAKIENIRPLKNR

ALPYEWAKGGLNRLFRNISVLDVCSRPEMVLLLNKTYYTFSLWEGDCHPYSCRLWRYKDFGFLSYMKNFPGPQCIEYEVY

SLYQECKLKLRSMGYALTGAVQTLAQISDINDQNLQQGIYLLRDHIVTLMEATLHDISIMEGMFAVQHVHTHLNHLRTML

MERRIDWTYMSSSWLQTQLQKSDDEMKVIKRTARSLVYYVKQTYNSLTATAWEIGLYYELIIPRHIYLNNWQVVNIGHLI

KSAGQLTHVTVSHPYEIINRECSNTLYLHLEECRRLDYVICDVVKIVQPCGNSSDSDCPVWAEPVKEPHVQISPLKSGSY

LVLASSTDCQIPPYVPSVVTVNETTQCFGVTFKKPLPQLPHLQLRLPHLVGIIAKIKGIKIEVTSSGESIKDQLERAKAE

LLRLDIHEGDTPAWIRQLAAATEDVWPAAASALKGIGNFLTGAAQGLFGTAFSILGYLKPILIGIGIIILVILIFKILRW

I

>FFVfca

PTPQKQYHINPKAKPDIQIVINDLLKQGVLIQKESTMNTPVYPVPKPNGRWRMVLDYRAVNKVTPLIAVQNQHSYGILGS

LFKGRYKTTIDLSNGFWAHPIVPEDYWITAFTWQGKQYCWTVLPQGFLNSPGLFTGDVVDLLQGIPNVEVYVDDVYISHD

SEKEHLEYLDILFNRLKEAGYIISLKKSNIANSIVDFLGFQITNEGRGLTDTFKEKLENITAPTTLKQLQSILGLLNFAR

NFIPDFTELIAPLYALIPKSTKNYVPWTLETLITKLNGAEYLQGRKGDKTLIMKVNASYTTGYIRYYNEGEKKPISYVSI

VFSKTELKFTELEKLLTTVHKGLLKALDLSMGQNIHVYSPIVSMQNIQKTPQTAKKALASRWLSWLSYLEDPRIRFFYDP

QMPALKDLP-SNFQHIFYTDGSAITSPTKEGHLNAGMGIVYINKDGNLQKQQEWSISLGNHTAQFAEIAAFEFALKKCLP

LGGNILVVTDSNYVAKAYNEELDVWASNGFVNNRKKPLKHISKWKSVADLKRLRPDVVVTHEPGHQKLDSSPHAYGNNLA

DQLATQASFKVHMTPKLDIEQIKAIQALPVGYPKQYTYELQNNKCMVLRKDGREIPPSRERYKLIKEAHNHAGREAVLLK

IQENYWWPKMKKDISSFLSTCNVCKMVNPLNLKPISPQAIVHPTKPFDKFYMDYIGPLPPSEGYVHVLVVVDAATGFTWL

YPTKAQTSKATIKVLNHLTGLAIPKVLHSDQGSAFTSEEFAQWAKERNIQLEFSTPYHPQSSGKVERKNSEIKKLLTKLL

VGRPLKWYNLISSVQLALNNTHVVSTKYTPHQLMFGIDCNLPFANKDTLDWTREEELALLQEIRESLWSPYVGQLVQERV

YRPSQLRPKWRKPTKVLEILNPRTVIIVDHLGQRKSVSIDNLKPTAHQYRCYTLCATSTRIMFWILFFLLCFSIVTLSTI

ISILRYQWKEAITHPGPVLSWQVEVNISGIPQGLFFAPQPKPIFHKERTLGLSQVILIDSDTITQG--HIKQKAYLVSTI

NEEMEQLQKTVLPFDLPIKDPLTQKEYIEKRCFQKYGHCYVIAFNGNKVWPSQDLIQDQCPLPPWKYYIPLPFKVSLRDI

AFPKELSPVGSGMLFRPINPYDICNMPRAVLLLNKTYYTFSLWEGDCHPYACRFWRNKDWGWLAYTDSFPSPICIEYTLS

SVLAECVNRIQEAGLGLANAITTVAKISDLNDQKLAKGVHLLRDHVVTLMEANLDDIVSLGEGIQIEHIHNHLTSLKLLT

LENRIDWRFINDSWIQEELGVSDNIMKVIRKTARCIPYNVKQTRNLNTSTAWEIYLYYEIIIPTTIYTQNWNIKNLGHLV

RNAGYLSKVWIQQPFEVLNQECGTNIYLHMEECVDQDYIICEEVMELPPCGNGTGSDCPVLTKPLTDEYLEIEPLKNGSY

LVLSSTTDCGIPAYVPVVITVNDTISCFDKEFKRPLPSVPQLELRVPRLTSLIAKIKGIQIEITSSWETIKEQVARAKAE

LLRLDLHEGDYPEWLQLLGEATKDVWPTISNFVSGIGNFIKDTAGGIFGTAFSFLGYVKPVLLGFVIIFCIILIIKIIGW

L

>TraEFVtja.a1

SRPQRQYHINPKAKPDMQIVVNDLLKQGVLIQKESSMNTPVYLVPKSNGRWRMVLDYREVNKVTPLIATQNQHSAGILSS

LYRGKYKSTIDLANGFWAHPITRESYWLTAFAWSGKQYCWMVLPQDFI----ALLCSLRMLMYEITNVQVYVDDVYLSND

TIEEHLDALRKMFQLLREAGYIVSLQKSALCQSTIEFLGFQITSTGRGWTSSFTEKLVHLKPPTTLKDLQSILGFMNFAR

NFLPNFAELIAPLYRLIPEFKISVVPWLLTKLIKALNDAAELQERDPTLKLILKCNACPTTGYARFYNEKGISPIQYLSI

LF--TELKFSILEKLLTTIHKALLKGLDLAMGQGIMVYSPVVSMTKIQKTLITARKALASRWVTWMTYLEDPRIEFYYDN

TLP-LKLLPLTEYASEFYTDGSAIKSPDKTKTYCSGMGIVQYKYTPEPKIIKEWSVPLGDHTAQFAEIAT-EFACKQALK

IQGPILIMTDSDYLAKSYNNELSYWISNRFVNNRKKPLTHIGKWKNIADCLKIKPDIFVAHEPGHQETNVSLHAKVNGLA

DTLAIHASYTVNTVPELDSELNQLLSGTIKGSPTQFKYELQGMDVVTIRPEGRTVPPRSNRAKIIKEALSLGGQNTTMMT

LQQKFWWPNMRKDVVKYLRLCEICAPVNPSNIKPSRPILQTRPPKPFERFQMDLIGPLPTSEGYSSVLVIVDVASGYIWL

YPTKAQTTKGTIKALNLLTGTAIPKVLHSDQGAAFISSDLATWAKEKNITLEFSTPYHPQSSGKVERENSEIKRLLTKLL

VGWPMKWYNLIPDTQFALNNTPSTVTKYIPHQLMFGIGCNQPFANRDTFNWTREEQLALLAEIRQSLWLPTVGQLIQESV

YRPSQLRPKWRKPTPIVAVLNNHTVQIVDHLGQPKIVSVDNLKLTPHQHWLHSSRAILTRILGWVFLVVLFLAIITTTII

TTLFRLQWKAAIETPGPVINWTV------LSQGIFFEPHP-PILAKERVFAISQLIMINTETIIAETDLTTAAEYLTQMV

NEEMRNLKSLILPFDLPIGDPTSQ-EYIRKKCFQDLGHCYLVDFGTERQWPDSTFIQDQGPIPGWEFYVSSNASGLLKAD

LLSPNWHYNGENKLFQNVTLTDSCTRPLLPILLNSTRYEWSLWEGDCHPYACLHWRYRDFGYLASLNIFPSPMCKTDNVY

SIYAECVNKVQTTVLGLVSAINTVARLSDLNDQNLASGIHLLKDHIITPMEGTIADVSLVTGAIQVEHFHNHISQFKHTL

MNN-VDWNIMDSAWIQEELKLSEEIMRIMRRTANSITYRVEQTRNLLNASAWKIYVVYELTTPKEVYTQHWEIKNLGHLV

RNSGYLTIVWVEHPYQILNRDCEKTRFLQLKDCYIKDYLIYEEVKEVQLCGNQTGKNCLVKASKISEPWVLIQPLKNGSN

LLMTDSTDCGIEPYVSALIIVNDS-------FKRALPQVPPLRIRLPHLLGIIAKLKNFEVTITSTAEAINDQLERTKNE

LLRLDIHEGDLPHWIQQLATATKDIWPAAAHAIQGIGNFVSKVIGGTFGSAINIMVYLRP-------------------

-

>TraEFVtka.a2

SRPQRQYHINPKAKPDMQIVVNDLLKQGVLIQKESSMNTPVYLVPKSNGRWRMVLDYREVNKVTPLIATQNQHSAGILSS

LYRGKYKSTIDLANGFWAHPITRESYWLTAFAWSGKQYCWMVLPQDFI----ALLCSLRMLMYEITNVQVYVDDVYLSND

TIEEHLDALRKMFQLLREAGYIVSLQKSALCQSTIEFLGFQITSTGRGWTSSFTEKLVHLKPPTTLKELQSILGFMNFAR

NFLPNFAELIAPLYRLIPEFKISVVPWLLTKLIKALNDAAELQERDPTLKLILKCNACPTTGYARFYNEKGISPIQYLSI

LF--TELKFSILEKLLTTIHKALLKGLDLAMGQGIMVYSPVVSMTKIQKTLITARKALASRWVTWMTYLEDPRIEFYYDN

TLP-LKLLPLTEYASEFYTDGSAIKSPDKTKTYCSGMGIVQYKYTPEPKIIKEWSVPLGDHTAQFAEIAT-EFACKQALK

IQGPILIMTDSDYLAKSYNNELSYWISNRFVNNRKKPLTHIGKWKNIADCLKIKPDIFVAHEPGHQETNVSLHAKVNGLA

DTLAIHASYTV---PELDSELNQLLSGTIKGSPTQFKYELQGMDVVTIRPEGRTVPPRSNRAKIIKEAFSLGGQNTTMMT

LQQKFWWPNMRKDVVKYLRLCEICAPVNPSNIKPSRPILQTRPPKPFERFQMDFIGPLPTSEGYSSVLVIVDVASGYIWL

YPTKAQTTKGTIKALNLLTGTAIPKVLHSDQGAAFISSNLATWAKEKNITLEFSTPYHPQSSGKVERKNSEIKRLLTKLL

VGWPMKWYNLIPDIQFALNNTPSTVTKYIPHQLMFGIGCNQPFANRDTFNWTREEQLALLAEIRQSLWLPTVGQLIQESV

YRPSQLRPKWRKPTPIVAVLNNHTVQIVDHLGQPKIVSVDNLKLTPHQHWLHSSRAILTRILGWVFLVVLFLAIITTTII

TTLFRLQWKAAIETPGPVINWTVNVTTHPLSQGIFFEPHP-PILAKERVFAISQVIMINTETIIAETDLTTAAEYLTQMV

NEEMRNLKSLILPFDLPIGDPTSQ-EYIRKKCFQDLGHCYLVDFGTERQWPDSTFIQDQGPIPGWEFYVSSNASGLLKAD

LLSPNWHYNGENKLFQNVTLTDSCTRPLLPILLNSTRYEWSLWEGDCHPYACLHWRYRDFGYLASY--FPSPMCKT----

--------KVQTTVLGLVSAINTVARLSDLNDQNLASGIHLLKDHIITPMEATIADVSLVTGAIQVEHFHNHISQFKHTL

MNN-VDWNIMDSAWIQEELKLSEEIMRIMRRTANSITYRVEQTRNLLNASAWKIYVYYELTTPKEVYTQHWEIKNLGHLV

RNSGYLTIVWVEHPYQILNRDCEKTRFLQLKDCYIKDYLIYEEVKEVQLCGNQTGKNCLVKASKISEPWVLIQPLKNGSD

LLMTDSTDCGIEPYVSALIIVNDS-------FKRALPQVPPLRIRLPHLLGIIAKLKNFEVTITSTAEAINDQLERTKNE

LLRLDIHEGDLPHWIQQLATATKDIWPAAAHAIQGIGNFVSKVIGGTFGSAINIMVYLRP--------------------

-

>sloEFV

PKPQKQYHINYKAKLAIQTVINDLIKQGVLLHQNSSMNTPIYPVPKTNGSWRMVLNFRAVNKVIPLIAVQNQYSIEILTQ

MQREQYKTTLDLSNGFWAHPIRKESYWLMAFTWEGKQLVWTRLPQGFINSPALFTANIVDILKEIPDVEVYVNDIYFSNV

TEEQHLITLKQVLKILLKSGYIVSLKKSEIAKEEVTFLSFNITKEGHGLTAKFREKLLNISAPKTLKQLQSILGLLNFAH

NVITDFAELTKPLYLVISRAEGQHIQWALQEIIKKLNNASYLENRDIQKPLIIKLNSSPTAGYIRMYNKGGKKPIQYVNF

IFTPAEIKFKPTEKLLTTMHKAIIKGLDLSQGAQVHIYSPLASPTHIQKTPLPERKGLHSQWITWMTHFKNPQLIFHHDP

TLPDIQNLPLTSYTAVYYTDGSAIKNPNPQKTHSAGIGIVKGKFDPNFSIIKQWRFPLGDHTAQYAEISALEFAVKKAMM

DKGPILIVTNSMYLAKSFNEELDIWISNGFVNNKKKPLQHISKWKVIANCKQNKPSIHMVHEPGHQKQGTSIHTKGNLLA

DQLAVQSSHMVGMVPSLDKELEQVLDSNPKGYPVKYIYLLENGNVIIEQDEGRIIPPVMERVKLAQQAHNHGGWEATLIK

LKNKYWWPNMIKTVRSVVANCEKCQVTNASSQIPTPPKTIIHPDKPFEKFYMDYIGPLPSSHGHKHILVVDDARMGYCWL

FPTKAQNANATVKALNFLSGTAIPKVLHSDQGSAFTSATLQQWTKDRGIQLEFSTPYHPQSSGKVERKNGKIKRVLTKLL

YGWPQKWYPLIPFVQLSINNIPSSQTHQTPHKLMFGVDSNLPFANVDDANLSREEQLSLLQELREELWKPFIGQFIQERV

QKYTPLCPRWKKPTKILTVFDDHTVEILDPLGQRRKVSIDNLKPTAHYYFLYTLCATSTQILCWFFFGLIIIGLILGFIL

SAVFRLQWKNAIHHPGPIISWNVHLEICGLQQGMFWEQFPKPIIHKKRTLGISQILLIDTPLVWY---IPLKKKILTQLI

DNEFAQLQEIVLPFTLPLDQPYTQEQYQQKGCFQEFGHCYLVKY-----WLTSKIIQDHCLIPTWKYYIQPQIMRPLKDC

ALPSEWK---QNNLFKSPTITQFCNHPELIYFLNTTYTTYSLWEGDCHPYTCQFWRQFDWRQLAYSKKFPAPIC--YKVK

SLYQKCIARLEKVSLMMANSTATVSKLSDLNEYLFADGLHILKDHVVTLLEANMKDTQHIDELTTAMLILSYIQNFRIPS

TEGRIDWRILNGTWINEGLNIPHHGMQIVKRMSCSNIYDIKKTIK----SIWEIGIYYQIILPNKVFYTNWQVLNIGHLV

KTGTQLTLTKIHQPYTHIS-ECSELYYLEPKGCEQRDYLICEEINLHQTCGNKT-SKCPVTGKAV--PYLEFIPLKNGSY

VVMSYTIDCNIPPYQSSIFTINDTVTCFEKILKKHL-HIPKIQLRLPHLVGILAKLKKIEVKATDTWASIEEQIEDTKSD

LLRLELHKGDTPEWIKQLGEALEDVWPAAASATKTIASFVSSATKGIFGGIIDILTYTKPIVILIIITILIVLIFRILKW

L

>CmaEFV

PKPQKQFKINPQAIPSIQIVINYLLKQGIPRQETSEMNTPVYPVPKGEGKWRLVLDYRAVNKVTPAIAAQSCHSTGILMQ

LTRKKYKTTLDLSRWVFGPILSPK----IAFTWCGKQHVWTQLPQGFLNSPALFSADVVDLLKEIPDVSVYVNDIYFLHD

TEKEHLKTTRHLHN--IERGQIHSLKKSEIGKREVNFLGFAITNEGRDLTD-YEEKLLNLQPPKTLKQLQSILGFLNFAH

PFISNFAELVKSLHDAIIKANNNESFWALDDLITAIKQAALLTERDPTKPLAVKLHVSPEGYYVRLYNMADRFPFQYTSI

IFK-AEKRFTLTEKLLTVMQYALIKSFDIAQGQMIHVYSPLRCPETLQRHTIPERKVLSSRWLKWMSHIENPQIKFHYDE

ELPDLASLQLTEYKQIYYLYGSATTNEHKRQ---ARMDAVQAIFNPDYQVLNVWSIPLGQHLAQYAEVAALEFALQQIPM

DQTPRLIITDSDYVSKRYNSKLEFWESNGFCNAKGKPLHHISLWKSISELKKIKPWVHVTHEPGH-CIGTSVRRAGNAAA

DSLAKKASMINRVHPTIDTDLGQCIN-NPPGYKK----KLDQGIYWITKPEGFQIPPTTERHMITERAHPHFGRDATLAV

LK-KCWWPYMIQTVQQVLQYCSKCVTYNSANRAPIPHDKRTIPESPFDILFIDYIGPLPKCPGQLYVLVIIDGATSFVWL

YPTTGPTAQATVRALTDFCKIAIPKKIHSDQGPAFTADISKEFAKKYNIQWEYSTPYHPQNSGKVERANGEVKAALTKLS

GSCPGK-YAYILLVQLGFNNRPRPSIKRTPFKLLFGVPMNVEF--NLTSDLSREEQLPLLAEIQYTLWHPLVGLLVQERV

TT--PLRPRWKPPTLIIKVLSDRVVEIVDKKGNLKQGSIDNLKVTPH-YSGYAAYATSTRIAMWIIFTLLILMTMLGVTC

TVIVRLQWKYAIERQGPTITWNVNVPITGLKQGL-YEPFPKPIVAKERVLGISQILILDSDHMAEANNLGHPKEILTQLL

NE---------IPFEIPLDGPQTQQEYMQKKRCHEFAHYYWIDYK-EQKWPESQVIADHCPHHGEDYWVESPFS--LKKD

ALLNDWHDQGKGRWFKPMDDLSFCRHPELTVFLNGTYHKHSCMEGDCHPYACQFYRLIDTGLLLHDHTFPGFWCIEYSLY

SLYQQCLFKIQEAGLIPDSSITKTAKISDLNDK-LAKGLHLLRDHLITFPEHTIDVIQMSQSIIAVM-THSHIQNLRILL

TEGKVDWDTLNSTWIQEQLRVSDEMMTLIRRTARGLAYDIQQRVDKPEKGVWEISLYYEIVIPR-IYSTNWKIINYGHLV

YTGNRGGRVWLKHPCTLITQGCGEVKYLEVRECYEQDYLICDEVIKHEPCGNQTGSRCPIMVEPIVSPYLRIGPLKNGNY

IVTTSLDECSIPPYQPSLITVNETVTCYGYEFKSPLIHMPPLSVSLPHMIGIIADLRQIKIELASSWDSVHDVTERANTE

LLRIDLREGYTPQWLNRLSESIADIWPAAAGAIKGIANGIKDLTSG---------------------------------

-

>PSFVaye

SKPQRQYAINPKARSSIQAVIDDLLKQGVL-TKTSVMNTPVYPVPKPNGQWRLVLDYEVVNKPNPPLTAQNFCSIGLLTT

LPKHKYKTTLDLSSGFEAYPITEESQWI----FLGVMNAWTRLPQGFLNSPSLLIADVTEILKDIPNMVVYTDDI-----

---------KKIFDNLLKSGYIVSVKKSEMAMREVTFLGF--TQEGRGLMETHKEKL-QLKPPKKVYAFTSQGTLFQILL

NLHTPFMISVQ----LVPRFTGNC---NMTLFSQNLTNILLFSQRKNQIFPLLKKCTFPKLLVLDFTIILGKKTISHLNI

VFSKTKLKFKNIEKLLTTIHKPLLKAFDMAQSQNIKIYSPIASLARIQKTPIPERKAFSSRWTICLVYTEDPRISFYFDS

MLLDLSDLPLQDYKYVFIWIDPPLLLKEKRKKHSAGTGIIQATFSPDFTFLKQWQLPLGDHTAQFAELAAFEFAIKKDYY

AQARINCYRQFLYL-----KLLTVRAPNGFLTAKRKPLKHISKWKQIYDIVKCITEIHMIHEPTHQADSATQHSFGNNQA

DRLHKPLIKFLFRYP---GGAKPILDNNPPGYPNKYMCELCNNKVYVTLPKGRKMPPVHLQLQLAQKAHNHGGRNTTLLK

LQNCY-WPNMLKTVKTVLKDCEPCLMVNFNNVKTIPPIKIQHAQNPFDKIYMDYVGPLSPSHRYEHVLVLADASTRFCLL

YATDVQTTDVTLKAPTHFSSVAIPRVLYSDQESAFTSSSLGLWAKDKGVPLEFSTTYHRQSSGKLKRASGKIKA-LTKLL

VGRPRKRYRLTLEVQLGISNIPNQQGK-TPYKPLFGVSPNVFFSSN-------------LQES-----TPQVGLLVQERI

TKPTGLRTRWKKPTPIIEVLSDQVVKILDEQNQ-PTVSTDNLKLTAFQYWAYVICAIISRILIWIMLFLILFSAILVSTL

IAVFTLQWNKAIYALGPIIIWNVDIDI-ALPQGIFMEPYLKPTVAKERVLGLSHRVLVDSSALFQDAKLNDQKFLLVNKI

NEEMRSLQDLVLQFDFPPDHPKTQRQYVEHKCCQCFAHCYVIDYGVDRQWPTKEIIQDHCPLPS---YSQVPLMQPFKNA

ALPPRNMKEGET--FKETPLTDFCNKPEQVLFINSTYYTRSLREGDCHPYVCRHLRLRNFGYLAFHNAFPAPICIEYKLT

CL------------------------------------------------------------------------------

----------------------------------------------------------------------------------------------

-----------------------------------------------------------------KMC--------------------------

----------------------------------------------------------------------------------------------

----------------------------------------------------------------------------------------------

-

>ChrEFV

PIKQKQNHINHKATPSIQ-VIDNLLAQQ-LKKQTSPMNTPVYPVPKSEGKWRMVLDYRAINKVIEPIAAQNSFSTSILAQ

LPKKKFKTTLDLSNIF-AIPIHPNDYWITAFT-KSLHHVWTRLPQSFINIPALFPADIQQLVLYLMSHMMTSISLQIQSN

NSYNK--------SYISEMQDVSSLPKSQ-PLGDIQFLSV-LKSQNKAEAQKISKLNLNIKEPGSLKQLQSVLGLFNFAR

NFVPDFAVLTELLCKLISTAKGQHTQWALSTLINSVNKTKYLAE--NVKLPLVKCYASQMAGFAGLHNESETTPISFLSF

IFTPAETKFAPTERILTIIHKTLLKASDLAVSI-IKIYSPVTPPTKLQKIPLSERNALNSKWITWPSHFKNPQL-FIYDP

NLPDLSHLPLTEYTYVYYTDGSAIKSSKINFQYSSWTGIIKGKFTPSYTEVNKWSFSAGDHPAQYAEVTVFIFAYKDASK

QVMPVLIVTDSDYVFKAYTKESQIRISNGFHNAKHKPIKRIVKWQQITSLYV--PKSHVIHEPGHQTPDPTKATTGNTIA

DPLATKASREVNVIPTENKDLKQVKDLGNSKYTVKYRYKTINDKPMILLPEGEILP-YDQHFFYAIQTHQDLG-ESTITK

LS-KNIWPSLRLTVDSVLGSCETCLNTHSANVSP-PPFKLDKPENPFDKIYMDYVSPLPSSQEYLHLLVLVDYLTGF-FD

TPHQRSTANTMVHALYVFISVAIPKVTHADQGAAFTSGTLTKWAADHNIVLEFSTPYHPQS--KVEQKTSVIKCALTKLF

AGRPQKRYPLIPLLQFGINNIPINVDNVIPYQLLFGATPNTPFSGSNLTTKFNSTSANFF-------LSPFGLTSLSRRY

TEGTCLRPHWKKPTKFLQFLNPRTA-IVEHLGQPRTVSIDNLRPTPHQYYCYVSCANTTRVMTSYV-SLILLGIIITASF
ITICRIQWQQTIEAQGPTIFIGPNITVTTIPQGHHWDPYPKPIETKERVVAISQVLLLNTKSILDAAKITDWKTKIKAAM
HKEM----DLADQTYLPMEDSFNQADYEDKLCFDNFGDCYVVDYQEERT-PIKEIIQDQCPPKVWLYFNDPKGGGPLNAT
AFPEDWH-EGKGKIFRPFKMDSFFKEPELVMHLNRTYDAFNSWENDNHSYNCDFQHAKILGLLISKDLHLIPL---YNIY
SLYGICKQTLFK--LLIEKSMNKLIQL-DWNNQQLENGFHLLQTHVITLFESTRYDLQILMA------IQQQLEKIKNQA
RMGHMDCAIVSWNWLQEQLNLTDAQMTLVKETSSARINNVIMGED-----HYEIHIHCELIIPKEIYATNQKLLN--YLV
SQNQHLLKLTIGSQYQYLNYETGQLKYLQVQGCTEDKFLMCDKIKEVPPCGISNNSNCPVYVQTIKDPYLEIIPLQNSSY
IILIE-SNCYIPAYQPIVITTKYPVHCQENVLSPPLLDLPQIHWRLPHLLGTLTELKGYKLKFYNLYDNLQDILDQIRST
LLRYNVHERDLLAWLTQVSKALEDIVPAAVNLISKVTAGITN----LFGGGYSMLFYLIYIFLTGFAIIVLIFIIKIVSW
L

**Reference**


1. Wei X, Chen Y, Duan G, Holmes EC, Cui J. 2019. A reptilian endogenous foamy virus sheds light on the early evolution of retroviruses. Virus Evol 5:vez001.
2. Han GZ, Worobey M. 2012. An endogenous foamy-like viral element in the coelacanth genome. PLoS Pathog 8:e1002790.
3. Aiewsakun P, Katzourakis A. 2017. Marine origin of retroviruses in the early Palaeozoic Era. Nat Commun 8:13954.
4. Katzourakis A, Gifford RJ, Tristem M, Gilbert MT, Pybus OG. 2009. Macroevolution of complex retroviruses. Science 325:1512.
5. Wang J, Han GZ. 2021. A Sister Lineage of Sampled Retroviruses Corroborates the Complex Evolution of Retroviruses. Mol Biol Evol 38:1031-1039.

6. Chen Y, Zhang YY, Wei X, Cui J. 2021. Multiple Infiltration and Cross-Species Transmission of Foamy Viruses across the Paleozoic to the Cenozoic Era. J Virol 95:e0048421.
7. Chen Y, Wei X, Zhang G, Holmes EC, Cui J. 2019. Identification and evolution of avian endogenous foamy viruses. Virus Evol 5:vez049.
8. Wang X, Chen Y, Cui J. 2023. Identification of Cartilaginous Fish Endogenous Foamy Virus Rooting to Vertebrate Counterparts. J Virol 97:e0181622.
